# Molecular Decay of the Tooth Gene Enamelin (*ENAM*) Mirrors the Loss of Enamel in the Fossil Record of Placental Mammals

**DOI:** 10.1371/journal.pgen.1000634

**Published:** 2009-09-04

**Authors:** Robert W. Meredith, John Gatesy, William J. Murphy, Oliver A. Ryder, Mark S. Springer

**Affiliations:** 1 Department of Biology, University of California Riverside, Riverside, California, United States of America; 2 Department of Veterinary Integrative Biosciences, Texas A&M University, College Station, Texas, United States of America; 3 San Diego Zoo's Institute for Conservation Research, Escondido, California, United States of America; Fred Hutchinson Cancer Research Center, United States of America

## Abstract

Vestigial structures occur at both the anatomical and molecular levels, but studies documenting the co-occurrence of morphological degeneration in the fossil record and molecular decay in the genome are rare. Here, we use morphology, the fossil record, and phylogenetics to predict the occurrence of “molecular fossils” of the enamelin (*ENAM*) gene in four different orders of placental mammals (Tubulidentata, Pholidota, Cetacea, Xenarthra) with toothless and/or enamelless taxa. Our results support the “molecular fossil” hypothesis and demonstrate the occurrence of frameshift mutations and/or stop codons in all toothless and enamelless taxa. We then use a novel method based on selection intensity estimates for codons (ω) to calculate the timing of iterated enamel loss in the fossil record of aardvarks and pangolins, and further show that the molecular evolutionary history of *ENAM* predicts the occurrence of enamel in basal representatives of Xenarthra (sloths, anteaters, armadillos) even though frameshift mutations are ubiquitous in *ENAM* sequences of living xenarthrans. The molecular decay of *ENAM* parallels the morphological degeneration of enamel in the fossil record of placental mammals and provides manifest evidence for the predictive power of Darwin's theory.

## Author Summary

Enamel is the hardest substance in the vertebrate body. One of the key proteins involved in enamel formation is enamelin. Most placental mammals have teeth that are capped with enamel, but there are also lineages without teeth (anteaters, pangolins, baleen whales) or with enamelless teeth (armadillos, sloths, aardvarks, pygmy and dwarf sperm whales). All toothless and enamelless mammals are descended from ancestral forms that possessed teeth with enamel. Given this ancestry, we predicted that mammalian species without teeth or with teeth that lack enamel would have copies of the gene that codes for the enamelin protein, but that the enamelin gene in these species would contain mutations that render it a nonfunctional pseudogene. To test this hypothesis, we sequenced most of the protein-coding region of the enamelin gene in all groups of placental mammals that lack teeth or have enamelless teeth. In every case, we discovered mutations in the enamelin gene that disrupt the proper reading frame that codes for the enamelin protein. Our results link evolutionary change at the molecular level to morphological change in the fossil record and also provide evidence for the enormous predictive power of Charles Darwin's theory of descent with modification.

## Introduction

Vestigial characters occur in a degenerate condition as a result of evolutionary reduction from a more elaborated, functional character state in an ancestral species [1]. Darwin [2],[3] identified “vestiges” in human anatomy such as the vermiform appendix that are leftovers from distant evolutionary history, and recognized that vestigial organs are both anticipated and explained by his theory of descent with modification. Other vestiges include remnants of the pelvic girdle and hindlimb within the body wall of baleen whales [4] and eyes that are in various stages of degeneration in cave-dwelling animals [5]. At the genomic level, genes that were functional in an ancestral species but which are no longer under selective constraints are predicted to degrade through an accumulation of mutations [6],[7]. However, previous studies provide limited evidence linking morphological degeneration in the fossil record to molecular decay in the genome owing to the rarity of soft tissue preservation and the prevalence of pleiotropic genes that influence multiple phenotypic traits.

Enamel is the hardest substance found in vertebrates and forms the outer cap of teeth, which comprise the most common and often only preserved remains of extinct mammalian species. Enamel is a derivative of the oral epithelium (ameloblasts), and its development involves secretory and maturation phases that culminate in the formation of an organized network of hydroxyapatite crystals [8]. Multiple extracellular matrix proteins (EMPs) are secreted by mammalian ameloblasts: amelogenin, ameloblastin, and enamelin are structural proteins that direct the formation of hydroxyapatite crystals during the secretory phase of enamel formation; enamelysin and kallikrein 4 are proteinases that process and eventually degrade the structural EMPs during the secretory and maturation phases, respectively. Mutations in the genes for amelogenin (*AMELX)*, enamelin (*ENAM*), enamelysin (*MMP20*), and kallikrein 4 (*KLK4*) are known to cause defects in enamel that are collectively referred to as amelogenesis imperfectas [9]. The three structural EMP genes belong to the secretory calcium-binding phosphoprotein (SSCP) gene family [10], and have an evolutionary history that traces at least as far back as the most recent common ancestor of tetrapods [11],[12]. EMPs that are associated with enameloid formation in chondrichthyans and actinopterygians also belong to the SSCP gene family, but are not orthologous to tetrapod EMPs [12].

Enamelin is the largest EMP in mammals (1104 aa in pig) and is secreted along the mineralization front of developing enamel [13] where it may play a role in crystal elongation [8]. Homozygous *ENAM* knock-in mice show no true enamel [14], but are otherwise normal. The combination of the hardness of enamel and the hypothesized ameloblast-specific expression of enamelin [14] provides a model system for investigating the correlated loss of a phenotypic character (enamel) in the fossil record and the molecular decay of a protein-coding gene (*ENAM*) in the genome.

Most placental mammals have teeth with enamel, but there are also edentulous (toothless) mammals (pangolins, baleen whales, anteaters) and mammals with enamelless teeth (sloths, armadillos, pygmy and dwarf sperm whales, aardvark). Among edentulous mammals, there is evidence for aborted tooth bud development in mysticetes [15], pangolins [16],[17], and anteaters [17]. Rudimentary teeth are best developed in mysticetes, but enamel is not deposited and the tooth buds are degraded and resorbed prior to parturition (reviewed in reference [15]). Given that all edentulous and enamelless mammals ultimately evolved from ancestors with fully mineralized teeth that included an outer enamel cap, we hypothesized that ameloblast-specific *ENAM* would be present in these taxa, albeit nonfunctional and in various states of decay with frameshift mutations and stop codons. To test this hypothesis, we generated and analyzed protein-coding sequences for *ENAM* in all placental orders containing edentulous and/or enamelless taxa. We also used selection intensity estimates for codons (ω) on branches of the tree with functional versus pseudogenic histories to estimate the timing of iterated enamel loss in the fossil record of aardvarks and pangolins. Finally, molecular evolutionary analyses of *ENAM* were employed to predict the occurrence of teeth with enamel in basal representatives of the order Xenarthra (anteaters, sloths, armadillos).

## Results/Discussion

Our sequence alignment (∼4.1 kb; Dataset S1) included more than 80% of the protein-coding region for *ENAM*. Taxon sampling included 20 species that are representative of all placental orders that either lack teeth or have enamelless teeth, 27 additional placental species among which are the closest living relatives to each edentulous/enamelless group, and two marsupial outgroups. The *ENAM* gene tree (Figure S1 and Figure S2) is generally concordant with other molecular phylogenies based on larger data sets [18],[19]. Three of the four major clades of placental mammals (Afrotheria, Xenarthra, Laurasiatheria) were represented in our study and each was recovered with robust support in the *ENAM* gene tree. Further, relationships within both Xenarthra and Cetartiodactyla are generally congruent with other molecular phylogenies [20],[21]. Differences between the *ENAM* gene tree and other molecular studies pertain to nodes that are not well supported by *ENAM* alone and typically require larger data sets with increased taxon sampling to subdivide long branches.

**Figure 1:**
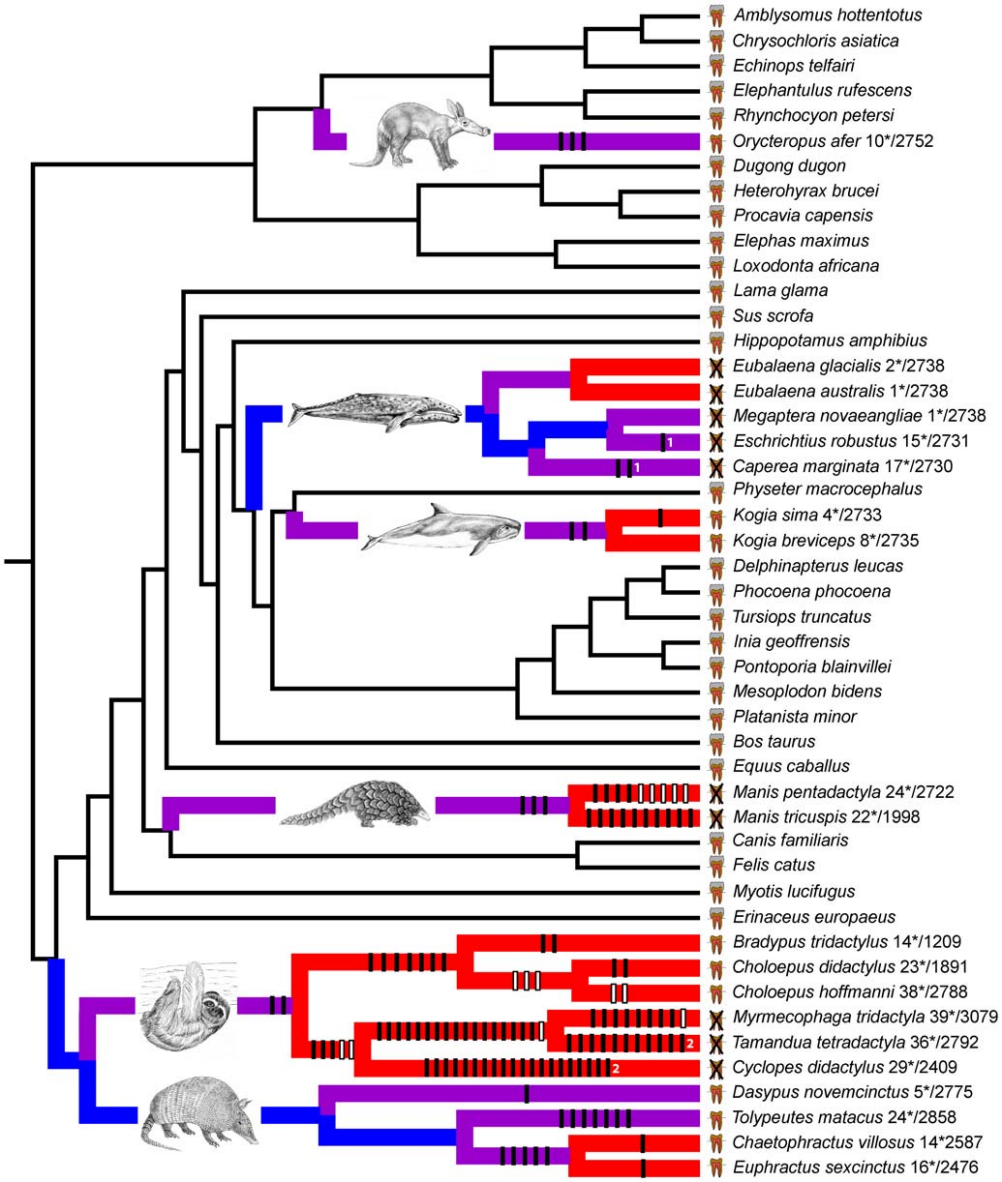
Species tree with frameshift mutations and dN/dS branch coding. Symbols next to taxon names denote taxa having teeth with enamel, taxa having teeth without enamel, and edentulous taxa. Branches are functional (black), pre-mutation (blue), mixed (purple), and pseudogenic (red). Vertical bars on branches represent frameshift mutations (see Table S1). Frameshifts that map unambiguously onto branches are shown in black. Frameshifts shown in white are unique, but occur in regions where sequences are missing for one or more taxa (Figure S7) and were arbitrarily mapped onto the youngest possible branch. Homoplastic frameshifts (deltran optimization) are marked by numbers. Numbers after taxon names indicate the minimum number of stop codons in the sequence (before slashes) and the length of the sequence (after slashes).

**Figure 2:**
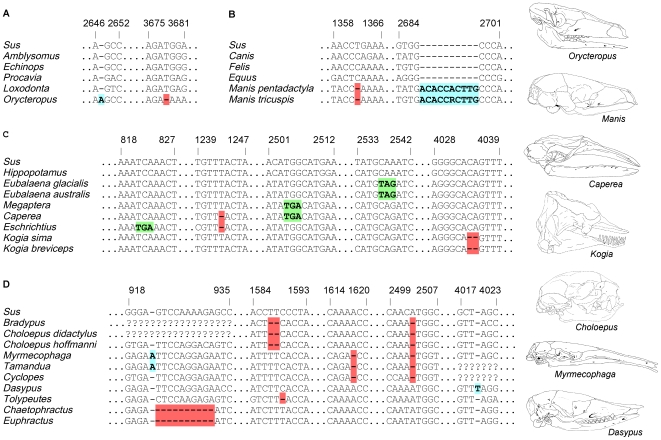
Examples of frameshift mutations and stop codons in edentulous and enamelless taxa. (A) *Orycteropus*. (B) *Manis*. (C) Cetacea. (D) Xenarthra. Frameshift insertions are highlighted in blue, frameshift deletions are highlighted in red, and stop codons are highlighted in green. Also see Figure S5.

Given the concordance of our gene tree to mammalian species trees, we mapped 125 different *ENAM* frameshift mutations onto a composite species tree (Figure 1). Of these, 123 mapped onto the tree without homoplasy and the remaining two required only two character state changes (Figure 1, Table S1). Frameshifts are lacking in all taxa having teeth with enamel whereas 17 of 20 taxa lacking teeth or enamel have at least one frameshift in *ENAM* (Figure 1). Frameshifts are evident in all four placental orders that include edentulous or enamelless taxa (Figure 2).

*ENAM* sequences for edentulous and enamelless taxa were translated in all three reading frames and in every case stop codons were present (Figure 1). Stop codons occur in both of the baleen whale genera (*Eubalaena*, *Megaptera*) that lack frameshift mutations. The combination of frameshifts and stop codons provides evidence that all edentulous and enamelless taxa lack a functional copy of full length *ENAM*. Molecular decay of *ENAM* in these lineages also corroborates the hypothesis that enamelin exhibits ameloblast-specific gene expression [14].

We calculated dN/dS ratios (ω) for *ENAM* after dividing phylogenetic branches into four categories [functional, pre-mutation, mixed (functional + pseudogene), and pseudogene] based on ancestral reconstructions of the presence or absence of enamel, and the distribution of frameshifts and stop codons in *ENAM* sequences (Figure 1, Figure S3). Functional branches lead to internal nodes or extant species with teeth having enamel and are expected to have evolved under selective constraints with the lowest ω values unless there is extensive positive selection. Pre-mutation, mixed, and pseudogene branches lead to internal nodes or extant species that lack enamel. Pre-mutation branches lack frameshifts or stop codons in the region of *ENAM* that was sequenced. Mixed branches are descended from pre-mutation branches and contain the first detected occurrence of a frameshift or stop codon. Mixed branches may be entirely pseudogenic if undetected frameshifts occurred on an earlier (pre-mutation) branch; alternatively, mixed branches may record a history of evolution under functional constraints combined with pseudogenic evolution if the first frameshift or stop codon occurred on the mixed branch. Pseudogene branches occur after mixed branches and are expected to have ω values near the neutral value of 1 given the absence of purifying or positive selection.

Results of dN/dS analyses confirm that functional branches have evolved under purifying selection (ω = 0.51) and that pseudogene branches have evolved as predicted for pseudogenes (ω = 1.02). The pseudogene value of 1.02 agrees with the null hypothesis that *ENAM* evolved under neutral constraints (ω = 1) based on a χ^2^ test (0.75<P<0.90). Estimated values for pre-mutation and mixed branches were 0.83 and 0.98, respectively, and are compatible with the hypothesis that *ENAM* has evolved under progressively more relaxed constraints on branches leading to species that are edentulous or enamelless. The ω value for mixed branches is only slightly less than for pseudogene branches, which suggests that these branches have histories that are almost entirely pseudogenic.

The abundance of mammalian teeth in the fossil record provides information on the timing and sequence of events leading to enamel and tooth loss in different mammalian lineages. Complementary data from molecular evolutionary analyses of *ENAM* provide information on missing segments of the fossil record. Together, as discussed below, the fossil record and the genome facilitate predictive interplay and inform our understanding of the evolutionary history of enamel degeneration in a way that is not possible based on fossils or molecular sequences alone.

**Figure 3:**
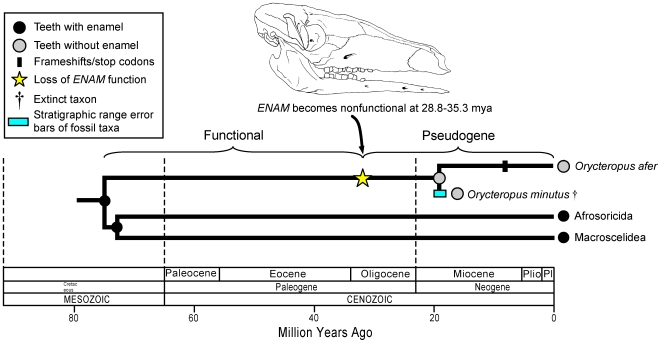
A synthetic interpretation of the history of enamel degeneration in Tubulidentata (aardvarks) based on fossils, phylogenetics, molecular clocks, frameshift mutations, and dN/dS ratios. Estimates for the timing of the transition from functional gene to pseudogene are based on decomposition of mixed dN/dS history into two distinct episodes of evolution under purifying selection and neutral evolution, respectively. The divergence time between Tubulidentata and Afrosoricida (golden moles, tenrecs) + Macroscelidea (elephant shrews) is from Murphy and Eizirik [23]. The age of the oldest aardvark, *O. minutus*, is from Milledge [25].

Teeth in the extant aardvark, *Orycteropus afer*, lack both enamel and a central pulp cavity and are composed of ∼1500 thin, hexagonal tubes of dentin that are bound together by cementum [22]. Whereas molecular data suggest that the aardvark lineage diverged from its closest living relatives (Afrosoricida, Macroscelidea) ∼75.1 million years ago in the Cretaceous [23], the oldest fossil aardvarks are *O. minutus* (19 mya) and *Myorycteropus africanus* (18 mya) from the early Miocene of Kenya and also lack enamel [24],[25]. The combination of molecular and paleontological data thus suggests that enamel was lost in the aardvark lineage after aardvarks split from their nearest extant relatives 75 million years ago but before the early Miocene when aardvarks first appear in the fossil record. We estimated the value of ω for *ENAM* at 0.75 for the aardvark branch and then calculated the fraction of functional versus pseudogenic history for *ENAM* on this branch by comparing its ω value with ωvalues that were calculated for functional and pseudogene branches (see Methods). Calculations were performed using two different assumptions. First, functional and pseudogenic segments of the mixed branch were assumed to have equal synonymous substitution rates [26],[27]. Second, the rate of synonymous substitution on the functional segment of the mixed branch was assumed to be 70% of the rate of synonymous substitution on the pseudogenic segment on the mixed branch [28]. Our estimates for the transition from functional gene to pseudogene for aardvark *ENAM* range from 28.8 mya (two synonymous rates) to 35.3 mya (one synonymous rate) and are ∼10-16 million years older than the first fossil aardvarks. Our analyses predict the occurrence of enamelless fossil aardvarks that are much older than both *O. minutus* and *M. africanus* (Figure 3).

**Figure 4:**
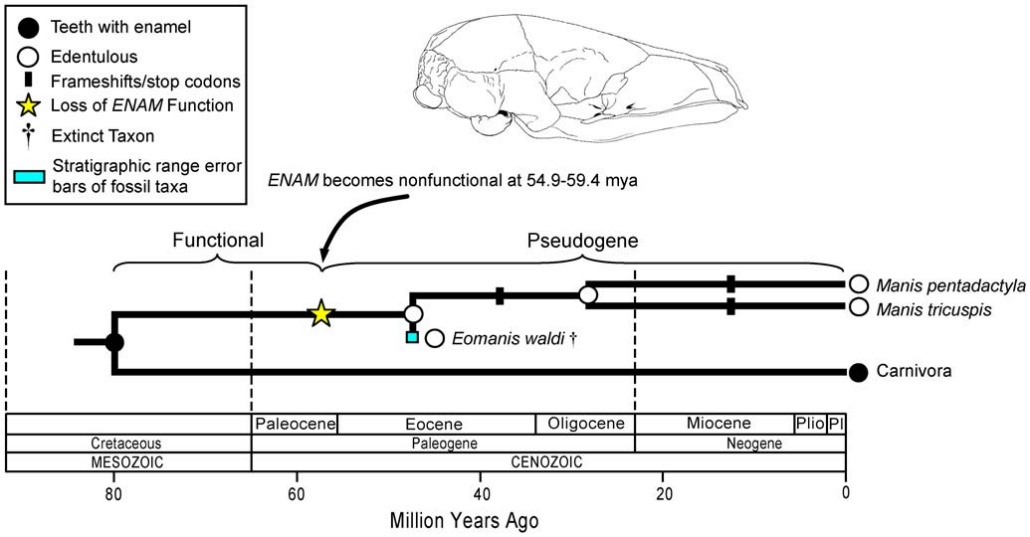
A synthetic interpretation of the history of enamel degeneration in Pholidota (pangolins) based on fossils, phylogenetics, molecular clocks, frameshift mutations, and dN/dS ratios. Estimates for the timing of the transition from functional gene to pseudogene are based on decomposition of mixed dN/dS history into two distinct episodes of evolution under purifying selection and neutral evolution, respectively. The divergence time between Pholidota and Carnivora (79.8 mya) is from Murphy and Eizirik [23]. The divergence time between *Manis pentadactyla* and *M. tricuspis* (27.9 mya) is based on molecular dating analyses with *ENAM* (see Methods). The age of the oldest pangolin, *Eomanis* from the Eocene Messel deposits of Germany, is ∼47 mya [29].

Numerous frameshifts were reconstructed on the internal branch leading to living pangolins (*Manis*) and support the hypothesis that *ENAM* was rendered nonfunctional on this branch (Figure 1). The internal branch leading to crown-group *Manis* extends from 79.8 mya [23] to 27.9 mya (see Methods). The earliest definitive pangolin is *Eomanis* from the Eocene Messel deposits of Germany, which have been dated at ∼47 mya [29]. *Eomanis* was edentulous as are modern pangolins. The occurrence of an early pangolin without teeth is consistent with our finding that *ENAM* became a pseudogene on the stem pangolin branch and further suggests that *ENAM* has been a pseudogene since at least the Eocene. Given that the branch leading to crown-group pangolins represents ∼52 million years of evolution and that the value of ω on this branch is 0.82, we used the aforementioned method to estimate that the transition from functional gene to pseudogene occurred 54.9 (two synonymous rates) to 59.4 (one synonymous rate) mya, which is ∼8 to 12 million years older than *Eomanis*. We therefore predict the occurrence of edentulous, or at least enamelless, fossil pangolins in the Paleocene (Figure 4).

Extinct palaeanodonts have been associated with pangolins [30],[31], xenarthrans [32],[33], or both groups [34]–[36]. Palaeanodonts first appear in the early Paleocene of North America and show a mixture of xenarthran and pholidotan features [37]. The earliest forms have teeth with enamel, but palaeanodonts show trends toward reduction and loss of postcanine teeth from back to front, and loss of enamel on the postcanine teeth [37]–[39]. Rose *et al*. (Figure 8.4 in [37]) hypothesized that Palaeanodonta and Pholidota are either sister taxa that split in the latest Cretaceous or that Pholidota evolved from within Palaeanodonta in the early Eocene. The former hypothesis implies that tooth reduction and loss, as well as enamel loss on the postcanine teeth, occurred independently in palaeanodonts and pangolins, whereas these changes may be shared derived features uniting metacheiromyid palaeanodonts and pangolins if the latter hypothesis is correct. Our estimates for the transition from functional *ENAM* to pseudogene on the basal pangolin branch (54.9–59.4 mya) suggest that ancestral pholidotans without enamel were contemporaneous with, rather than descended from, late Paleocene and early Eocene metacheiromyids such as *Palaeanodon* that exhibited a reduced postcanine dental arcade, but retained a functional copy of the *ENAM* gene as inferred by the presence of canines with enamel. The combination of fossil and molecular evolutionary evidence is thus more compatible with the hypothesis that palaeanodonts and pholidotans are sister taxa than the hypothesis that pangolins are derived from within Metacheiromyidae.

**Figure 5:**
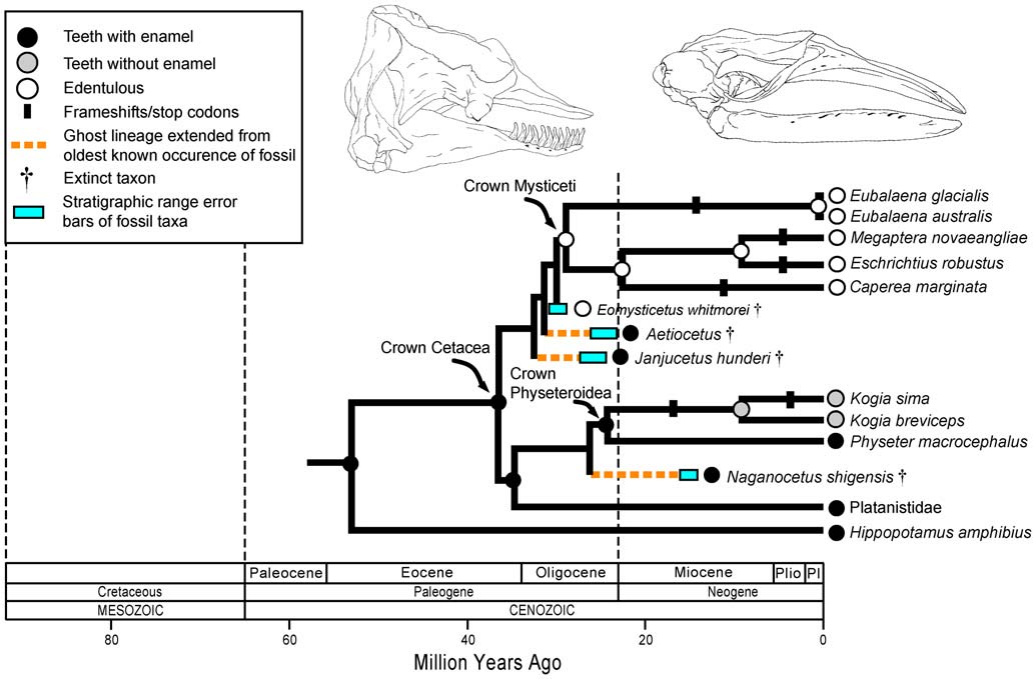
A synthetic interpretation of the history of enamel degeneration in Cetacea based on fossils, phylogenetics, molecular clocks, frameshift mutations, and dN/dS ratios. The common ancestor of Cetacea was reconstructed as having teeth with enamel based on the occurrence of teeth with enamel in stem cetaceans, stem mysticetes, and stem odontocetes. The common ancestor of Mysticeti was reconstructed as toothless based on (1) the fossil record, which records the transition from ancestral forms that possessed teeth but not baleen (e.g., *Janjucetus*), to intermediate forms with teeth and baleen (e.g., *Aetiocetus*), to forms that are fully edentulous (e.g., *Eomysticetus*) and (2) a dN/dS ratio for *ENAM* in crown mysticetes that agrees with the null hypothesis of neutral evolution (dN/dS = 1). The common ancestor of physeteroids was reconstructed as having teeth with enamel based on the presence of enamel-capped teeth in stem physeteroids (e.g., *Naganocetus*) and *Physeter*. Cladistic relationships and divergence times among extant taxa are based on McGowen *et al.* [69]. Cladistic relationships of basal mysticetes and basal physeteroids are based on Deméré *et al.* [15] and Bianucci and Landini [40], respectively. Frameshift mutations and stop codons were mapped using deltran optimization. All extant mysticete lineages that were sampled have at least one frameshift or stop codon, but none are shared by all mysticetes. Acctran optimization suggests that some homoplastic frameshifts and stop codons within Mysticeti may have resulted from lineage sorting of ancestral polymorphisms [15].

Cetacea includes both edentulous (Mysticeti) [15] and enamelless (*Kogia*) [40] taxa. Early mysticete fossils record the transition from ancestral forms that possessed teeth, but not baleen, to intermediate forms with teeth and baleen, to forms that are fully edentulous [15],[41]. This pattern suggests that purifying selection on enamel-specific genes was relaxed on the stem mysticete branch. The five mysticetes examined here all exhibit frameshifts and/or stop codons in *ENAM*, which is indicative of pseudogenic evolution, although no frameshifts or stop codons were found that unite all mysticetes. This result agrees with the findings of Deméré *et al.* [15] that the ameloblastin gene (*AMBN*) and a shorter segment of *ENAM* (∼530 bp) both contain frameshift mutations that are shared by one or more mysticete taxa, but none in common to all extant mysticetes. Whereas deltran optimization (Figure 1) suggests an independent origin of the same frameshift mutation in *Eschrichtius robustus* and *Caperea marginata*, acctran optimization (not shown) suggests an earlier origin of the frameshift in the common ancestor of *E. robustus*, *C. marginata*, and *Megaptera novaeangliae*, followed by loss of the frameshift in *M. novaeangliae*. The acctran optimization scenario is compatible with the gain of an ancestral polymorphism on the branch leading to these taxa, followed by subsequent lineage sorting that resulted in loss of the frameshift in *M. novaeangliae* [15]. When all crown mysticete branches were treated as a separate category in dN/dS analyses (Methods), the result was in agreement with the null hypothesis that *ENAM* evolved as a pseudogene (i.e., ω = 1) based on a χ^2^ test (0.10<P<0.25). Thus, it appears that *ENAM* was released from selective constraints in crown mysticetes as predicted by the basal position of the archaic toothless mysticete, *Eomysticetus whitmorei* (Figure 5) [15]. If we assume that *ENAM* evolved as a pseudogene in crown-group mysticetes, which as a group are characterized by generation times and lifespans that equal or exceed those of most other mammals, then the neutral rate of nucleotide substitution is only 3.3×10^−4^ substitutions/site/myr, which is almost an order of magnitude lower than Kumar and Subramanian's [27] estimate of the average mammalian genome rate of 2.2×10^−3^ substitutions/site/myr based on an analysis of fourfold degenerate sites.

Stem physeteroids (sperm whales) are known from the Miocene and had teeth with enamel [40]. *Physeter* and *Kogia* are the only living genera of sperm whales and the teeth in adults belonging to these genera lack enamel. A caveat is that thin, prismless enamel has been observed on the unerupted teeth of *P. macrocephalus* (giant sperm whale) [42]. Our results provide support for loss of the intact enamelin protein in both *Kogia* species, but not in *P. macrocephalus* (Figure 5, Text S1).

**Figure 6:**
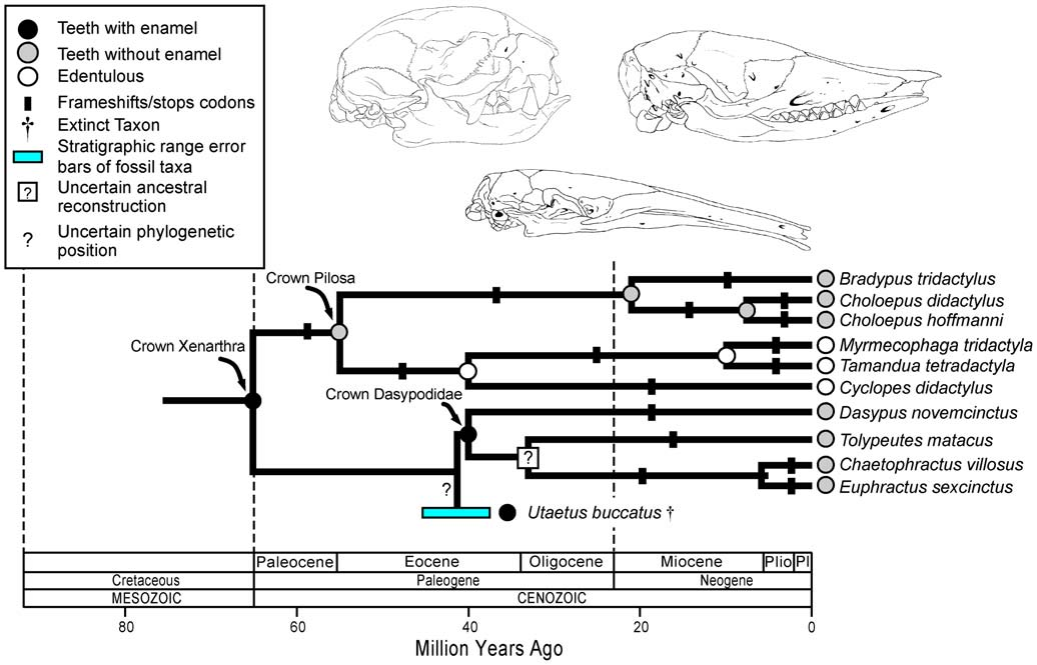
An integrated interpretation of the history of enamel degeneration in Xenarthra based on fossils, phylogenetics, molecular clocks, frameshift mutations, and dN/dS ratios. No definitive stem xenarthrans have been described, but the common ancestor of Xenarthra was reconstructed as having teeth with enamel based on (1) purifying selection on the stem xenarthran branch (ω = 0.48), (2) a reconstructed ancestral *ENAM* sequence that contains no frameshifts and no stop codons, (3) at least one crown-group xenarthran (*Utaetus*) with enamel, which would be improbable to re-evolve if the common ancestor of Xenarthra lacked enamel and had a nonfunctional copy of *ENAM*, and (4) genomics observations. The common ancestor of Pilosa was reconstructed as having teeth without enamel based on the occurrence of shared frameshifts in *ENAM* on the stem pilosan branch. The dN/dS ratio in crown clade Dasypodidae agrees with the neutral evolution (dN/dS = 1) hypothesis. There are also frameshifts that result in premature stop codons in all dasypodid *ENAM* sequences; however, none are shared by all four dasypodids that were sampled in our analysis. We reconstructed the ancestor of living Dasypodidae as having enamel, which may have been very thin as in *Utaetus*, based on the absence of shared frameshift mutations in protein-coding sequences of *AMELX*, *AMBN*, *ENAM*, and *MMP20*. The sister group relationship and divergence time between *Choloepus hoffmanni* and *C. didactylus* is based on analyses with *ENAM* (Figure S1, Figure S2; Methods). All other relationships and divergence times are based on Figure 1 of Delsuc *et al.* [20]. *Utaetus* is derived from Casamayoran age deposits, which are thought to be ?middle to late Eocene in age [75],[76].

Xenarthrans comprise one of the major clades of placental mammals [18] and the stem xenarthran branch represents ∼30 million years of evolution [43]. Even with this long history there are no described fossils that are definitive basal xenarthrans [37]. Almost all living and fossil xenarthrans, which are crown taxa, either lack teeth or have teeth without enamel. The only clear-cut exception is *Utaetus*, a member of Cingulata (armadillos and extinct relatives) from the Casamayoran (Eocene) that had thin, prismatic enamel (Text S1) [44]. The occurrence of enamel in *Utaetus* suggests that enamel was lost independently in pilosans (anteaters, sloths) and in cingulatans. An alternative hypothesis is that *Utaetus* re-evolved enamel after earlier loss in the common ancestor of Xenarthra (Figure S4). We estimated the value of ω for the stem xenarthran branch to determine if *ENAM* evolved under purifying selection or as a pseudogene on this branch. The estimated value (0.48) is similar to that estimated for functional branches (0.51) and implies that *ENAM* evolved under purifying selection on the stem xenarthran branch, concordant with the hypothesis that enamel was lost independently in Pilosa and in Cingulata. Ancestral reconstructions based solely on the phenotypes of extant taxa (Figure S3) imply that enamel was lost on the stem xenarthran branch, but we predict that all stem xenarthran fossils will have teeth with enamel (Figure 6) based on (1) an estimated value of ω on the stem xenarthran branch that is similar to the estimated value of ω for functional branches elsewhere on the tree, (2) the absence of frameshifts on the stem xenarthran branch, (3) the occurrence of enamel in a fossil armadillo (*Utaetus*), which would be difficult to regain if enamel had been lost earlier in evolution, and (4) genomics observations on *AMELX*, *AMBN*, *MMP20*, and *ENAM* in *Dasypus novemcinctus*, which preclude the possibility of unique, homoplasy-free frameshifts in these genes in the common ancestry of Xenarthra (see below).

Unambiguous fossils of Pilosa are crown rather than stem [37] and the fossil record does not speak directly to whether enamel was lost on the branch leading to extant pilosans. However, the occurrence of 2–4 frameshifts on the stem pilosan branch implies that *ENAM* transitioned from a functional gene to a pseudogene after Pilosa diverged from Cingulata ∼65 mya, but before Folivora (sloths) separated from Vermilingua (anteaters) ∼55 mya [20] (Figure 6).

In Cingulata, all extant dasypodids (armadillos) that we examined have frameshifts and premature stop codons, suggesting that *ENAM* is a decaying pseudogene in these taxa. However, the only shared frameshifts were in *Euphractus* and *Chaetophractus*, which suggests the possibility that enamel was lost independently in different armadillo lineages. As a further test of this hypothesis, we reconstructed the complete coding sequences for the enamelin (*ENAM*), amelogenin (*AMELX*), ameloblastin (*AMBN*), and enamelysin (*MMP20*) genes in *Dasypus novemcinctus* based on Ensembl 51 scaffolds and trace files (including inspection of chromatograms). There are no frameshifts or stop codons in the coding sequences of *AMELX*, *AMBN*, and *MMP20* in *D. novemcinctus*, and the only frameshift in the Ensembl sequence for *ENAM* is identical to the one that we discovered for *D. novemcinctus* (Figure 2, Figure S5, and Figure S6). These observations preclude the occurrence of unique, homoplasy-free frameshifts in *AMELX*, *AMBN*, *MMP20*, or *ENAM* in the common ancestry of either Xenarthra or Dasypodidae and are consistent with the hypothesis that enamel was lost independently in Pilosa and also in more than one dasypodid lineage. When crown-group dasypodid branches were treated as a separate category in dN/dS analyses with *ENAM*, the pseudogene hypothesis of neutral evolution (ω = 1) could not be rejected (0.05<P<0.10), which suggests that *ENAM* has evolved as a pseudogene over most of the history of Dasypodidae even though the protein-coding regions of four EMPs appear to have been intact in the most recent common ancestor of Dasypodidae (Figure 6).

Whereas our analyses provide strong support for multiple instances of tooth loss, enamel loss, and *ENAM* degeneration in placental mammals, they provide no compelling evidence for the re-evolution of enamel following degeneration of the *ENAM* gene on an earlier branch of the tree. This finding is consistent with Dollo's law, which may be stated as follows: complex organs, once lost, can never be regained in exactly the same form [45, p. 1441]. Dollo's law implies that genes or developmental pathways released from selective constraints will rapidly become nonfunctional [46]. Marshall *et al.* [46] showed that there is a significant probability over evolutionary time scales of 0.5 to 6 million years for successful reactivation of neutrally evolving coding sequences in the range of 0.5 to 2.0 kb, but that resurrection of genes of this length that have evolved neutrally for >10 million years is highly improbable. In the case of a three kb exon (e.g., exon 10 of *ENAM*), the probability of survival after one million years of neutral evolution ranges from 0.65 to 1.6×10^−17^ based on calculations using Marshall *et al.*'s [46] equation 4 and a range of substitution rates, frameshift accumulation rates, and different values of *f*, where *f* is the probability that a substitution will not result in loss of function. Even in the case of very slow substitution and frameshift accumulation rates (i.e., mysticete rates), and a high value of *f* (i.e., 0.7) [46], the probability that a 3 kb exon will remain functional after ten million years is only 0.014. Our finding that enamel has not been regained in any placental mammal lineage is consistent with these predictions for *ENAM*'s rapid demise in the absence of selective constraints.

Among other vertebrates, Kollar and Fisher [47] performed experiments with recombinant mouse molar mesenchyme and chick dental epithelium that resulted in “hen's teeth” consisting of a dentine cone covered by enamel matrix proteins. This result implies that chick dental epithelium has retained the genetic potential to develop until the last developmental stage (enamel matrix deposition) for 80 to 100 million years. However, possible contamination of mouse mesenchyme by mouse epithelium makes this interpretation uncertain (reviewed in [48]). Further, Sire *et al.* [48] report that all of the genes encoding dental-specific proteins in chicken, including *ENAM*, have either disappeared from the genome or are pseudogenes. Oral teeth were lost in the common ancestor of cypriniform fishes at least 50 million years ago, possibly in conjunction with the evolution of suction feeding, but have never been regained [49]. The group has subsequently diversified into ∼3000 extant species that exhibit a wide range of feeding modes, but the loss of oral teeth may have constrained the evolution of large fish-eating forms, which are rare and may be less efficient predators than teleosts that retain oral teeth [50],[51]. There are examples of the reacquisition of specific teeth, including the polymorphic occurrence of the second lower molar in *Lynx lynx* [52], and of sets of teeth such as the third basibranchial teeth in centrarchid fishes [53]. However, examples of the reacquisition of individual teeth or sets of teeth only occur in the context of organisms that retain other teeth in the oropharynx [54].

Darwin [2] stated that “the existence of organs in a rudimentary, imperfect, and useless condition, or quite aborted, far from presenting a strange difficulty, as they assuredly do on the ordinary doctrine of creation, might even have been anticipated, and can be accounted for by the laws of inheritance.” Our analysis of *ENAM* pseudogenes is the first study to combine information from living taxa, the fossil record, phylogenetics, molecular sequences, and selection intensity estimates for codons to make reciprocal predictions about patterns of molecular decay and the parallel loss of a phenotypic character in the fossil record. Given ancestral species with enamel-capped teeth, we correctly predicted the occurrence of vestigial copies of *ENAM* in four different placental orders with edentulous or enamelless species. dN/dS methods then allowed us to predict the timing of iterated enamel loss in the fossil record of aardvarks and pangolins and the presence of enamel in extinct, basal xenarthrans that may reside in the rock record, but have not yet been described by paleontologists. The molecular decay of *ENAM* mirrors the morphological degeneration of enamel in edentulous and enamelless taxa and provides manifest evidence for the predictive power of Darwin's theory.

## Methods

### Gene Sequences

Exon 10 [55] is the longest exon in *ENAM* (2838 base pairs in *Sus*) and codes for >80% of the total length of the secreted protein after removal of the signal peptide. PCR was used to amplify ∼95% of exon 10 in four overlapping segments. Primer sequences are enumerated in Figure S7. All segments were amplified with Invitrogen *Taq* DNA polymerase in 50 µl reactions with the following thermal cycling parameters: initial denaturation at 94°C for 2 minutes; 35 cycles of 1 minute at 94°C (denaturation), 1 minute at 50°C (annealing), and 1 minute at 72°C (extension); and a final extension at 72°C for 10 minutes. Fully nested or half nested PCR reactions, using the aforementioned PCR regime, were performed when the primary PCR reaction did not yield a usable product. One µl of the original PCR product was used as the template DNA in the 50 µl nested PCR reaction. PCR products were run out on a 1% agarose gel, excised, and cleaned with the Bioneer AccuPrep Gel Purification Kit. Cleaned PCR products were sequenced in both directions at the UCR Core Instrumentation Facility using an automated DNA sequencer (ABI 3730xl). Contigs were assembled using Sequencher 4.1. In cases where PCR products did not sequence directly, they were cloned using the CloneJET PCR Cloning Kit (Fermentas) and Top10 chemically competent cells. Transformed cells were grown overnight on SOB agar medium at 37°C. Three to five clone colonies were then picked and used as the template DNA in 50 µl PCR reactions using vector specific primers. PCR amplification, cleaning, and sequencing protocols were identical to the aforementioned regime except that the annealing temperature was 60°C. Sequences for cloned PCR products are consensus sequences based on data from at least three clones. Some xenarthran and *Manis* segments failed to amplify (Figure S8). Genbank Accession numbers for new *ENAM* sequences for 46 taxa are GQ354839-GQ354884; sequences for *Bos*, *Canis*, and *Monodelphis* were obtained from Ensembl 51.

### DNA Alignments

DNA sequences were aligned with Clustal X 2.0.9 [56] using default settings and then manually adjusted using Se-Al [57]. Two alignments were generated. The first alignment (4089 bp; Dataset S1), which was used for phylogenetic analyses and visual discovery of frameshift mutations, included complete sequences for all taxa. Frameshift mutations were mapped onto the composite species tree (Figure 1) using deltran parsimony optimization. The second alignment (2760 bp; Dataset S2) was used in dN/dS analyses with PAML and omitted marsupial sequences, all frameshift insertions (i.e., insertions not in multiples of three), all insertions that were unique to one taxon, and all insertions that were unique to edentulous or enamelless taxa. Stop codons were recoded as missing.

### Phylogenetic Analyses

Modeltest 3.06 [58] was used to select the model of molecular evolution (GTR + Γ) that was implemented in ML and Bayesian analyses with the 4089 bp alignment. RAxML7.0.4 [59] and MrBayes v3.1.1 [60],[61] were used to perform the ML and Bayesian analyses, respectively. RAxML analyses employed 500 replicates, randomized MP starting trees, and the fast hill-climbing algorithm; all other free parameters were estimated. The Bayesian analyses used the default priors, random starting trees, and eight chains (seven hot, one cold), and were terminated when the standard deviation of split frequencies for two different simultaneous analyses fell below 0.01 (2.03 million post-burnin generations). Standard deviation of split frequency calculations were performed on the fly after discarding 25% of each chain as burnin. Post-burnin trees were sampled every 1000 generations (i.e., 2030 trees were sampled from each analysis).

### dN/dS Analyses

PAML 4.2 [62] was used to estimate the ratio (ω) of the nonsynonymous substitution rate (dN) to the synonymous substitution rate (dS) using a composite species tree (Figure 1) and the PAML alignment after recoding stop codons as missing data. The composite species tree is based on Springer *et al.* [19] for interordinal relationships, Delsuc et al. [20] for xenarthrans, and Gatesy [63] for cetartiodactyls. Codeml [62] with the branch model was implemented to estimate dN/dS ratios (ω) for four different branch categories (functional, pre-mutation, mixed, pseudogene) (Figure 1 and Figure S3). Functional branches lead to terminal nodes (i.e., extant taxa) having enamel or internal nodes that were reconstructed as having enamel (Figure S3) and are expected to have evolved under purifying selection with ω values<1. Pre-mutation, mixed, and pseudogene branches lead to terminal nodes without enamel or internal nodes that were reconstructed as not having enamel (Figure S3). Pre-mutation branches predate the first detected occurrence of a stop codon or frameshift in *ENAM*; these branches have uncertain functional versus pseudogene histories and may have evolved under purifying selection or alternatively under relaxed constraints if enamel was already lost or in a state of degeneration. Mixed branches contain the first detected occurrence of a frameshift mutation or stop codon in *ENAM*. Mixed branches may be entirely pseudogenic if undetected frameshift mutations (i.e., in one of the remaining short exons that comprise the *ENAM* gene structure that we did not sample here) occurred on an earlier pre-mutation branch; alternatively, mixed branches may reflect a history of evolution that includes a period of evolution under functional constraints followed by a period of pseudogenic evolution if the first frameshift mutation or stop codon in *ENAM* occurred on the mixed branch. Pseudogene branches post-date the first detected occurrence of a frameshift or stop codon on an earlier branch and are expected to have evolved at the neutral rate (ω = 1). In addition to analyses that recognized these four categories of branches, we performed additional analyses that added a fifth branch category for (a) the branch leading to *Orycteropus*, (b) the branch leading to *Manis* species, (c) crown-group mysticete branches, (d) the stem branch leading to crown Xenarthra, and (e) crown-group dasypodid branches, respectively. Codon sites with ambiguous data (i.e., missing data, gaps) were included in the analyses. We used χ^2^ tests to compare the estimated number of nonsynonymous and synonymous substitutions (from PAML) with the expected number of nonsynonymous and synonymous substitutions according to a neutral model of evolution with ω = 1. PAML estimates for the number of nonsynonymous and synonymous sites in the alignment were 2004.6 and 755.4, respectively. These estimates were used to calculate expected numbers of nonsynonymous and synonymous changes. In the case that examined all pseudogene branches in the four-category analysis, estimated numbers of nonsynonymous and synonymous changes were 780.8 and 287.9, respectively, whereas expected numbers of nonsynonymous and synonymous changes were 776.2 and 292.5, respectively. In the case of crown-group mysticetes, estimated numbers of nonsynonymous and synonymous changes were 66.3 and 16.3, respectively, whereas expected numbers of nonsynonymous and synonymous changes were 60.0 and 22.6 for ω = 1. Finally, estimated numbers of nonsynonymous and synonymous changes were 308.7 and 93.0, respectively, for crown-group dasypodids, whereas expected numbers or nonsynonymous and synonymous changes were 291.8 and 109.9 for ω = 1. All PAML analyses were run with the CodonFreq = 3 option in PAML.

### Molecular Dating Analyses

The relaxed molecular clock method implemented in *Multidivtime* (version 9-25-03) [64]–[66] was used to estimate divergence times for several pairs of taxa that lack published molecular estimates of divergence times: *Manis tricuspis* and *M. pentadactyla*; *Choloepus didactylus* and *C. hoffmanni*; *Heterohyrax brucei* and *Procavia capensis*; and *Amblysomus hottentotus* and *Chrysochloris asiatica*. We used the phylogeny in Figure 1 with the following constraints: (1) minimum of 50 mya and maximum of 63 mya for the split between Feliformia and Caniformia [67]; (2) minimum of 54 mya and maximum of 65 mya for the base of Paenungulata [67]; (3) minimum of 52 mya for the base of the hippo and cetacean clade [68] and a maximum of 65 mya given uncertain relationships between cetartiodactyls and Paleocene mesonychids [21].

### Calculations of Substitution Rates in Mysticetes

We calculated the neutral substitution rate in crown-group mysticetes as 82.6 substitutions/2760 nucleotide sites/90 myr = 3.3×10^−3^ substitutions/site/myr, where 82.6 is the PAML estimate for the total number of substitutions in *ENAM* on crown-group mysticete branches, 2760 is the number of nucleotide sites in the PAML alignment, and 90 myr is the total duration of crown-group mysticete branches based on McGowen *et al.* [69]. Similarly, we calculated the rate of frameshift accumulation in mysticetes as 2 frameshifts/2.76 kb/90 myr = 0.0081 frameshifts/kb/myr, where 2 is the minimum number of frameshifts in mysticete *ENAM* (acctran reconstruction).

### Resolving Mixed Branches into Functional and Pseudogenic Components

The mixed branches leading to *Orycteropus* and to the two species of *Manis* were assumed to have mixed histories that included both functional and pseudogenic components. For these branches, the lengths of time that *ENAM* was functional versus pseudogenic were estimated under two different assumptions. First, we assumed that rates of synonymous substitution are neutral and equal on functional and pseudogene branches [26],[27]. Second, we assumed that the rate of synonymous substitution on functional branches is non-neutral and is 70% of the rate of synonymous substitution on pseudogene branches following Bustamante *et al*. [28]. Possible reasons for a lower rate of synonymous substitution on functional branches include constraints associated with codon usage, splicing, and mRNA stability [70].

For a single rate of synonymous substitution on functional and pseudogenic branches, the following equation describes the relationship between the lengths of time that *ENAM* was functional versus pseudogenic on the mixed branch:
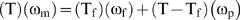
(1)where T is the length of the mixed branch in millions of years, ω_m_ is the dN/dS estimate for the mixed branch, T_f_ is the amount of time that *ENAM* was functional on the mixed branch, ω_f_ is the dN/dS estimate for functional branches, T_p_  =  (T − T_f_) is the amount of time that *ENAM* was pseudogenic on the mixed branch, and ω_p_ is the dN/dS estimate for pseudogenic branches. T_f_ and T_p_ are then obtained by rearrangement and substitution:
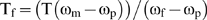
(2)

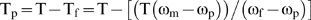
(3)


In the case of two different synonymous substitution rates, one for functional branches and the other for pseudogenic branches, the lengths of time that *ENAM* was functional versus pseudogenic were estimated using the following equations:
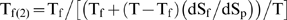
(4)

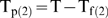
(5)where T_f(2)_ is the amount of time that *ENAM* was functional on the mixed branch when there are two synonymous rates, T_p(2)_ is the amount of time that *ENAM* was pseudogenic on the mixed branch when there are two synonymous rates, dS_f_ is the synonymous (non-neutral) rate of substitution on the functional segment of the mixed branch, dS_p_ is the synonymous (neutral) rate of substitution on the pseudogenic segment of the mixed branch, and T_f_ is from equation 2. The denominator in equation 4 adjusts for differences in rates of synonymous substitution on functional and pseudogenic segments of the mixed branch, and also introduces a normalization correction so that T  =  T_f(2)_ + T_p(2)_. In the case of dS_f_  =  dS_p_, the denominator in equation 4 equals 1 and the equation reduces to T_f(2)_  =  T_f_.

Our approach for dating the transition from functional gene to pseudogene is conceptually related to methods that are based on the overall substitution rate [71] and the rate of indel accumulation [72],[73], respectively.

### Ancestral Reconstructions

Ancestral reconstructions for the presence or absence of enamel (Figure S3) were performed with SIMMAP Version 1.0 B2.3.2 [74].

### Calculations of Survival Time under Neutral Evolution

Equation 4 from Marshall *et al.* [46] was used to estimate the probability that a three kb exon will remain functional after one million years of neutral evolution. Calculations were performed using the very slow rate of nucleotide substitution that we calculated for mysticetes (r_s_ = 3.3×10^−4^ substitutions per site per myr) and Marshall *et al*.'s [46] slow, intermediate, and fast rates of nucleotide substitution (*r*_s_ = 1×10^−3^, 5×10^−3^, and 10×10^−3^ substitutions per site per myr); very slow (i.e., mysticetes), slow, intermediate, and fast rates of frameshift accumulation (*r*_f_ = 0.0081, 0.014, 0.093, and 0.287 frameshifts per kb per myr); and with two different values of *f* (0.3, 0.7), where *f* is the probability that a substitution will not result in loss of function and the chosen values represent endpoints of Marshall *et al*.'s [46] “realistic range” for this variable.

## Supporting Information

Dataset S1 Complete nexus alignment of DNA sequences for *ENAM.*
(0.32 MB TXT)

Dataset S2 Complete nexus alignment of DNA sequences used in PAML analyses.
(0.21 MB TXT)

Figure S1 ML phylogram based on 4089 bp alignment (Dataset S1). Marsupial outgroups are not shown.
(1.72 MB TIF)

Figure S2 Maximum posterior probability tree with Bayesian posterior probabilities above branches and ML bootstrap support percentages below branches. Bayesian and ML analyses were performed with the 4089 bp alignment (Dataset S1).
(1.81 MB TIF)

Figure S3 Ancestral state reconstructions (enamel present or enamel absent) for internal nodes based on SIMMAP (Version 1.0 B2.3.2) [74]. Parsimony optimization (not shown) agreed with the most probable ML reconstruction. Functional branches lead to extant taxa and internal nodes with enamel. Pre-mutation, mixed, and pseudogene branches lead to extant taxa and internal nodes without enamel. Pre-mutation branches predate the first detected occurrence of a frameshift mutation or stop codon in *ENAM*. Mixed branches record the first detected occurrence of a frameshift or stop codon in *ENAM*. Pseudogene branches postdate the first detected occurrence of a frameshift or stop codon in *ENAM*. Branch colors are as in Figure 1.
(1.95 MB TIF)

Figure S4 Alternative hypotheses for the loss of enamel in Xenarthra given a basal position for the fossil armadillo *Utaetus* relative to living armadillos. Deltran parsimony optimization favors the dual loss hypothesis wherein enamel was lost independently in Pilosa and Dasypodidae; acctran parsimony optimization favors loss of enamel in the common ancestor of Xenarthra followed by gain of this feature in *Utaetus*. A dN/dS ratio of 0.48 suggests that *ENAM* evolved under purifying selection on the stem branch leading to crown Xenarthra and was a functional gene at this stage in its evolutionary history. Parsimony reconstruction of the partial *ENAM* sequence for the most recent common ancestor of Xenarthra also suggests that *ENAM* was functional (i.e., no frameshift mutations or stop codons in reconstructed ancestral sequence). Taken together, the dN/dS ratio for *ENAM* on the stem xenarthran branch and the reconstructed ancestral *ENAM* sequence in the last common ancestor of Xenarthra provide support for the dual loss hypothesis. Additional observations from genomics suggest that enamel may have been lost independently in more than one armadillo lineage (see text).
(0.74 MB TIF)

Figure S5 Representative chromatograms showing frameshift insertions and deletions in edentulous and enamelless taxa.
(0.29 MB PDF)

Figure S6 Pairwise amino acid alignments between *Sus* and *Dasypus* for the *AMELX, AMBN, ENAM,* and *MMP20* genes.
(0.04 MB DOC)

Figure S7 Primer sequences and location of primer sequences in relationship to nearly complete exonic sequences that were assembled from Ensembl 51 and trace files. F designates forward primers; R designates reverse primers.
(0.74 MB DOC)

Figure S8 Illustration of *ENAM* segments that amplified (black) and failed to amplify (white) for edentulous and enamelless taxa.
(0.87 MB TIF)

Table S1 Comprehensive list of frameshift insertions and deletions in edentulous and enamelless taxa. Numbering of insertions and deletions corresponds to the alignment in Dataset S1. Numbers in red font indicate the minimum to maximum number of frameshift indels.
(0.03 MB DOC)

Text S1 Supplementary note on enamel in (a) sperm whales and (b) xenarthrans.
(0.07 MB DOC)
