# Supplementary figures and images for: Molecular Decay of the Tooth Gene Enamelin (ENAM) Mirrors the Loss of Enamel in the Fossil Record of Placental Mammals
Source: PLoS Genet. 2009 Sep 4;5(9):e1000634. doi: 10.1371/journal.pgen.1000634 (PMC2728479; doi:10.1371/journal.pgen.1000634)

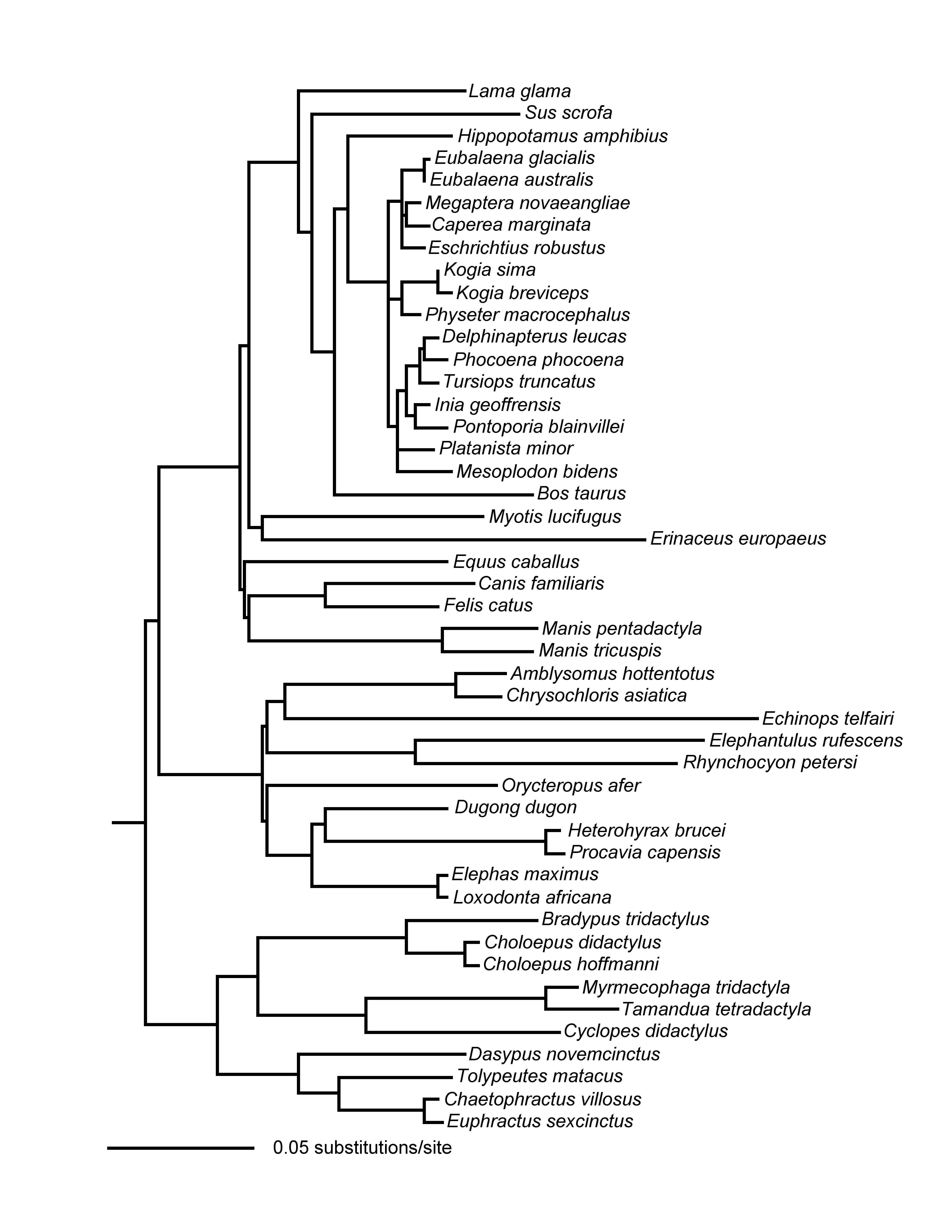

Supplement: Figure S1 — ML phylogram based on 4089 bp alignment (Dataset S1). Marsupial outgroups are not shown. (1.72 MB TIF) [file pgen.1000634.s003.tif]

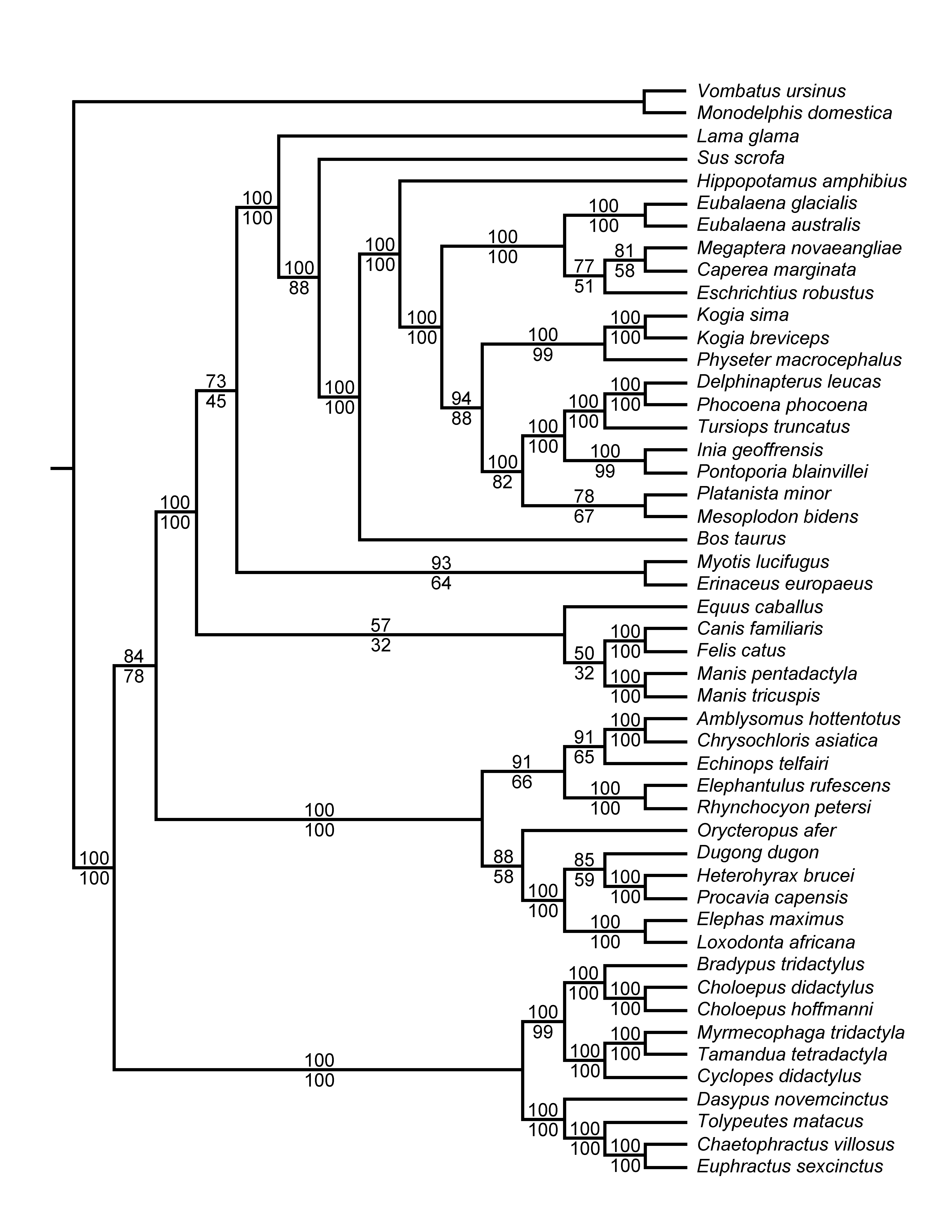

Supplement: Figure S2 — Maximum posterior probability tree with Bayesian posterior probabilities above branches and ML bootstrap support percentages below branches. Bayesian and ML analyses were performed with the 4089 bp alignment (Dataset S1). (1.81 MB TIF) [file pgen.1000634.s004.tif]

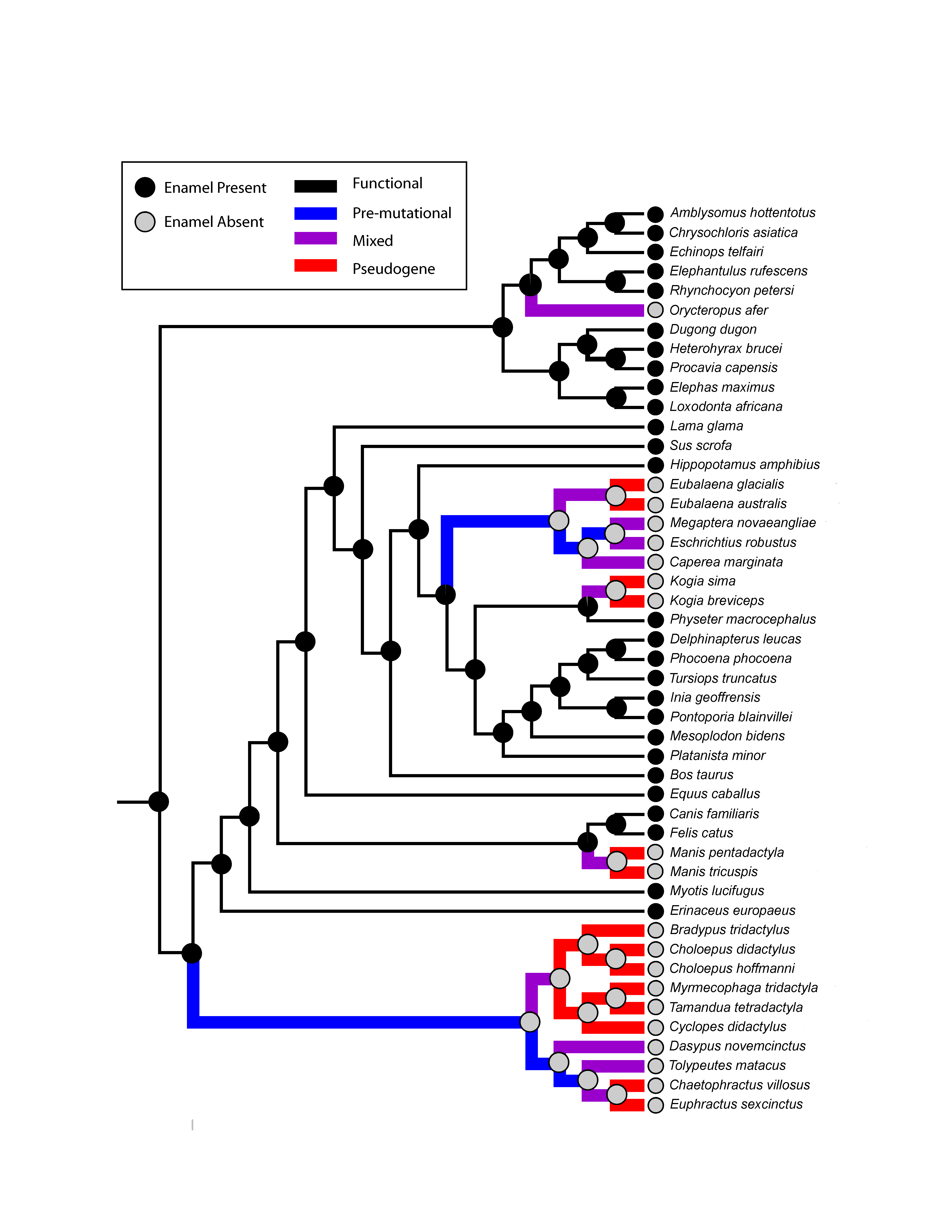

Supplement: Figure S3 — Ancestral state reconstructions (enamel present or enamel absent) for internal nodes based on SIMMAP (Version 1.0 B2.3.2) [74]. Parsimony optimization (not shown) agreed with the most probable ML reconstruction. Functional branches lead to extant taxa and internal nodes with enamel. Pre-mutation, mixed, and pseudogene branches lead to extant taxa and internal nodes without enamel. Pre-mutation branches predate the first detected occurrence of a frameshift mutation or stop codon in ENAM. Mixed branches record the first detected occurrence of a frameshift or stop codon in ENAM. Pseudogene branches postdate the first detected occurrence of a frameshift or stop codon in ENAM. Branch colors are as in Figure 1. (1.95 MB TIF) [file pgen.1000634.s005.tif]

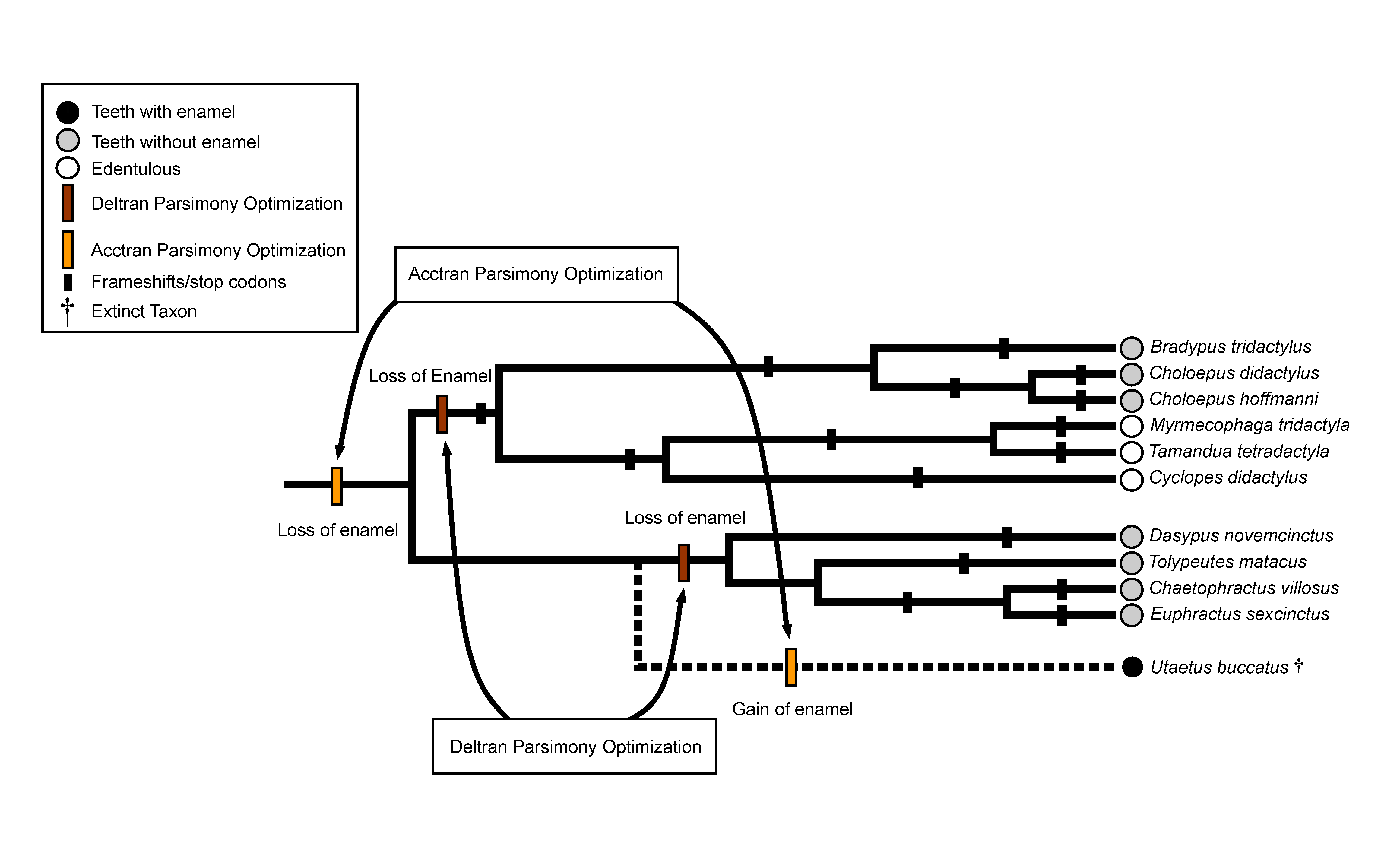

Supplement: Figure S4 — Alternative hypotheses for the loss of enamel in Xenarthra given a basal position for the fossil armadillo Utaetus relative to living armadillos. Deltran parsimony optimization favors the dual loss hypothesis wherein enamel was lost independently in Pilosa and Dasypodidae; acctran parsimony optimization favors loss of enamel in the common ancestor of Xenarthra followed by gain of this feature in Utaetus. A dN/dS ratio of 0.48 suggests that ENAM evolved under purifying selection on the stem branch leading to crown Xenarthra and was a functional gene at this stage in its evolutionary history. Parsimony reconstruction of the partial ENAM sequence for the most recent common ancestor of Xenarthra also suggests that ENAM was functional (i.e., no frameshift mutations or stop codons in reconstructed ancestral sequence). Taken together, the dN/dS ratio for ENAM on the stem xenarthran branch and the reconstructed ancestral ENAM sequence in the last common ancestor of Xenarthra provide support for the dual loss hypothesis. Additional observations from genomics suggest that enamel may have been lost independently in more than one armadillo lineage (see text). (0.74 MB TIF) [file pgen.1000634.s006.tif]

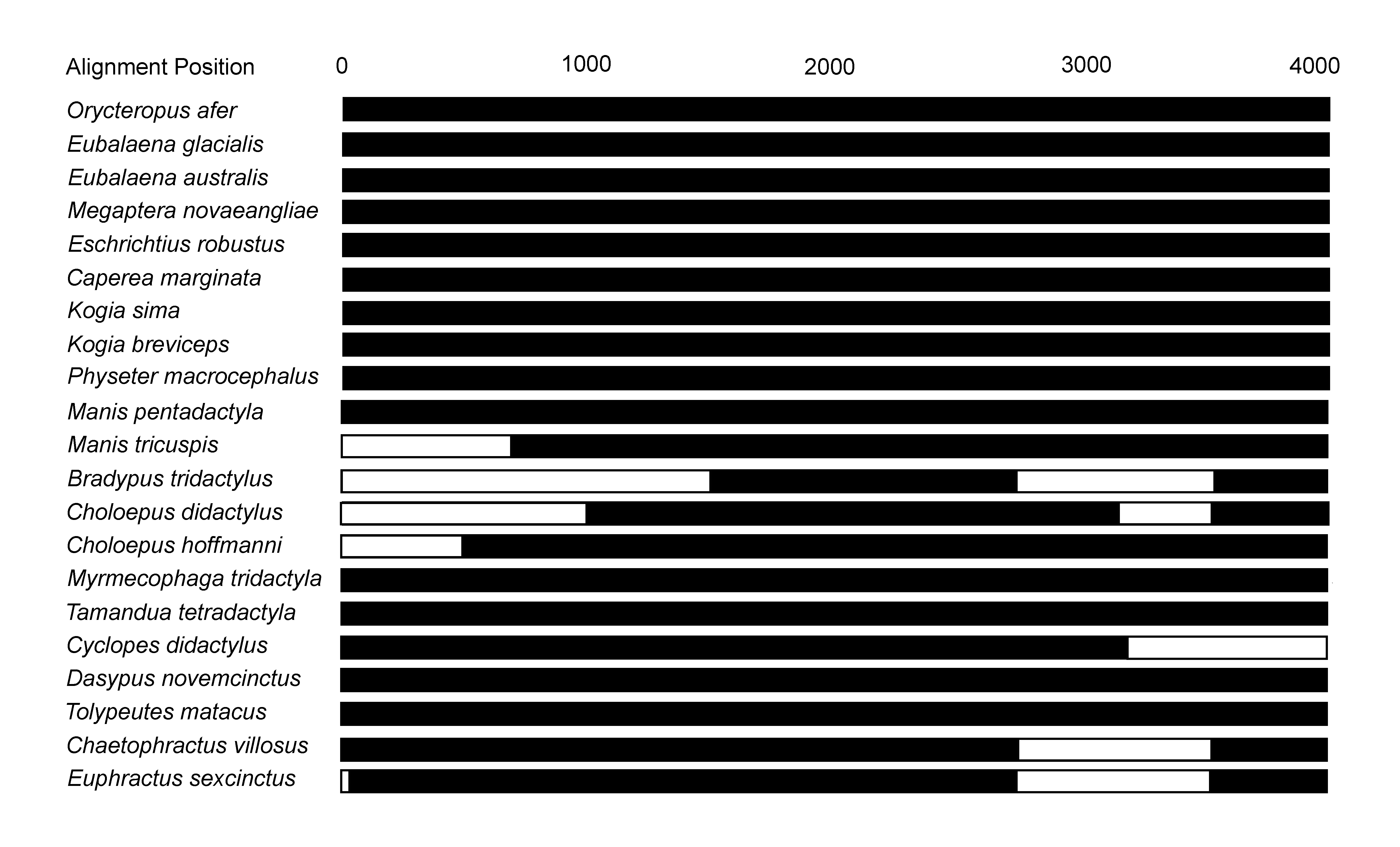

Supplement: Figure S8 — Illustration of ENAM segments that amplified (black) and failed to amplify (white) for edentulous and enamelless taxa. (0.87 MB TIF) [file pgen.1000634.s010.tif]
