# Supplementary material for: Molecular Decay of the Tooth Gene Enamelin (ENAM) Mirrors the Loss of Enamel in the Fossil Record of Placental Mammals
Source: PLoS Genet. 2009 Sep 4;5(9):e1000634. doi: 10.1371/journal.pgen.1000634 (PMC2728479; doi:10.1371/journal.pgen.1000634)
Supplement: Figure S5 — Representative chromatograms showing frameshift insertions and deletions in edentulous and enamelless taxa. (0.29 MB PDF) [file pgen.1000634.s007.pdf]

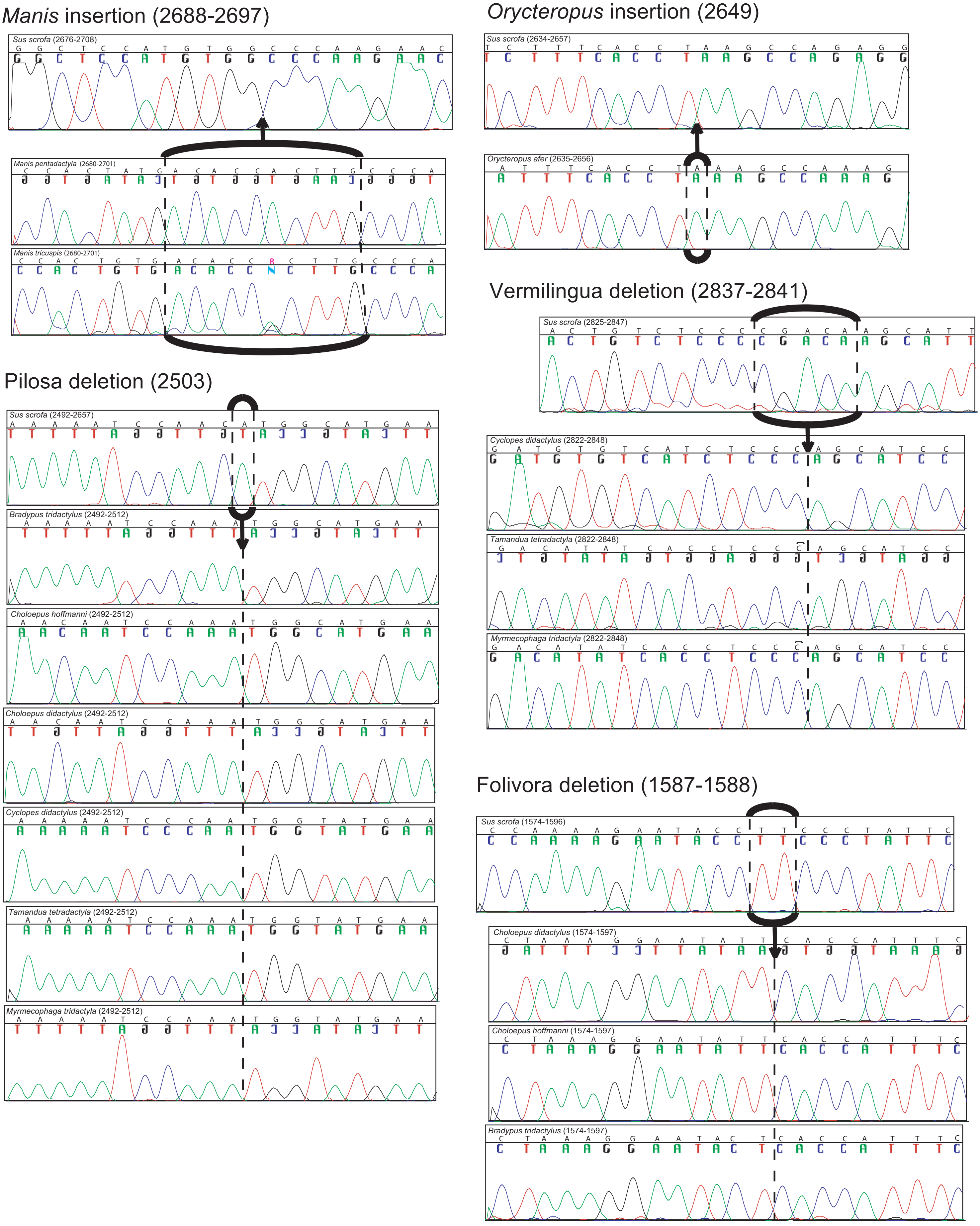

Manis insertion (2688-2697)
Sus scrofa (2676-2708)
Manis pentadactyla (2680-2701)
Manis tricuspis (2680-2701)
Orycteropus insertion (2649)
Sus scrofa (2634-2657)
Orycteropus afer (2635-2656)
Vermilingua deletion (2837-2841)
Sus scrofa (2825-2847)
Cyclopes didactylus (2822-2848)
Tamandua tetradactyla (2822-2848)
Myrmecophaga tridactyla (2822-2848)
Pilosa deletion (2503)
Sus scrofa (2492-2657)
Bradypus tridactylus (2492-2512)
Choloepus hoffmanni (2492-2512)
Choloepus didactylus (2492-2512)
Cyclopes didactylus (2492-2512)
Tamandua tetradactyla (2492-2512)
Myrmecophaga tridactyla (2492-2512)
Folivora deletion (1587-1588)
Sus scrofa (1574-1596)
Choloepus didactylus (1574-1597)
Choloepus hoffmanni (1574-1597)
Bradypus tridactylus (1574-1597)

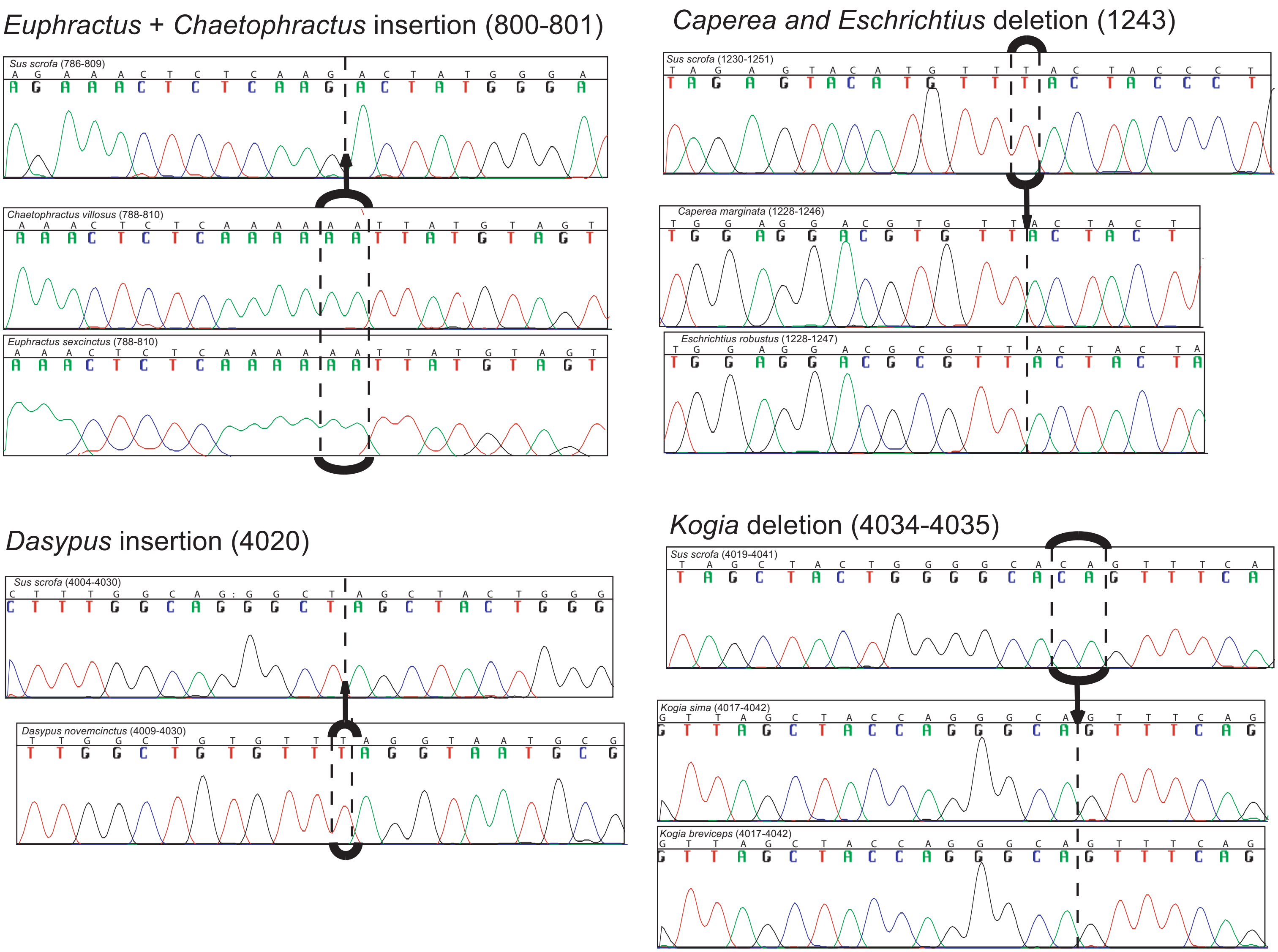
Euphractus + Chaetophractus insertion (800-801)
Sus scrofa (786-809)
Chaetophractus villosus (788-810)
Euphractus sexcinctus (788-810)
Caperea and Eschrichtius deletion (1243)
Sus scrofa (1230-1251)
Caperea marginata (1228-1246)
Eschrichtius robustus (1228-1247)
Dasypus insertion (4020)
Sus scrofa (4004-4030)
Dasypus novemcinctus (4009-4030)
Kogia deletion (4034-4035)
Sus scrofa (4019-4041)
Kogia sima (4017-4042)
Kogia breviceps (4017-4042)
