# Supplementary material for: Molecular Decay of the Tooth Gene Enamelin (ENAM) Mirrors the Loss of Enamel in the Fossil Record of Placental Mammals
Source: PLoS Genet. 2009 Sep 4;5(9):e1000634. doi: 10.1371/journal.pgen.1000634 (PMC2728479; doi:10.1371/journal.pgen.1000634)
Supplement: Figure S6 — Pairwise amino acid alignments between Sus and Dasypus for the AMELX, AMBN, ENAM, and MMP20 genes. (0.04 MB DOC) [file pgen.1000634.s008.doc]

**[Ameloblastin]**

*Sus* MPALKIPLFKMKDMVLILCLLKMSSAVPAFPRQPGTPGVASLSLETMRQL

*Dasypus* TSALKIPLFKMKVLILMLCLLGTSFAVPVFPQQPGTPGLASLSLETMRQL

*Sus* GSLQGLNMLSQYSRFGFGKSFNSLWMHGLLPPHSSFQWMRPREHETQQYE

*Dasypus* GSLQGLNMLPKFSRFGFGKSFNSLWIHDLLPPHSSFQWMHPREHETQQYE

*Sus* YSLPVHPPPLPSQPSLQPQQPGQKPFLQPTVVTSIQNPVQKGVPQPPIYQ

*Dasypus* YSLPVHPPPLPSQQSLQPQQPGQKPLPQPTVTTGTQDTAQKGGLHPPIHQ

*Sus* GHPPLQQVEGPMVQQQVAPSEKPPEAELPGLDFADPQDPSMFPIARLISQ

*Dasypus* GQPPLQQGEMPAIQQQVAPSEKPPKPELPGLEFAAPQGRSIFPSSRLISS

*Sus* GPVPQDKPSPLYPGMFYMSYGANQLNSPARLGILSSEEMAGGRGGPLAYG

*Dasypus* GPMPQTKQPQLYPGMFYMSYGTNQLNAPARLGIMSSEEMAGGRGGPMAYG

*Sus* AMFPGFGGMRPNLGGMPPNSAKGGDFTLEFDSPAAGTKGPEKGEGGAEGS

*Dasypus* AMFPGFGGMRPGFGGMPPNPAMGGDFTLEFDSTVSGTKGPEKGEGGAQGS

*Sus* PVAEANTADPESPALFSEVASGVLGGLLANPKGKIPNLARGPAGRSRGPP

*Dasypus* PVPDGNPANTENPALPAEGAPAAHGGLLVFPKGNILSLAKGPAGQSRGPP

*Sus* GVTPADADPLMTPGLADAYETYGADETTTLGLQEEMTMDSTATPYSEHTS

*Dasypus* RVTPAAADPLMTPGLADAYGTYIAGVTTPLDVPEESTTDTTMTPDTEQTS

*Sus* MPGNKAQQPQIKRDAWRFQEP

*Dasypus* MPGNKVQQPGVMHHMWHFQEP

**[Amelogenin]**

*Sus* MPLPPHPGHPGYINFSYEVLTPLKWYQNMIRHPYTSYGYEPMGGWLHHQI

*Dasypus* MPLPPHPGHPGYINFSYEVLSPLKWYQS-IRQPYPSYGYEPMSGWLHQQI

*Sus* IPVVSQQTPQSHALQPHHHIPMVPAQQPGIPQQPMMPLPGQHSMTPTQHH

*Dasypus* IPVLSQQHPPSHALQPHQHIPMMPAQEPMVPQQPMMPVPGQHSMTPSLHH

*Sus* QPNLPLPAQQPFQPQPVQPQPHQPLQPQSPMHPIQPLLPQPPLPPMFSMQ

*Dasypus* QPNLPLPVQQPFQPQPAQPQPHQPIQPQPPMQPIQP-----PLPPVFAMQ

*Sus* SLLP----DLPLEAWPATDKTKREEVD

*Dasypus* PLAPMLPGDLPLEAWPATDKTKREEVV

**[Enamelysin (MMP-20)]**

*Sus* MKVLPASGLAVLLVTALKFSAAAPSLFAATPRTSRNNYHLAQAYLDKYYT

*Dasypus* MAVLPAPGLAVL-VTAWAFSTAAPSLLAAT-----NSYHLAQAYLDKYYT

*Sus* KKGGHQVGEMVAKGGNSMVKKIKELQAFFGLRVTGKLDRTTMDVIKRPRC

*Dasypus* KKGGQQIGEMVSRGGSSMVKKLKELQAFFGLHVTGKLDQPTLEMIQKPRC

*Sus* GVPDVANYRLFPGEPKWKKNTLTYRISKYTPSMTPAEVDKAMEMALQAWS

*Dasypus* GVPDVANYRLFPGEPKWKKNTLTYRISKYTPSLSAAAVDAAVEMALCAWS

*Sus* SAVPLSFVRVNAGEADIMISFETGDHGDSYPFDGPRGTLAHAFAPGEGLG

*Dasypus* SAVPLTFVRTDVGEADIMVSFETGDHGDSYPFDGPRGTLAHAFAPGEGLG

*Sus* GDTHFDNAEKWTMGMNGFNLFTVAAHEFGHALGLAHSTDPSALMYPTYKY

*Dasypus* GDTHFDNAETWTLGMNGFNLFTVAAHEFGHALGLAHSTDPSALMYPTYKY

*Sus* QNPYGFHLPKDDVKGIQALYGPRKTFTGKPTVPHGPPHNPSLPDICDSSS

*Dasypus* QSPYGFQLPKDDVRGIQALYGPRRTFLGKPTVPHIPPHGPSTPEHCDSST

*Sus* SFDAVTMLGKELLFFRDRIFWRRQVHLMSGIRPSTITSSFPQLMSNVDAA

*Dasypus* SFDAVTMLGKELLFFRDRIFWRRQVHQPLGVRPSTTTSSFPQLMANVDAA

*Sus* YEVADRGMAYFFKGPHYWITRGFQMQGPPRTIYDFGFPRYVQRIDAAVHL

*Dasypus* YQVAERGTAYFFKGPHYWVTRGFQMQGPPQTIYDFGFPRSVQRIDAALHL

*Sus* KDTQKTLFFVGDEYYSYDERKRKMDKDYPKNTEEEFSGVNGQIDAAVELN

*Dasypus* QEAQRTLFFVGDEHYSYDEKKRKMEKDYPKNTEEEFSGVNGQIDAAVELN

*Sus* GYIYFFSGPKAYKYDTEKEDVVSVLKSNSWIGC

*Dasypus* GYIYFFSGPKSFKYDTEKEDVVGVVKSSSWIGC

**[Enamelin]**

*Sus* MPMQMPRMPGFSSKSEEMMRYGHFNFMNAPHMAHLGTLYGNGMQLPQFFP

*Dasypus* MP-HMPRMPGFSSKSEEMIWYGQFNFVNSPHMGHLGPVYGNGIQFPQLFP

*Sus* QYQMPMWPQPPPNKKHPQKPSAS---KQQSKTDPAPESQKPNQPQPKTPT

*Dasypus* QYQMPMWPHPPPNTWHPQKPSPPSAPKHHSKTGQAQGTQKTNQPESKKPP

*Sus* PKQPLNEPSPTPTQPEEETQTPQAFPPFGNGLFPYQQPLWHVPHRIPPGY

*Dasypus* PKRPLKQPLHAPTQPQEEPSPPQEFPPFANGLFPYQQPPWQMPQRIPPGY

*Sus* GRPPTSNEEGGNPYFGFFGYHGFGGRPPYYSEEMFEQDFEKPKEKDPPKT

*Dasypus* GRPPISNEEGGNPYFGYFGYHGFGGRPPYYSEEMFEQNFEKPKEKDPPKT

*Sus* ETPATEPSVNTTVPETNSTQPNAPNPR----GNDTSPTGTSGQGPNPRSN

*Dasypus* ESPAAEPTTNSTVPATNSSQPNTPGTGGSQGGNGTSPTGNSAHVPNPGNN

*Sus* PTGQN----GPAVNVSGQGVPRSQSPWGPRQTIIHENYPNPNIRGFPARR

*Dasypus* PTAQNVATPSPPIYVSGQEMPRSQIPWRPSQSNIHEKYPNPNIRDFPARR

*Sus* QWRPPGPAMGHRRNGPFYRNQQIQRGPRWNSFTLEGKQAVRPGYPTYRRV

*Dasypus* PWHPSGNVMGHRENGPFYRNQ-VQRGPRWNSFALESKKAVLLGNPAYHKV

*Sus* YGSTARSNPPNYAGNSANLRRKPEGPNKNPMVTNVAPPGPKHGTVDQNEN

*Dasypus* CPSISRGSLPKHAGNPANFRRKPQGLNKHTVGTNVALLGPSHGTVGHSEK

*Sus* IQNPREKQVSQKERTVVPTRDPSGPWRNSQDYGINKSNYKLPQPEDNMLV

*Dasypus* FQNPRENSVSQKETIGSPTRDPTDPWRNSHDNGANKSNYKLPDSEGNRPA

*Sus* PNFNSIDQRENSYYPRGESKRAPNSDGQTQTQIIPKGIVLEPRRIPYESE

*Dasypus* PSFNSIDQQENSYYPRGDSRRTSNSDGQTQSLHLPKGIVLEPRRIPHESE

*Sus* TNQPELKHSAYQPVYTEGIPSPAKEHFPAGRNTWNQQEISPPFKEDPGRQ

*Dasypus* TSWPELKHSTFHPVYPEEIPSSARENFPAGRNIWNHQEISPNFGKDPARQ

*Sus* EEH---------LPHLSHGSRVHVYYPDYNPYDPRENSPYLRSNTWYERD

*Dasypus* EGHSPPGRQDGHLPQSSHGSRGSVFYPEYNPYDPRENSPYLRGNTWDE-D

*Sus* DSPNTMGQPENPHYPMNTPDPKETIPYNEEDPIDPTGDEHFPGQSRWDME

*Dasypus* DSPNTMGQPEHPLYPMNTPDQKKRIPYNEEDPADPTGDETFPGQSRWGEE

*Sus* ELSFKEDPTVRHYEGEQYTSNQPKEYLPYSLDNPSKPREDFLYGEFYPWN

*Dasypus* ESNFKGDPTIRHYEGKQYTPNQPKEYLHHSLDNPSKPREDFPYGEFYPWG

*Sus* PEENFPSYNTAPTVSSPVESRGYYANNAVGQEESTMFPSWSSWDPRIQAQ

*Dasypus* PDENFPSYNTASTVPPPVESRGYYVNNAVGQEESTLFPSWNTWYNRIQAH

*Sus* GQKEGRPYLNRNFWDQSTNLYKTPTSSPHQKENQPYSNNSPAGLQKNPTW

*Dasypus* GQKEGRPYVKENFWDEVPNLQKAPAISPDWKKNQHYFSNYPNRLQKNPKW

*Sus* HEGENLNYGMQITRLNSPERDHLAFPDLIPPDYPGGQKESHVFHLSQRGP

*Dasypus* HEDGNLNYGMQITRLNSPEGKHLAFPELIPQNYPTGQDEANLFHLSQRSS

*Sus* CCAGGSMWPKNNPLALQDYTQSFGLAPGENPDTSIGYAEDSHIKYARQTV

*Dasypus* CCA--VTRPKDNPLALQEYTPSFSLVPGENKDTSPLNTEGTHTKHARHII

*Sus* SPTSIVPGQRNSSEKILPGESQNPSPFKDDVSTLRRSTPCSVKSQLSQRG

*Dasypus* SLVSTLSSQTNSSEKRLPGESQNPSPFRDYVSTLRRNTPYSINNQQGQRE

*Sus* IMPLPEANSLQSKNTPCLTSDLGGDGNNVLEQIFEGNQLNERTVDLTPEQ

*Dasypus* IMPLLEASSLQSKNTHCLKIGLGGDGNNVLGQIFEGNPINQRTAELTPEQ

*Sus* LVFGTPDKEPRPEGIPNEMQGNESERQQQRQSSILQLPCFGSKLANYHTS

*Dasypus* LVISTPDKGPKPEGIQSKVQRNEGERQKQIPSSIQQLPCFGSKVAKPHSS

*Sus* SIGTPSSLGRQDSFDGDPIMPTETPNSLAGLATGAQFQNINVDPLNEDEH

*Dasypus* GTVTPSSTGRRGLFDGDPIKPTETPNMLAVFR*CGTV*EWKCKPT*CR*T

[“T” insert in DNA]

*Sus* TPFDSLQIGTNPQDQVQDCLLLQA

*Dasypus* PSI*IL*KRDQSTRPGTRLLTTSG
