# Supplementary material for: Molecular Decay of the Tooth Gene Enamelin (ENAM) Mirrors the Loss of Enamel in the Fossil Record of Placental Mammals
Source: PLoS Genet. 2009 Sep 4;5(9):e1000634. doi: 10.1371/journal.pgen.1000634 (PMC2728479; doi:10.1371/journal.pgen.1000634)
Supplement: Figure S7 — Primer sequences and location of primer sequences in relationship to nearly complete exonic sequences that were assembled from Ensembl 51 and trace files. F designates forward primers; R designates reverse primers. (0.74 MB DOC) [file pgen.1000634.s009.doc]

Primer sequences and location of primer sequences in relationship to nearly complete exonic sequences that were assembled from Ensembl 51 and trace files. F designates forward primers; R designates reverse primers.

**Afrotheria and Xenarthra Primers**

>Procavia

AATCCTTACTTTGGATATTTTGGATATCATGGATTTGGGGGTCGCCCTCCTTATTATTCAGAAGAGATGTTTGAACAAGA

TTTTGAAAAACCCAAAGAAAAAGATCCCCCTAAAACAGAGAGTCCAGCTACAGACCCCACACCTAATACAACTGTTCCTG

AGACTAATTCAACCCAATCAAATGTGCCTGGCTCTGGCGGGAATCATGGAGGAAATGACACCACCCTAACAGGAAACAGT

CATGGCCCAAACTTGGGGAGCAACCCTACAGCTCAAAATGGGGTCATTTCTCCCTCTACAGCTAATGTTTCAAACCAGGG

AGTACCACGAAGTCAAAATCCATGGGGACCAAGTTACCCAAATATTCAYGAAAATTATCCATATCCTGGTGCCCAGGGTT

CTCCTATAGCAAGACAATGGCGTCCCATTGGTACTGCCATGGGCCACAGACAGAATGGGCCTCTTTACCGAAATCAACAG

GTTCAAAGGGGTCCTCGGTGGAACTCCTTTACTTTGGAAGGCAAACCAGCAGCTCGTCCAGGAAATCCAGCTTATCAAAA

AGYTTACCCTTCCATTTCAAGAGGTAATTCTCTCAATTATGCAAGAAATCCAGCAAATTTCAGAAGAAAGCCTCAGTTGC

CAAATAAATACCCTACAGGAGCCAATGCTGCCCCACTGGGAACTAAACAGGGTACTGTTGGACGCAATGAAAAAATCCRA

AGTCCAGGAGAGAAGTCACCAGCTCAAAAAGAAAGAATAGTTGTTCCTACAAGGGAACCAACTGGCCTCAGRAAAAACTC

TCAAGATTTTGGAGTTAGTAAACCCAATTATAAACTGCCTGGTTCTGAGGGCAACATGCAAGTTCCAAGTTTTAATACCA

TTGATCAACACGAAAACTCCTACTACCCAAGAGAAGATTCGAGGAGAGTCCAAAATTCTGATGGACAAACCCCAAACCAG

TATTTGCCCAAAGGGGTTGTTTTAGAACCAAGAAGAATCCCATATGAATCAGAAACTAATGGGCCAGAGTTAAAACATAG

TACATATCATCCTGTATACCCTGAGGAAATTCCTTCCCCTGCAAGAGAACATTTTCCTGCTGAAAGAAAACACTGGAATC

AGCAAATCTCTCCAACTCTTGAGGAAGATCCTGGAAAGCAGGTTAGACCTTTGCCTTATCCTTCCCATGGCACTAGAGGA

AGTGTTTTCTACCCTGAATATAACTCCTATGATCCCAGGGAAAATTCATACCTTAGAAGTAATACCTGGGATGAAAGAGA

TAATTCTCCCAATACCATTAGACGACCAGAAACTTCACTATACCCCATGAACACACCAGACCAGAAAGAGACAGTCCCTT

ACAATGAGGAGGACCCAACTGATCCAACAGGAGATGAAACTTTCCTAGAACCAGGTAGATGGGGTAAGGAAGAATCCAGC

TTTAAAGAAAGTCCCCAAATTAGGCAGTACGAAGGCCAACGATATGCCTCCAATCAGCCAAAAGAATACCTCCCTTATTC

CTTAGACAATCCTTCGAAACCCAGAGAGGATTTTCCATATGGTGAATTTTACCCCTGGGGCCCTGATGAGAATTTTCCGT

CATATAATACAGCTCCTACTCTACCACCACCAGTGGAGGGCCGGGGCTATTACATTAATAACGCTGTTGAACGAGAAGTA

GGTCCTCTGTTTCCTTCTTGGAACTCCTGGGACCACAGGATTCAAGCTCAGGGACCAGAAGAAAAAGGGCCATATTTTAG

TAGAAATTTTTGGGATCAGACAACGAATTTACACAAAGCTCCAGCTGTGTTACCAGACCATAATGAGATCCAGCCCTATT

CCTCTAACTCCCCAGTTGGGCTTCAGAAAAATCCAACCTGGCATGACAGCGAGAATTTGAACTACGGCATGCAAATTACT

AGGTTAAATTCACCAGAGGGGGAACGTTTGGCTTTCCCAGACTTGATTCCTCCAAGTTACTCAATTGATCAAAAAGAAAC

AAATTTGTTTCACCCAAGACAAAGAGGCCCCTGCTGTGCTGGTGGCTCCACAGGGCCCAAGGACAATCTGCTTGCTCTAC

AAGACTACACTCCATCCTTGGGCCTTGCGCCAGGGGAGAACCAAGACACCAGCCCTCTATATACAGAAAGCAGTCATACT

AAGCATGCAAGACATAGTATCTCCCCTACAAGCATCCTACCTGACCAAAGAAACAGCTCAGAGAAGAGAATGCCTGGGGA

AAGCCAAAACTCAAGTCCCTTCAGAGACGATGTGTCCACTCTGAGGAGGAACACACCATGTTCTGTGAAGAGTCAACTGG

GTCAAATGGGAATGATGCCTCTTCCTGAAACTAGCTCCCTTCAGTTGAACACACCGTGTCTCAAAAGTGATCATGGTGGA

GATGAGAACAACGTTATGGAACAAATGTTTGAAGATAACCAACCCAGTGAAAGAACTGTTGACCTTACCCCTGAGCAGCT

TGTTATAGGTACCCCTGATGAAGATCCTAAACCAGAAGCAATGCAAAGTGAACTCCTAGGACACGACAGTGAGAGGCAAC

AACAAAGGCCACCTAGCATCCTGCAGTTACCTTGCTTTGGCAAATTAGCAAAGCATCACTTCTCTGGCACTGGAACTCCA

TCCAGCAATGGAAGGCAAGGCTCATTTGATGGGGATCCAATCATGCCAACTGAAAATCCTAACACTTTTGCTGAGTTAGC

TACAGAGGCACAGTTTAACGGTGTCAATGTAAACACTCTTAATGCAGGTGAACACCCTCCATTTGACTCCAATCCACAGG

ACCAGGTACAAGACTGCTTATTACTTCAGGCTTAGGGA--------------------

>Enam-AfroXenScanF1

---CCTTACTTTGGATATTTTGGATATCATGG------------------------------------------------

--------------------------------------------------------------------------------

--------------------------------------------------------------------------------

--------------------------------------------------------------------------------

--------------------------------------------------------------------------------

--------------------------------------------------------------------------------

--------------------------------------------------------------------------------

--------------------------------------------------------------------------------

--------------------------------------------------------------------------------

--------------------------------------------------------------------------------

--------------------------------------------------------------------------------

--------------------------------------------------------------------------------

--------------------------------------------------------------------------------

--------------------------------------------------------------------------------

--------------------------------------------------------------------------------

--------------------------------------------------------------------------------

--------------------------------------------------------------------------------

--------------------------------------------------------------------------------

--------------------------------------------------------------------------------

--------------------------------------------------------------------------------

--------------------------------------------------------------------------------

--------------------------------------------------------------------------------

--------------------------------------------------------------------------------

--------------------------------------------------------------------------------

--------------------------------------------------------------------------------

--------------------------------------------------------------------------------

--------------------------------------------------------------------------------

--------------------------------------------------------------------------------

--------------------------------------------------------------------------------

--------------------------------------------------------------------------------

--------------------------------------------------------------------------------

--------------------------------------------------------------------------------

--------------------------------------------------------------------------------

--------------------------------------------------------------------------------

--------------------------------------------------------------------------------

----------------------------------------------------------

>Enam-AfroXenScanF2

-------------GATATTTTGGATATCATGGMTTTGGGGG---------------------------------------

--------------------------------------------------------------------------------

--------------------------------------------------------------------------------

--------------------------------------------------------------------------------

--------------------------------------------------------------------------------

--------------------------------------------------------------------------------

--------------------------------------------------------------------------------

--------------------------------------------------------------------------------

--------------------------------------------------------------------------------

--------------------------------------------------------------------------------

--------------------------------------------------------------------------------

--------------------------------------------------------------------------------

--------------------------------------------------------------------------------

--------------------------------------------------------------------------------

--------------------------------------------------------------------------------

--------------------------------------------------------------------------------

--------------------------------------------------------------------------------

--------------------------------------------------------------------------------

--------------------------------------------------------------------------------

--------------------------------------------------------------------------------

--------------------------------------------------------------------------------

--------------------------------------------------------------------------------

--------------------------------------------------------------------------------

--------------------------------------------------------------------------------

--------------------------------------------------------------------------------

--------------------------------------------------------------------------------

--------------------------------------------------------------------------------

--------------------------------------------------------------------------------

--------------------------------------------------------------------------------

--------------------------------------------------------------------------------

--------------------------------------------------------------------------------

--------------------------------------------------------------------------------

--------------------------------------------------------------------------------

--------------------------------------------------------------------------------

--------------------------------------------------------------------------------

----------------------------------------------------------

>Enam-AfroXenIF1

--------------------------------------------------------------------------------

--------------------------------------------------------------------------------

--------------------------------------------------------------------------------

--------------------------------------------------------------------------------

--------------------------------------------------------------------------------

--------------------------------------------CACAGASAGAATGGRCCTYTTTAYCGAAATC-----

--------------------------------------------------------------------------------

--------------------------------------------------------------------------------

--------------------------------------------------------------------------------

--------------------------------------------------------------------------------

--------------------------------------------------------------------------------

--------------------------------------------------------------------------------

--------------------------------------------------------------------------------

--------------------------------------------------------------------------------

--------------------------------------------------------------------------------

--------------------------------------------------------------------------------

--------------------------------------------------------------------------------

--------------------------------------------------------------------------------

--------------------------------------------------------------------------------

--------------------------------------------------------------------------------

--------------------------------------------------------------------------------

--------------------------------------------------------------------------------

--------------------------------------------------------------------------------

--------------------------------------------------------------------------------

--------------------------------------------------------------------------------

--------------------------------------------------------------------------------

--------------------------------------------------------------------------------

--------------------------------------------------------------------------------

--------------------------------------------------------------------------------

--------------------------------------------------------------------------------

--------------------------------------------------------------------------------

--------------------------------------------------------------------------------

--------------------------------------------------------------------------------

--------------------------------------------------------------------------------

--------------------------------------------------------------------------------

----------------------------------------------------------

>Enam-AfroXenIR1

--------------------------------------------------------------------------------

--------------------------------------------------------------------------------

--------------------------------------------------------------------------------

--------------------------------------------------------------------------------

--------------------------------------------------------------------------------

--------------------------------------------------------------------------------

--------------------------------------------------------------------------------

----------------------------------------GCARGAAAYCCAGCAAATTTCAGAAGAAAGCC--------

--------------------------------------------------------------------------------

--------------------------------------------------------------------------------

--------------------------------------------------------------------------------

--------------------------------------------------------------------------------

--------------------------------------------------------------------------------

--------------------------------------------------------------------------------

--------------------------------------------------------------------------------

--------------------------------------------------------------------------------

--------------------------------------------------------------------------------

--------------------------------------------------------------------------------

--------------------------------------------------------------------------------

--------------------------------------------------------------------------------

--------------------------------------------------------------------------------

--------------------------------------------------------------------------------

--------------------------------------------------------------------------------

--------------------------------------------------------------------------------

--------------------------------------------------------------------------------

--------------------------------------------------------------------------------

--------------------------------------------------------------------------------

--------------------------------------------------------------------------------

--------------------------------------------------------------------------------

--------------------------------------------------------------------------------

--------------------------------------------------------------------------------

--------------------------------------------------------------------------------

--------------------------------------------------------------------------------

--------------------------------------------------------------------------------

--------------------------------------------------------------------------------

----------------------------------------------------------

>Enam-AfroXenScanR1

--------------------------------------------------------------------------------

--------------------------------------------------------------------------------

--------------------------------------------------------------------------------

--------------------------------------------------------------------------------

--------------------------------------------------------------------------------

--------------------------------------------------------------------------------

--------------------------------------------------------------------------------

--------------------------------------------------------------------------------

--------------------------------------------------------------------------------

--------------------------------------------------------------------------------

--------------------------------------------------------------------------------

---------------------------------------------------------CTGATGGACAAACYCAAAACWAG

HATTTG--------------------------------------------------------------------------

--------------------------------------------------------------------------------

--------------------------------------------------------------------------------

--------------------------------------------------------------------------------

--------------------------------------------------------------------------------

--------------------------------------------------------------------------------

--------------------------------------------------------------------------------

--------------------------------------------------------------------------------

--------------------------------------------------------------------------------

--------------------------------------------------------------------------------

--------------------------------------------------------------------------------

--------------------------------------------------------------------------------

--------------------------------------------------------------------------------

--------------------------------------------------------------------------------

--------------------------------------------------------------------------------

--------------------------------------------------------------------------------

--------------------------------------------------------------------------------

--------------------------------------------------------------------------------

--------------------------------------------------------------------------------

--------------------------------------------------------------------------------

--------------------------------------------------------------------------------

--------------------------------------------------------------------------------

--------------------------------------------------------------------------------

----------------------------------------------------------

>Enam-AfroXenScanR2

--------------------------------------------------------------------------------

--------------------------------------------------------------------------------

--------------------------------------------------------------------------------

--------------------------------------------------------------------------------

--------------------------------------------------------------------------------

--------------------------------------------------------------------------------

--------------------------------------------------------------------------------

--------------------------------------------------------------------------------

--------------------------------------------------------------------------------

--------------------------------------------------------------------------------

--------------------------------------------------------------------------------

-----------------------------------------------------------------CAAACYCAAAACWAG

HATTTGCCCAAAGG------------------------------------------------------------------

--------------------------------------------------------------------------------

--------------------------------------------------------------------------------

--------------------------------------------------------------------------------

--------------------------------------------------------------------------------

--------------------------------------------------------------------------------

--------------------------------------------------------------------------------

--------------------------------------------------------------------------------

--------------------------------------------------------------------------------

--------------------------------------------------------------------------------

--------------------------------------------------------------------------------

--------------------------------------------------------------------------------

--------------------------------------------------------------------------------

--------------------------------------------------------------------------------

--------------------------------------------------------------------------------

--------------------------------------------------------------------------------

--------------------------------------------------------------------------------

--------------------------------------------------------------------------------

--------------------------------------------------------------------------------

--------------------------------------------------------------------------------

--------------------------------------------------------------------------------

--------------------------------------------------------------------------------

--------------------------------------------------------------------------------

----------------------------------------------------------

>Enam-AfroXenF3

--------------------------------------------------------------------------------

--------------------------------------------------------------------------------

--------------------------------------------------------------------------------

--------------------------------------------------------------------------------

--------------------------------------------------------------------------------

--------------------------------------------------------------------------------

--------------------------------------------------------------------------------

--------------------------------------------------------------------------------

--------------------------------------------------------------------------------

---------------------------------------------CCTACAAGGGAWCCAACTGRCCCTWGG--------

--------------------------------------------------------------------------------

--------------------------------------------------------------------------------

--------------------------------------------------------------------------------

--------------------------------------------------------------------------------

--------------------------------------------------------------------------------

--------------------------------------------------------------------------------

--------------------------------------------------------------------------------

--------------------------------------------------------------------------------

--------------------------------------------------------------------------------

--------------------------------------------------------------------------------

--------------------------------------------------------------------------------

--------------------------------------------------------------------------------

--------------------------------------------------------------------------------

--------------------------------------------------------------------------------

--------------------------------------------------------------------------------

--------------------------------------------------------------------------------

--------------------------------------------------------------------------------

--------------------------------------------------------------------------------

--------------------------------------------------------------------------------

--------------------------------------------------------------------------------

--------------------------------------------------------------------------------

--------------------------------------------------------------------------------

--------------------------------------------------------------------------------

--------------------------------------------------------------------------------

--------------------------------------------------------------------------------

----------------------------------------------------------

>Enam-AfroXenF4

--------------------------------------------------------------------------------

--------------------------------------------------------------------------------

--------------------------------------------------------------------------------

--------------------------------------------------------------------------------

--------------------------------------------------------------------------------

--------------------------------------------------------------------------------

--------------------------------------------------------------------------------

--------------------------------------------------------------------------------

--------------------------------------------------------------------------------

---------------------------------------------------------CCAACTGRCCCTWGGAGAAACTC

TCAWG---------------------------------------------------------------------------

--------------------------------------------------------------------------------

--------------------------------------------------------------------------------

--------------------------------------------------------------------------------

--------------------------------------------------------------------------------

--------------------------------------------------------------------------------

--------------------------------------------------------------------------------

--------------------------------------------------------------------------------

--------------------------------------------------------------------------------

--------------------------------------------------------------------------------

--------------------------------------------------------------------------------

--------------------------------------------------------------------------------

--------------------------------------------------------------------------------

--------------------------------------------------------------------------------

--------------------------------------------------------------------------------

--------------------------------------------------------------------------------

--------------------------------------------------------------------------------

--------------------------------------------------------------------------------

--------------------------------------------------------------------------------

--------------------------------------------------------------------------------

--------------------------------------------------------------------------------

--------------------------------------------------------------------------------

--------------------------------------------------------------------------------

--------------------------------------------------------------------------------

--------------------------------------------------------------------------------

----------------------------------------------------------

>Enam-AfroXenIF2

--------------------------------------------------------------------------------

--------------------------------------------------------------------------------

--------------------------------------------------------------------------------

--------------------------------------------------------------------------------

--------------------------------------------------------------------------------

--------------------------------------------------------------------------------

--------------------------------------------------------------------------------

--------------------------------------------------------------------------------

--------------------------------------------------------------------------------

--------------------------------------------------------------------------------

--------------------------------------------------------------------------------

--------------------------------------------------------------------------------

--------------------------------------------------------------------------------

------------------------GGAAATYCCTTCCYCTGCAAGAGAAMATTTTCC-----------------------

--------------------------------------------------------------------------------

--------------------------------------------------------------------------------

--------------------------------------------------------------------------------

--------------------------------------------------------------------------------

--------------------------------------------------------------------------------

--------------------------------------------------------------------------------

--------------------------------------------------------------------------------

--------------------------------------------------------------------------------

--------------------------------------------------------------------------------

--------------------------------------------------------------------------------

--------------------------------------------------------------------------------

--------------------------------------------------------------------------------

--------------------------------------------------------------------------------

--------------------------------------------------------------------------------

--------------------------------------------------------------------------------

--------------------------------------------------------------------------------

--------------------------------------------------------------------------------

--------------------------------------------------------------------------------

--------------------------------------------------------------------------------

--------------------------------------------------------------------------------

--------------------------------------------------------------------------------

----------------------------------------------------------

>Enam-AfroXenIR2

--------------------------------------------------------------------------------

--------------------------------------------------------------------------------

--------------------------------------------------------------------------------

--------------------------------------------------------------------------------

--------------------------------------------------------------------------------

--------------------------------------------------------------------------------

--------------------------------------------------------------------------------

--------------------------------------------------------------------------------

--------------------------------------------------------------------------------

--------------------------------------------------------------------------------

--------------------------------------------------------------------------------

--------------------------------------------------------------------------------

--------------------------------------------------------------------------------

--------------------------------------------------------------------------------

--------------------------------------------------------------------------------

--------CTACYCTGAATATAACYCCTATGATCCCAGGG----------------------------------------

--------------------------------------------------------------------------------

--------------------------------------------------------------------------------

--------------------------------------------------------------------------------

--------------------------------------------------------------------------------

--------------------------------------------------------------------------------

--------------------------------------------------------------------------------

--------------------------------------------------------------------------------

--------------------------------------------------------------------------------

--------------------------------------------------------------------------------

--------------------------------------------------------------------------------

--------------------------------------------------------------------------------

--------------------------------------------------------------------------------

--------------------------------------------------------------------------------

--------------------------------------------------------------------------------

--------------------------------------------------------------------------------

--------------------------------------------------------------------------------

--------------------------------------------------------------------------------

--------------------------------------------------------------------------------

--------------------------------------------------------------------------------

----------------------------------------------------------

>Enam-AfroXenR3

--------------------------------------------------------------------------------

--------------------------------------------------------------------------------

--------------------------------------------------------------------------------

--------------------------------------------------------------------------------

--------------------------------------------------------------------------------

--------------------------------------------------------------------------------

--------------------------------------------------------------------------------

--------------------------------------------------------------------------------

--------------------------------------------------------------------------------

--------------------------------------------------------------------------------

--------------------------------------------------------------------------------

--------------------------------------------------------------------------------

--------------------------------------------------------------------------------

--------------------------------------------------------------------------------

--------------------------------------------------------------------------------

--------------------------------------------------------------------------------

--------------------------------------------------------------------------------

--------------------------------------------------------------------------------

--------------------------------------------------------------------------------

-------------------------GAGGATTTTCCATATGGTGAATTTTACC---------------------------

--------------------------------------------------------------------------------

--------------------------------------------------------------------------------

--------------------------------------------------------------------------------

--------------------------------------------------------------------------------

--------------------------------------------------------------------------------

--------------------------------------------------------------------------------

--------------------------------------------------------------------------------

--------------------------------------------------------------------------------

--------------------------------------------------------------------------------

--------------------------------------------------------------------------------

--------------------------------------------------------------------------------

--------------------------------------------------------------------------------

--------------------------------------------------------------------------------

--------------------------------------------------------------------------------

--------------------------------------------------------------------------------

----------------------------------------------------------

>Enam-AfroXenR4

--------------------------------------------------------------------------------

--------------------------------------------------------------------------------

--------------------------------------------------------------------------------

--------------------------------------------------------------------------------

--------------------------------------------------------------------------------

--------------------------------------------------------------------------------

--------------------------------------------------------------------------------

--------------------------------------------------------------------------------

--------------------------------------------------------------------------------

--------------------------------------------------------------------------------

--------------------------------------------------------------------------------

--------------------------------------------------------------------------------

--------------------------------------------------------------------------------

--------------------------------------------------------------------------------

--------------------------------------------------------------------------------

--------------------------------------------------------------------------------

--------------------------------------------------------------------------------

--------------------------------------------------------------------------------

--------------------------------------------------------------------------------

----------------------------------CCAWATGGTGAATTTTAYCMCTGGGGCCC-----------------

--------------------------------------------------------------------------------

--------------------------------------------------------------------------------

--------------------------------------------------------------------------------

--------------------------------------------------------------------------------

--------------------------------------------------------------------------------

--------------------------------------------------------------------------------

--------------------------------------------------------------------------------

--------------------------------------------------------------------------------

--------------------------------------------------------------------------------

--------------------------------------------------------------------------------

--------------------------------------------------------------------------------

--------------------------------------------------------------------------------

--------------------------------------------------------------------------------

--------------------------------------------------------------------------------

--------------------------------------------------------------------------------

----------------------------------------------------------

>Enam-AfroXenF5

--------------------------------------------------------------------------------

--------------------------------------------------------------------------------

--------------------------------------------------------------------------------

--------------------------------------------------------------------------------

--------------------------------------------------------------------------------

--------------------------------------------------------------------------------

--------------------------------------------------------------------------------

--------------------------------------------------------------------------------

--------------------------------------------------------------------------------

--------------------------------------------------------------------------------

--------------------------------------------------------------------------------

--------------------------------------------------------------------------------

--------------------------------------------------------------------------------

--------------------------------------------------------------------------------

--------------------------------------------------------------------------------

--------------------------------------------------------------------------------

--------------------------------------------------------------------------------

-----GAAGAGGACCCARTTGAYCCAACTGGAGATG--------------------------------------------

--------------------------------------------------------------------------------

--------------------------------------------------------------------------------

--------------------------------------------------------------------------------

--------------------------------------------------------------------------------

--------------------------------------------------------------------------------

--------------------------------------------------------------------------------

--------------------------------------------------------------------------------

--------------------------------------------------------------------------------

--------------------------------------------------------------------------------

--------------------------------------------------------------------------------

--------------------------------------------------------------------------------

--------------------------------------------------------------------------------

--------------------------------------------------------------------------------

--------------------------------------------------------------------------------

--------------------------------------------------------------------------------

--------------------------------------------------------------------------------

--------------------------------------------------------------------------------

----------------------------------------------------------

>Enam-AfroXenF6

--------------------------------------------------------------------------------

--------------------------------------------------------------------------------

--------------------------------------------------------------------------------

--------------------------------------------------------------------------------

--------------------------------------------------------------------------------

--------------------------------------------------------------------------------

--------------------------------------------------------------------------------

--------------------------------------------------------------------------------

--------------------------------------------------------------------------------

--------------------------------------------------------------------------------

--------------------------------------------------------------------------------

--------------------------------------------------------------------------------

--------------------------------------------------------------------------------

--------------------------------------------------------------------------------

--------------------------------------------------------------------------------

--------------------------------------------------------------------------------

--------------------------------------------------------------------------------

--------------------GAYCCAACTGGAGATGAAAMTTTCCCAGGAC-----------------------------

--------------------------------------------------------------------------------

--------------------------------------------------------------------------------

--------------------------------------------------------------------------------

--------------------------------------------------------------------------------

--------------------------------------------------------------------------------

--------------------------------------------------------------------------------

--------------------------------------------------------------------------------

--------------------------------------------------------------------------------

--------------------------------------------------------------------------------

--------------------------------------------------------------------------------

--------------------------------------------------------------------------------

--------------------------------------------------------------------------------

--------------------------------------------------------------------------------

--------------------------------------------------------------------------------

--------------------------------------------------------------------------------

--------------------------------------------------------------------------------

--------------------------------------------------------------------------------

----------------------------------------------------------

>Enam-AfroXenIF3

--------------------------------------------------------------------------------

--------------------------------------------------------------------------------

--------------------------------------------------------------------------------

--------------------------------------------------------------------------------

--------------------------------------------------------------------------------

--------------------------------------------------------------------------------

--------------------------------------------------------------------------------

--------------------------------------------------------------------------------

--------------------------------------------------------------------------------

--------------------------------------------------------------------------------

--------------------------------------------------------------------------------

--------------------------------------------------------------------------------

--------------------------------------------------------------------------------

--------------------------------------------------------------------------------

--------------------------------------------------------------------------------

--------------------------------------------------------------------------------

--------------------------------------------------------------------------------

--------------------------------------------------------------------------------

--------------------------------------------------------------------------------

--------------------------------------------------------------------------------

--------------------------------------------------------------------------------

--------------------------------------------------------------------------------

--------------------------------------------------------------------------------

----TAACTMCCCAGHTRGGCTTCRGAAAAAYCC----------------------------------------------

--------------------------------------------------------------------------------

--------------------------------------------------------------------------------

--------------------------------------------------------------------------------

--------------------------------------------------------------------------------

--------------------------------------------------------------------------------

--------------------------------------------------------------------------------

--------------------------------------------------------------------------------

--------------------------------------------------------------------------------

--------------------------------------------------------------------------------

--------------------------------------------------------------------------------

--------------------------------------------------------------------------------

----------------------------------------------------------

>Enam-AfroXenIR3

--------------------------------------------------------------------------------

--------------------------------------------------------------------------------

--------------------------------------------------------------------------------

--------------------------------------------------------------------------------

--------------------------------------------------------------------------------

--------------------------------------------------------------------------------

--------------------------------------------------------------------------------

--------------------------------------------------------------------------------

--------------------------------------------------------------------------------

--------------------------------------------------------------------------------

--------------------------------------------------------------------------------

--------------------------------------------------------------------------------

--------------------------------------------------------------------------------

--------------------------------------------------------------------------------

--------------------------------------------------------------------------------

--------------------------------------------------------------------------------

--------------------------------------------------------------------------------

--------------------------------------------------------------------------------

--------------------------------------------------------------------------------

--------------------------------------------------------------------------------

--------------------------------------------------------------------------------

--------------------------------------------------------------------------------

--------------------------------------------------------------------------------

-----------------------------------------------------------------GGCATGCAAATKACT

AGGTTAAAYTCACCAG----------------------------------------------------------------

--------------------------------------------------------------------------------

--------------------------------------------------------------------------------

--------------------------------------------------------------------------------

--------------------------------------------------------------------------------

--------------------------------------------------------------------------------

--------------------------------------------------------------------------------

--------------------------------------------------------------------------------

--------------------------------------------------------------------------------

--------------------------------------------------------------------------------

--------------------------------------------------------------------------------

----------------------------------------------------------

>Enam-AfroXenR5

--------------------------------------------------------------------------------

--------------------------------------------------------------------------------

--------------------------------------------------------------------------------

--------------------------------------------------------------------------------

--------------------------------------------------------------------------------

--------------------------------------------------------------------------------

--------------------------------------------------------------------------------

--------------------------------------------------------------------------------

--------------------------------------------------------------------------------

--------------------------------------------------------------------------------

--------------------------------------------------------------------------------

--------------------------------------------------------------------------------

--------------------------------------------------------------------------------

--------------------------------------------------------------------------------

--------------------------------------------------------------------------------

--------------------------------------------------------------------------------

--------------------------------------------------------------------------------

--------------------------------------------------------------------------------

--------------------------------------------------------------------------------

--------------------------------------------------------------------------------

--------------------------------------------------------------------------------

--------------------------------------------------------------------------------

--------------------------------------------------------------------------------

--------------------------------------------------------------------------------

--------------------------------------------------------------------------------

--------------------------------------------------------------------------------

--------------------------------------------------------------------------------

---------------------------------------------CAAASAAAYAGCTCAGAGAAGAGRMTGCCTG----

--------------------------------------------------------------------------------

--------------------------------------------------------------------------------

--------------------------------------------------------------------------------

--------------------------------------------------------------------------------

--------------------------------------------------------------------------------

--------------------------------------------------------------------------------

--------------------------------------------------------------------------------

----------------------------------------------------------

>Enam-AfroXenR6

--------------------------------------------------------------------------------

--------------------------------------------------------------------------------

--------------------------------------------------------------------------------

--------------------------------------------------------------------------------

--------------------------------------------------------------------------------

--------------------------------------------------------------------------------

--------------------------------------------------------------------------------

--------------------------------------------------------------------------------

--------------------------------------------------------------------------------

--------------------------------------------------------------------------------

--------------------------------------------------------------------------------

--------------------------------------------------------------------------------

--------------------------------------------------------------------------------

--------------------------------------------------------------------------------

--------------------------------------------------------------------------------

--------------------------------------------------------------------------------

--------------------------------------------------------------------------------

--------------------------------------------------------------------------------

--------------------------------------------------------------------------------

--------------------------------------------------------------------------------

--------------------------------------------------------------------------------

--------------------------------------------------------------------------------

--------------------------------------------------------------------------------

--------------------------------------------------------------------------------

--------------------------------------------------------------------------------

--------------------------------------------------------------------------------

--------------------------------------------------------------------------------

-------------------------------------------------------GCTCAGAGAAGAGRMTGCCTGKGGA

AAGCCAAAAC----------------------------------------------------------------------

--------------------------------------------------------------------------------

--------------------------------------------------------------------------------

--------------------------------------------------------------------------------

--------------------------------------------------------------------------------

--------------------------------------------------------------------------------

--------------------------------------------------------------------------------

----------------------------------------------------------

>Enam-AfroXenF7

--------------------------------------------------------------------------------

--------------------------------------------------------------------------------

--------------------------------------------------------------------------------

--------------------------------------------------------------------------------

--------------------------------------------------------------------------------

--------------------------------------------------------------------------------

--------------------------------------------------------------------------------

--------------------------------------------------------------------------------

--------------------------------------------------------------------------------

--------------------------------------------------------------------------------

--------------------------------------------------------------------------------

--------------------------------------------------------------------------------

--------------------------------------------------------------------------------

--------------------------------------------------------------------------------

--------------------------------------------------------------------------------

--------------------------------------------------------------------------------

--------------------------------------------------------------------------------

--------------------------------------------------------------------------------

--------------------------------------------------------------------------------

--------------------------------------------------------------------------------

--------------------------------------------------------------------------------

--------------------------------------------------------------------------------

--------------------------------------------------------------------------------

-----------------------------------------------------------------GGCATGCAAATTACT

AGGTTAAATTCACC------------------------------------------------------------------

--------------------------------------------------------------------------------

--------------------------------------------------------------------------------

--------------------------------------------------------------------------------

--------------------------------------------------------------------------------

--------------------------------------------------------------------------------

--------------------------------------------------------------------------------

--------------------------------------------------------------------------------

--------------------------------------------------------------------------------

--------------------------------------------------------------------------------

--------------------------------------------------------------------------------

----------------------------------------------------------

>Enam-AfroXenF8

--------------------------------------------------------------------------------

--------------------------------------------------------------------------------

--------------------------------------------------------------------------------

--------------------------------------------------------------------------------

--------------------------------------------------------------------------------

--------------------------------------------------------------------------------

--------------------------------------------------------------------------------

--------------------------------------------------------------------------------

--------------------------------------------------------------------------------

--------------------------------------------------------------------------------

--------------------------------------------------------------------------------

--------------------------------------------------------------------------------

--------------------------------------------------------------------------------

--------------------------------------------------------------------------------

--------------------------------------------------------------------------------

--------------------------------------------------------------------------------

--------------------------------------------------------------------------------

--------------------------------------------------------------------------------

--------------------------------------------------------------------------------

--------------------------------------------------------------------------------

--------------------------------------------------------------------------------

--------------------------------------------------------------------------------

--------------------------------------------------------------------------------

------------------------------------------------------------------------------CT

AGGTTAAATTCACCAGAGGGG-----------------------------------------------------------

--------------------------------------------------------------------------------

--------------------------------------------------------------------------------

--------------------------------------------------------------------------------

--------------------------------------------------------------------------------

--------------------------------------------------------------------------------

--------------------------------------------------------------------------------

--------------------------------------------------------------------------------

--------------------------------------------------------------------------------

--------------------------------------------------------------------------------

--------------------------------------------------------------------------------

----------------------------------------------------------

>Enam-AfroIF4

--------------------------------------------------------------------------------

--------------------------------------------------------------------------------

--------------------------------------------------------------------------------

--------------------------------------------------------------------------------

--------------------------------------------------------------------------------

--------------------------------------------------------------------------------

--------------------------------------------------------------------------------

--------------------------------------------------------------------------------

--------------------------------------------------------------------------------

--------------------------------------------------------------------------------

--------------------------------------------------------------------------------

--------------------------------------------------------------------------------

--------------------------------------------------------------------------------

--------------------------------------------------------------------------------

--------------------------------------------------------------------------------

--------------------------------------------------------------------------------

--------------------------------------------------------------------------------

--------------------------------------------------------------------------------

--------------------------------------------------------------------------------

--------------------------------------------------------------------------------

--------------------------------------------------------------------------------

--------------------------------------------------------------------------------

--------------------------------------------------------------------------------

--------------------------------------------------------------------------------

--------------------------------------------------------------------------------

--------------------------------------------------------------------------------

--------------------------------------------------------------------------------

--------------------------------------------------------------------------------

-----------------------------------CCACTCTGAGGAGGAACACRCCRTGTTC-----------------

--------------------------------------------------------------------------------

--------------------------------------------------------------------------------

--------------------------------------------------------------------------------

--------------------------------------------------------------------------------

--------------------------------------------------------------------------------

--------------------------------------------------------------------------------

----------------------------------------------------------

>Enam-AfroIR4

--------------------------------------------------------------------------------

--------------------------------------------------------------------------------

--------------------------------------------------------------------------------

--------------------------------------------------------------------------------

--------------------------------------------------------------------------------

--------------------------------------------------------------------------------

--------------------------------------------------------------------------------

--------------------------------------------------------------------------------

--------------------------------------------------------------------------------

--------------------------------------------------------------------------------

--------------------------------------------------------------------------------

--------------------------------------------------------------------------------

--------------------------------------------------------------------------------

--------------------------------------------------------------------------------

--------------------------------------------------------------------------------

--------------------------------------------------------------------------------

--------------------------------------------------------------------------------

--------------------------------------------------------------------------------

--------------------------------------------------------------------------------

--------------------------------------------------------------------------------

--------------------------------------------------------------------------------

--------------------------------------------------------------------------------

--------------------------------------------------------------------------------

--------------------------------------------------------------------------------

--------------------------------------------------------------------------------

--------------------------------------------------------------------------------

--------------------------------------------------------------------------------

--------------------------------------------------------------------------------

--------------------------------------------------------------------------------

--------------------------------------------------------------------------------

---------------------------------------------------------------------CCTGAGCAGCT

TGTTMTAGGTACCCC-----------------------------------------------------------------

--------------------------------------------------------------------------------

--------------------------------------------------------------------------------

--------------------------------------------------------------------------------

----------------------------------------------------------

>Enam-AfroXenScanR7

--------------------------------------------------------------------------------

--------------------------------------------------------------------------------

--------------------------------------------------------------------------------

--------------------------------------------------------------------------------

--------------------------------------------------------------------------------

--------------------------------------------------------------------------------

--------------------------------------------------------------------------------

--------------------------------------------------------------------------------

--------------------------------------------------------------------------------

--------------------------------------------------------------------------------

--------------------------------------------------------------------------------

--------------------------------------------------------------------------------

--------------------------------------------------------------------------------

--------------------------------------------------------------------------------

--------------------------------------------------------------------------------

--------------------------------------------------------------------------------

--------------------------------------------------------------------------------

--------------------------------------------------------------------------------

--------------------------------------------------------------------------------

--------------------------------------------------------------------------------

--------------------------------------------------------------------------------

--------------------------------------------------------------------------------

--------------------------------------------------------------------------------

--------------------------------------------------------------------------------

--------------------------------------------------------------------------------

--------------------------------------------------------------------------------

--------------------------------------------------------------------------------

--------------------------------------------------------------------------------

--------------------------------------------------------------------------------

--------------------------------------------------------------------------------

--------------------------------------------------------------------------------

--------------------------------------------------------------------------------

--------------------------------------------------------------------------------

--------------------------------------------------------------------------------

-------------------------------------------------------------------------CCASARG

ACCAGGYACAAGACTG------------------------------------------

>Enam-AfroXenScanR8

--------------------------------------------------------------------------------

--------------------------------------------------------------------------------

--------------------------------------------------------------------------------

--------------------------------------------------------------------------------

--------------------------------------------------------------------------------

--------------------------------------------------------------------------------

--------------------------------------------------------------------------------

--------------------------------------------------------------------------------

--------------------------------------------------------------------------------

--------------------------------------------------------------------------------

--------------------------------------------------------------------------------

--------------------------------------------------------------------------------

--------------------------------------------------------------------------------

--------------------------------------------------------------------------------

--------------------------------------------------------------------------------

--------------------------------------------------------------------------------

--------------------------------------------------------------------------------

--------------------------------------------------------------------------------

--------------------------------------------------------------------------------

--------------------------------------------------------------------------------

--------------------------------------------------------------------------------

--------------------------------------------------------------------------------

--------------------------------------------------------------------------------

--------------------------------------------------------------------------------

--------------------------------------------------------------------------------

--------------------------------------------------------------------------------

--------------------------------------------------------------------------------

--------------------------------------------------------------------------------

--------------------------------------------------------------------------------

--------------------------------------------------------------------------------

--------------------------------------------------------------------------------

--------------------------------------------------------------------------------

--------------------------------------------------------------------------------

--------------------------------------------------------------------------------

--------------------------------------------------------------------------------

-CCAGGYACAAGACTGYTTATTACTTCAGGC---------------------------

**Carnivora and Pholidota Primers**

>Canis

AATCCTTACTTTGGATATTTTGGATATCAGGGATTTGGGGGTCGTCCTCCTTATTATTCAGAAGAGATGTTTGAACAAGA

TTTTGAAAAACCCAAAGAAAAAGATCCTCCTAAAGCAGAGAGTCCGGCCGCTGAGCCCTCGGGTAATTCAACAGGTCCCG

AGACTAATTCTACTCAATCAAATCCTGGAGGGAGTCAGAGTGGAAATGACACCAGCCCAACAGGAAACAGTGGCCCTGGC

CCAAACACTGTGAGTAATCCTACGGCTCAAAACGGGGTTATCTCACCCGCTACAGTTAATATTTCAGGCCAGGGAGTACC

AAGAACTCAAATCTCATGGGGACCAAATCAGCCAAATATTCATGAAAATTATCCAAATCCTAACATCCGAAATTTTCCTG

CAGGGAGACAATGGCGTCCCACTGGTACTTTCATGGGGCACAGACAGAATGGGCCTTTTTACCGAAATCAACAGGTACAA

AGGGGGCCTCGGTGGAACTCCTTTGCTTTGGAACGCAAACAAGCAATGCGTCCAGGAAATCCAATCTATCGTAAGGCTTA

TGCTTCTACTGCGAGAGGGAATTCTCCTAATCATGCAGGAAATCTGGGGAATGTCAGAAGAAAGCCTCAGGCACCAAATA

AACACCCTATGGGAACCAACGTTGCTCCTCTGGGTCCCAAACATGGTACTGTTGTCCACAATGAAAAAATCCAAAATCCA

GGAGAGAAACCAGTAGGCCCAAAAGAAAGAATAGTCATTCCTACAAGGGATCCATCTGGCCCCTGGAGAAACTCTCAAGA

TTATGGAGTTAACAAATCAAACTATAAACTGTCTCCCCCTGAGAGTAATGTGCTAGTCCCAAATTTTAATTCTGTTGATC

AACATGAAAACTCTTATTACTCAAGAGGAGATTCCAGAAGAGCCCCAAATTCTGATGGACAAACCCAAAGCCAGAATTTG

CCCAAAGGGATTATTTTAGAGCCAAGAAGAAACCCGTATGAATCAGAAACTAATCGGCCAGAATTAAAGCACAGTACATA

TCAACCTGTATTCCCTGAGGAAATTCCTCCCCCTGCAAGAGAACATTTTCCTGCTGGAAGAAATACTTGGCATCACCAAG

AAATCTCTCCATCTTTTAAGGAAGATCCTGGGAGGCAGGAGGAACTCTTACCTCCTCCTTCCCAGGGCTCTAGGGGAGGT

GTTTACTACCCTGACTATAACTCTTATGATCCCAGGGAAAATTCACCATACCTTAGAAGCAATAGATGGGATGAGAGAGA

TGATTCTCCCAACACAATAGGGCAACCCAGAAATTCGCTATATCCCATAAATACTCCAGAGCTGAAAGAAACAGTCCCTT

ACAATGAAGAGGACCCAATTGATCCAACTGGAGATGAAACTTTCCCAGGACAAAATAGATGGGGTATGGAAGAGCCAAGC

TTTAAAGAAGGTCCAACAGTTAGGCACTATGAAGGCGAGCAATATACCGTAAATCAACCAAAGGAATACCTTCCCTATTC

CTTAGATAATCCATCAAAAACCAGGGAGGATTTCCCTTATGGTGAGTTTTACCCCTGGAACCCAGATGAGAATTTTCCAT

CATATAATACAGCTCCCACTGTACCACCACCTGTGGAGAGCAGGGGCTACTATGCTAATAATGCTGTCAGACAAGAAGAA

AGCCCTCTGATTCCTTCTTGGAACTCCTGGGACCACAGGGTTCAAGCCCAAGGACAGAAAGAAAGGCAGCCATATTTTAA

CAGAAATTACTGGGATCAGCCAACAACTTTACACAAAGCGCCCCCTAGTCCACCACACCAGAAAGAGAACCAGCCCTATC

CTAGTAATTCCCCAGCTGGGCTTCAGAAAAATCCAACATGGCGTGAAGGTGAGAATTTGAATTACGGCATGCAGATTACT

AGGTTAAATTCACCAGAGGGAGAACATTTGGCTTTCCCAGATTTAATTCCTCCAAGTTACCCAGCAGGTCAAAAAGAAGC

ACATGTATTTCACCTAAGCCAGAGAGGCTCTTGCTGTGCTGGTGGCTCCCCAGGACACAAGGACAATCCACTTGCTCTAC

AGGACTACACTCCACCCTTCGATCTTGCACCAGGGGAGAATGAAGACACCAGTCCTCTGTACACAGAAGATAGTCATGCT

AATCATGCAAGAGATACCATCTCTCCTGCCAGCAACCTACCTGGCCAAAGAAACAGCTCAGAGAAAAGAATGCCTGCAGA

GAGTCAAAACCTAAGTCCTTTTAGAGATGATGTGTCCACTCTGAGAAGGAACACGCCGTGCTCTATGAAGAATCAACTGA

GCCAAAGGGGAATTATGCCGTTTCCTGAAGCCGGTTCCCTTCAATCAAAGAATACACCTTGTCTCACAAGTGATCTTGGA

GGAGATGGCAACAATGTTTTGGAAGAGATATTCGAAGACAACCAGCTCAGTGAAAGAACCGTTGACCTTACTCCTGAACA

ACTTGTTATTGGTACACCTGATGAAGGCCCTGAGCCAGAAGGAATCCAAAGTGAAGTGCAAGGAAATGAGGGTGACAGGC

AGCAACAAAGACCGTCAAGCATCATACAGTTACCATGCTTTGGCTCCAAAATAACAAACTACCACTCCTCCAGCACAGGA

ACTCCATCTAGCATCGGAAGGCAAGGCCCATTTGATGAAGAGCCGATTATGCCTACCGAAAATCCTAACTCATTGTCTAG

GTTAGCTACTGGGGCACAGTTTCAGAGTATAAATGTAGACCCACTTAGTGCTGATGAACACACTCCATTTGATTCTCTTC

AATTAGGGACCAATCCACAGGACCATGTACAAGACTGCTTGCTGCTTCAGGCCTAGGG

>Enam-CarnF1

---CCTTAYTTTGGATATTTTGGATATCAGGG------------------------------------------------

--------------------------------------------------------------------------------

--------------------------------------------------------------------------------

--------------------------------------------------------------------------------

--------------------------------------------------------------------------------

--------------------------------------------------------------------------------

--------------------------------------------------------------------------------

--------------------------------------------------------------------------------

--------------------------------------------------------------------------------

--------------------------------------------------------------------------------

--------------------------------------------------------------------------------

--------------------------------------------------------------------------------

--------------------------------------------------------------------------------

--------------------------------------------------------------------------------

--------------------------------------------------------------------------------

--------------------------------------------------------------------------------

--------------------------------------------------------------------------------

--------------------------------------------------------------------------------

--------------------------------------------------------------------------------

--------------------------------------------------------------------------------

--------------------------------------------------------------------------------

--------------------------------------------------------------------------------

--------------------------------------------------------------------------------

--------------------------------------------------------------------------------

--------------------------------------------------------------------------------

--------------------------------------------------------------------------------

--------------------------------------------------------------------------------

--------------------------------------------------------------------------------

--------------------------------------------------------------------------------

--------------------------------------------------------------------------------

--------------------------------------------------------------------------------

--------------------------------------------------------------------------------

--------------------------------------------------------------------------------

--------------------------------------------------------------------------------

--------------------------------------------------------------------------------

----------------------------------------------------------

>Enam-CarnF2

------------GGATATTTTGGATATCAGGGATTTGGGGG---------------------------------------

--------------------------------------------------------------------------------

--------------------------------------------------------------------------------

--------------------------------------------------------------------------------

--------------------------------------------------------------------------------

--------------------------------------------------------------------------------

--------------------------------------------------------------------------------

--------------------------------------------------------------------------------

--------------------------------------------------------------------------------

--------------------------------------------------------------------------------

--------------------------------------------------------------------------------

--------------------------------------------------------------------------------

--------------------------------------------------------------------------------

--------------------------------------------------------------------------------

--------------------------------------------------------------------------------

--------------------------------------------------------------------------------

--------------------------------------------------------------------------------

--------------------------------------------------------------------------------

--------------------------------------------------------------------------------

--------------------------------------------------------------------------------

--------------------------------------------------------------------------------

--------------------------------------------------------------------------------

--------------------------------------------------------------------------------

--------------------------------------------------------------------------------

--------------------------------------------------------------------------------

--------------------------------------------------------------------------------

--------------------------------------------------------------------------------

--------------------------------------------------------------------------------

--------------------------------------------------------------------------------

--------------------------------------------------------------------------------

--------------------------------------------------------------------------------

--------------------------------------------------------------------------------

--------------------------------------------------------------------------------

--------------------------------------------------------------------------------

--------------------------------------------------------------------------------

----------------------------------------------------------

>Enam-CarnIF1

--------------------------------------------------------------------------------

--------------------------------------------------------------------------------

--------------------------------------------------------------------------------

--------------------------------------------------------------------------------

----------------------------------------------------CCAAATCCTAACATCCGAAATTTTCCTG

CAGG----------------------------------------------------------------------------

--------------------------------------------------------------------------------

--------------------------------------------------------------------------------

--------------------------------------------------------------------------------

--------------------------------------------------------------------------------

--------------------------------------------------------------------------------

--------------------------------------------------------------------------------

--------------------------------------------------------------------------------

--------------------------------------------------------------------------------

--------------------------------------------------------------------------------

--------------------------------------------------------------------------------

--------------------------------------------------------------------------------

--------------------------------------------------------------------------------

--------------------------------------------------------------------------------

--------------------------------------------------------------------------------

--------------------------------------------------------------------------------

--------------------------------------------------------------------------------

--------------------------------------------------------------------------------

--------------------------------------------------------------------------------

--------------------------------------------------------------------------------

--------------------------------------------------------------------------------

--------------------------------------------------------------------------------

--------------------------------------------------------------------------------

--------------------------------------------------------------------------------

--------------------------------------------------------------------------------

--------------------------------------------------------------------------------

--------------------------------------------------------------------------------

--------------------------------------------------------------------------------

--------------------------------------------------------------------------------

--------------------------------------------------------------------------------

----------------------------------------------------------

>Enam-CarnIR1

--------------------------------------------------------------------------------

--------------------------------------------------------------------------------

--------------------------------------------------------------------------------

--------------------------------------------------------------------------------

--------------------------------------------------------------------------------

--------------------------------------------------------------------------------

--------------------------------------------------------------------------------

----------------------------------------------------GTCAGAAGAAAGCCTCAGGSACCAAATA

AACACCC-------------------------------------------------------------------------

--------------------------------------------------------------------------------

--------------------------------------------------------------------------------

--------------------------------------------------------------------------------

--------------------------------------------------------------------------------

--------------------------------------------------------------------------------

--------------------------------------------------------------------------------

--------------------------------------------------------------------------------

--------------------------------------------------------------------------------

--------------------------------------------------------------------------------

--------------------------------------------------------------------------------

--------------------------------------------------------------------------------

--------------------------------------------------------------------------------

--------------------------------------------------------------------------------

--------------------------------------------------------------------------------

--------------------------------------------------------------------------------

--------------------------------------------------------------------------------

--------------------------------------------------------------------------------

--------------------------------------------------------------------------------

--------------------------------------------------------------------------------

--------------------------------------------------------------------------------

--------------------------------------------------------------------------------

--------------------------------------------------------------------------------

--------------------------------------------------------------------------------

--------------------------------------------------------------------------------

--------------------------------------------------------------------------------

--------------------------------------------------------------------------------

----------------------------------------------------------

>Enam-CarnR1

--------------------------------------------------------------------------------

--------------------------------------------------------------------------------

--------------------------------------------------------------------------------

--------------------------------------------------------------------------------

--------------------------------------------------------------------------------

--------------------------------------------------------------------------------

--------------------------------------------------------------------------------

--------------------------------------------------------------------------------

--------------------------------------------------------------------------------

--------------------------------------------------------------------------------

--------------------------------------------------------------------------------

-----------------------------------------------------------------------CAGAATTTG

CCCAAAGGGATTATTTTRGAGCC---------------------------------------------------------

--------------------------------------------------------------------------------

--------------------------------------------------------------------------------

--------------------------------------------------------------------------------

--------------------------------------------------------------------------------

--------------------------------------------------------------------------------

--------------------------------------------------------------------------------

--------------------------------------------------------------------------------

--------------------------------------------------------------------------------

--------------------------------------------------------------------------------

--------------------------------------------------------------------------------

--------------------------------------------------------------------------------

--------------------------------------------------------------------------------

--------------------------------------------------------------------------------

--------------------------------------------------------------------------------

--------------------------------------------------------------------------------

--------------------------------------------------------------------------------

--------------------------------------------------------------------------------

--------------------------------------------------------------------------------

--------------------------------------------------------------------------------

--------------------------------------------------------------------------------

--------------------------------------------------------------------------------

--------------------------------------------------------------------------------

----------------------------------------------------------

>Enam-CarnR2

--------------------------------------------------------------------------------

--------------------------------------------------------------------------------

--------------------------------------------------------------------------------

--------------------------------------------------------------------------------

--------------------------------------------------------------------------------

--------------------------------------------------------------------------------

--------------------------------------------------------------------------------

--------------------------------------------------------------------------------

--------------------------------------------------------------------------------

--------------------------------------------------------------------------------

--------------------------------------------------------------------------------

--------------------------------------------------------------------------------

-------GGATTATTTTRGAGCCAAGAAGAAWCCC---------------------------------------------

--------------------------------------------------------------------------------

--------------------------------------------------------------------------------

--------------------------------------------------------------------------------

--------------------------------------------------------------------------------

--------------------------------------------------------------------------------

--------------------------------------------------------------------------------

--------------------------------------------------------------------------------

--------------------------------------------------------------------------------

--------------------------------------------------------------------------------

--------------------------------------------------------------------------------

--------------------------------------------------------------------------------

--------------------------------------------------------------------------------

--------------------------------------------------------------------------------

--------------------------------------------------------------------------------

--------------------------------------------------------------------------------

--------------------------------------------------------------------------------

--------------------------------------------------------------------------------

--------------------------------------------------------------------------------

--------------------------------------------------------------------------------

--------------------------------------------------------------------------------

--------------------------------------------------------------------------------

--------------------------------------------------------------------------------

----------------------------------------------------------

>Enam-CarnF3

--------------------------------------------------------------------------------

--------------------------------------------------------------------------------

--------------------------------------------------------------------------------

--------------------------------------------------------------------------------

--------------------------------------------------------------------------------

--------------------------------------------------------------------------------

--------------------------------------------------------------------------------

--------------------------------------------------------------------------------

--------------------------------------------------------------------------------

---------------------------------------CCTACAAGRGATCCATCTGGCCCCTGGAGAAAC--------

--------------------------------------------------------------------------------

--------------------------------------------------------------------------------

--------------------------------------------------------------------------------

--------------------------------------------------------------------------------

--------------------------------------------------------------------------------

--------------------------------------------------------------------------------

--------------------------------------------------------------------------------

--------------------------------------------------------------------------------

--------------------------------------------------------------------------------

--------------------------------------------------------------------------------

--------------------------------------------------------------------------------

--------------------------------------------------------------------------------

--------------------------------------------------------------------------------

--------------------------------------------------------------------------------

--------------------------------------------------------------------------------

--------------------------------------------------------------------------------

--------------------------------------------------------------------------------

--------------------------------------------------------------------------------

--------------------------------------------------------------------------------

--------------------------------------------------------------------------------

--------------------------------------------------------------------------------

--------------------------------------------------------------------------------

--------------------------------------------------------------------------------

--------------------------------------------------------------------------------

--------------------------------------------------------------------------------

----------------------------------------------------------

>Enam-CarnF4

--------------------------------------------------------------------------------

--------------------------------------------------------------------------------

--------------------------------------------------------------------------------

--------------------------------------------------------------------------------

--------------------------------------------------------------------------------

--------------------------------------------------------------------------------

--------------------------------------------------------------------------------

--------------------------------------------------------------------------------

--------------------------------------------------------------------------------

---------------------------------------------------------GGCCCCTGGAGAAACTCTCAAGA

TTATGG--------------------------------------------------------------------------

--------------------------------------------------------------------------------

--------------------------------------------------------------------------------

--------------------------------------------------------------------------------

--------------------------------------------------------------------------------

--------------------------------------------------------------------------------

--------------------------------------------------------------------------------

--------------------------------------------------------------------------------

--------------------------------------------------------------------------------

--------------------------------------------------------------------------------

--------------------------------------------------------------------------------

--------------------------------------------------------------------------------

--------------------------------------------------------------------------------

--------------------------------------------------------------------------------

--------------------------------------------------------------------------------

--------------------------------------------------------------------------------

--------------------------------------------------------------------------------

--------------------------------------------------------------------------------

--------------------------------------------------------------------------------

--------------------------------------------------------------------------------

--------------------------------------------------------------------------------

--------------------------------------------------------------------------------

--------------------------------------------------------------------------------

--------------------------------------------------------------------------------

--------------------------------------------------------------------------------

----------------------------------------------------------

>Enam-CarnIF2

--------------------------------------------------------------------------------

--------------------------------------------------------------------------------

--------------------------------------------------------------------------------

--------------------------------------------------------------------------------

--------------------------------------------------------------------------------

--------------------------------------------------------------------------------

--------------------------------------------------------------------------------

--------------------------------------------------------------------------------

--------------------------------------------------------------------------------

--------------------------------------------------------------------------------

--------------------------------------------------------------------------------

--------------------------------------------------------------------------------

--------------------------------------------------------------------------------

--------------------------------CTGCAAGAGAACATTTTCCTGCTGGAAG--------------------

--------------------------------------------------------------------------------

--------------------------------------------------------------------------------

--------------------------------------------------------------------------------

--------------------------------------------------------------------------------

--------------------------------------------------------------------------------

--------------------------------------------------------------------------------

--------------------------------------------------------------------------------

--------------------------------------------------------------------------------

--------------------------------------------------------------------------------

--------------------------------------------------------------------------------

--------------------------------------------------------------------------------

--------------------------------------------------------------------------------

--------------------------------------------------------------------------------

--------------------------------------------------------------------------------

--------------------------------------------------------------------------------

--------------------------------------------------------------------------------

--------------------------------------------------------------------------------

--------------------------------------------------------------------------------

--------------------------------------------------------------------------------

--------------------------------------------------------------------------------

--------------------------------------------------------------------------------

----------------------------------------------------------

>Enam-CarnIR2

--------------------------------------------------------------------------------

--------------------------------------------------------------------------------

--------------------------------------------------------------------------------

--------------------------------------------------------------------------------

--------------------------------------------------------------------------------

--------------------------------------------------------------------------------

--------------------------------------------------------------------------------

--------------------------------------------------------------------------------

--------------------------------------------------------------------------------

--------------------------------------------------------------------------------

--------------------------------------------------------------------------------

--------------------------------------------------------------------------------

--------------------------------------------------------------------------------

--------------------------------------------------------------------------------

--------------------------------------------------------------------------------

----------------------CTTATGATCCCAGGGAAAATTCACCATAC-----------------------------

--------------------------------------------------------------------------------

--------------------------------------------------------------------------------

--------------------------------------------------------------------------------

--------------------------------------------------------------------------------

--------------------------------------------------------------------------------

--------------------------------------------------------------------------------

--------------------------------------------------------------------------------

--------------------------------------------------------------------------------

--------------------------------------------------------------------------------

--------------------------------------------------------------------------------

--------------------------------------------------------------------------------

--------------------------------------------------------------------------------

--------------------------------------------------------------------------------

--------------------------------------------------------------------------------

--------------------------------------------------------------------------------

--------------------------------------------------------------------------------

--------------------------------------------------------------------------------

--------------------------------------------------------------------------------

--------------------------------------------------------------------------------

----------------------------------------------------------

>Enam-CarnR3

--------------------------------------------------------------------------------

--------------------------------------------------------------------------------

--------------------------------------------------------------------------------

--------------------------------------------------------------------------------

--------------------------------------------------------------------------------

--------------------------------------------------------------------------------

--------------------------------------------------------------------------------

--------------------------------------------------------------------------------

--------------------------------------------------------------------------------

--------------------------------------------------------------------------------

--------------------------------------------------------------------------------

--------------------------------------------------------------------------------

--------------------------------------------------------------------------------

--------------------------------------------------------------------------------

--------------------------------------------------------------------------------

--------------------------------------------------------------------------------

--------------------------------------------------------------------------------

--------------------------------------------------------------------------------

--------------------------------------------------------------------------------

--------------------CCAGGGAGGATTTCCCTTATGGTGAGTTTTACC---------------------------

--------------------------------------------------------------------------------

--------------------------------------------------------------------------------

--------------------------------------------------------------------------------

--------------------------------------------------------------------------------

--------------------------------------------------------------------------------

--------------------------------------------------------------------------------

--------------------------------------------------------------------------------

--------------------------------------------------------------------------------

--------------------------------------------------------------------------------

--------------------------------------------------------------------------------

--------------------------------------------------------------------------------

--------------------------------------------------------------------------------

--------------------------------------------------------------------------------

--------------------------------------------------------------------------------

--------------------------------------------------------------------------------

----------------------------------------------------------

>Enam-CarnR4

--------------------------------------------------------------------------------

--------------------------------------------------------------------------------

--------------------------------------------------------------------------------

--------------------------------------------------------------------------------

--------------------------------------------------------------------------------

--------------------------------------------------------------------------------

--------------------------------------------------------------------------------

--------------------------------------------------------------------------------

--------------------------------------------------------------------------------

--------------------------------------------------------------------------------

--------------------------------------------------------------------------------

--------------------------------------------------------------------------------

--------------------------------------------------------------------------------

--------------------------------------------------------------------------------

--------------------------------------------------------------------------------

--------------------------------------------------------------------------------

--------------------------------------------------------------------------------

--------------------------------------------------------------------------------

--------------------------------------------------------------------------------

----------------------------------CCTTATGGTGAGTTTTACCCCTGGAACCCAG---------------

--------------------------------------------------------------------------------

--------------------------------------------------------------------------------

--------------------------------------------------------------------------------

--------------------------------------------------------------------------------

--------------------------------------------------------------------------------

--------------------------------------------------------------------------------

--------------------------------------------------------------------------------

--------------------------------------------------------------------------------

--------------------------------------------------------------------------------

--------------------------------------------------------------------------------

--------------------------------------------------------------------------------

--------------------------------------------------------------------------------

--------------------------------------------------------------------------------

--------------------------------------------------------------------------------

--------------------------------------------------------------------------------

----------------------------------------------------------

>Enam-CarnF5

--------------------------------------------------------------------------------

--------------------------------------------------------------------------------

--------------------------------------------------------------------------------

--------------------------------------------------------------------------------

--------------------------------------------------------------------------------

--------------------------------------------------------------------------------

--------------------------------------------------------------------------------

--------------------------------------------------------------------------------

--------------------------------------------------------------------------------

--------------------------------------------------------------------------------

--------------------------------------------------------------------------------

--------------------------------------------------------------------------------

--------------------------------------------------------------------------------

--------------------------------------------------------------------------------

--------------------------------------------------------------------------------

--------------------------------------------------------------------------------

--------------------------------------------------------------------------------

--------------------GATCCAACTGGAGATGAAACTTTCCCAGGAC-----------------------------

--------------------------------------------------------------------------------

--------------------------------------------------------------------------------

--------------------------------------------------------------------------------

--------------------------------------------------------------------------------

--------------------------------------------------------------------------------

--------------------------------------------------------------------------------

--------------------------------------------------------------------------------

--------------------------------------------------------------------------------

--------------------------------------------------------------------------------

--------------------------------------------------------------------------------

--------------------------------------------------------------------------------

--------------------------------------------------------------------------------

--------------------------------------------------------------------------------

--------------------------------------------------------------------------------

--------------------------------------------------------------------------------

--------------------------------------------------------------------------------

--------------------------------------------------------------------------------

----------------------------------------------------------

>Enam-CarnF6

--------------------------------------------------------------------------------

--------------------------------------------------------------------------------

--------------------------------------------------------------------------------

--------------------------------------------------------------------------------

--------------------------------------------------------------------------------

--------------------------------------------------------------------------------

--------------------------------------------------------------------------------

--------------------------------------------------------------------------------

--------------------------------------------------------------------------------

--------------------------------------------------------------------------------

--------------------------------------------------------------------------------

--------------------------------------------------------------------------------

--------------------------------------------------------------------------------

--------------------------------------------------------------------------------

--------------------------------------------------------------------------------

--------------------------------------------------------------------------------

--------------------------------------------------------------------------------

------------------------------GAGATGAAACTTTCCCAGGACAAAATAGATGG------------------

--------------------------------------------------------------------------------

--------------------------------------------------------------------------------

--------------------------------------------------------------------------------

--------------------------------------------------------------------------------

--------------------------------------------------------------------------------

--------------------------------------------------------------------------------

--------------------------------------------------------------------------------

--------------------------------------------------------------------------------

--------------------------------------------------------------------------------

--------------------------------------------------------------------------------

--------------------------------------------------------------------------------

--------------------------------------------------------------------------------

--------------------------------------------------------------------------------

--------------------------------------------------------------------------------

--------------------------------------------------------------------------------

--------------------------------------------------------------------------------

--------------------------------------------------------------------------------

----------------------------------------------------------

>Enam-CarnIF3

--------------------------------------------------------------------------------

--------------------------------------------------------------------------------

--------------------------------------------------------------------------------

--------------------------------------------------------------------------------

--------------------------------------------------------------------------------

--------------------------------------------------------------------------------

--------------------------------------------------------------------------------

--------------------------------------------------------------------------------

--------------------------------------------------------------------------------

--------------------------------------------------------------------------------

--------------------------------------------------------------------------------

--------------------------------------------------------------------------------

--------------------------------------------------------------------------------

--------------------------------------------------------------------------------

--------------------------------------------------------------------------------

--------------------------------------------------------------------------------

--------------------------------------------------------------------------------

--------------------------------------------------------------------------------

--------------------------------------------------------------------------------

--------------------------------------------------------------------------------

--------------------------------------------------------------------------------

--------------------------------------------------------------------------------

---------------------------------------------------------------------CCAGCCYTATC

CTAGYAATTCCCCAGCTGG-------------------------------------------------------------

--------------------------------------------------------------------------------

--------------------------------------------------------------------------------

--------------------------------------------------------------------------------

--------------------------------------------------------------------------------

--------------------------------------------------------------------------------

--------------------------------------------------------------------------------

--------------------------------------------------------------------------------

--------------------------------------------------------------------------------

--------------------------------------------------------------------------------

--------------------------------------------------------------------------------

--------------------------------------------------------------------------------

----------------------------------------------------------

>Enam-CarnIR3

--------------------------------------------------------------------------------

--------------------------------------------------------------------------------

--------------------------------------------------------------------------------

--------------------------------------------------------------------------------

--------------------------------------------------------------------------------

--------------------------------------------------------------------------------

--------------------------------------------------------------------------------

--------------------------------------------------------------------------------

--------------------------------------------------------------------------------

--------------------------------------------------------------------------------

--------------------------------------------------------------------------------

--------------------------------------------------------------------------------

--------------------------------------------------------------------------------

--------------------------------------------------------------------------------

--------------------------------------------------------------------------------

--------------------------------------------------------------------------------

--------------------------------------------------------------------------------

--------------------------------------------------------------------------------

--------------------------------------------------------------------------------

--------------------------------------------------------------------------------

--------------------------------------------------------------------------------

--------------------------------------------------------------------------------

--------------------------------------------------------------------------------

--------------------------------------------------------------------------------

------------------------------GCTTTCCCAGAYTTAATTCCTCCAAGTTACCC------------------

--------------------------------------------------------------------------------

--------------------------------------------------------------------------------

--------------------------------------------------------------------------------

--------------------------------------------------------------------------------

--------------------------------------------------------------------------------

--------------------------------------------------------------------------------

--------------------------------------------------------------------------------

--------------------------------------------------------------------------------

--------------------------------------------------------------------------------

--------------------------------------------------------------------------------

----------------------------------------------------------

>Enam-CarnR5

--------------------------------------------------------------------------------

--------------------------------------------------------------------------------

--------------------------------------------------------------------------------

--------------------------------------------------------------------------------

--------------------------------------------------------------------------------

--------------------------------------------------------------------------------

--------------------------------------------------------------------------------

--------------------------------------------------------------------------------

--------------------------------------------------------------------------------

--------------------------------------------------------------------------------

--------------------------------------------------------------------------------

--------------------------------------------------------------------------------

--------------------------------------------------------------------------------

--------------------------------------------------------------------------------

--------------------------------------------------------------------------------

--------------------------------------------------------------------------------

--------------------------------------------------------------------------------

--------------------------------------------------------------------------------

--------------------------------------------------------------------------------

--------------------------------------------------------------------------------

--------------------------------------------------------------------------------

--------------------------------------------------------------------------------

--------------------------------------------------------------------------------

--------------------------------------------------------------------------------

--------------------------------------------------------------------------------

--------------------------------------------------------------------------------

--------------------------------------------------------------------------------

---CATGCAAGABAYACCATCTCYCCTSCMAGC-----------------------------------------------

--------------------------------------------------------------------------------

--------------------------------------------------------------------------------

--------------------------------------------------------------------------------

--------------------------------------------------------------------------------

--------------------------------------------------------------------------------

--------------------------------------------------------------------------------

--------------------------------------------------------------------------------

----------------------------------------------------------

>Enam-CarnR6

--------------------------------------------------------------------------------

--------------------------------------------------------------------------------

--------------------------------------------------------------------------------

--------------------------------------------------------------------------------

--------------------------------------------------------------------------------

--------------------------------------------------------------------------------

--------------------------------------------------------------------------------

--------------------------------------------------------------------------------

--------------------------------------------------------------------------------

--------------------------------------------------------------------------------

--------------------------------------------------------------------------------

--------------------------------------------------------------------------------

--------------------------------------------------------------------------------

--------------------------------------------------------------------------------

--------------------------------------------------------------------------------

--------------------------------------------------------------------------------

--------------------------------------------------------------------------------

--------------------------------------------------------------------------------

--------------------------------------------------------------------------------

--------------------------------------------------------------------------------

--------------------------------------------------------------------------------

--------------------------------------------------------------------------------

--------------------------------------------------------------------------------

--------------------------------------------------------------------------------

--------------------------------------------------------------------------------

--------------------------------------------------------------------------------

--------------------------------------------------------------------------------

----------------CCATCTCYCCTGCMAGCAACCTACCTGGCC----------------------------------

--------------------------------------------------------------------------------

--------------------------------------------------------------------------------

--------------------------------------------------------------------------------

--------------------------------------------------------------------------------

--------------------------------------------------------------------------------

--------------------------------------------------------------------------------

--------------------------------------------------------------------------------

----------------------------------------------------------

>Enam-CarnF7

--------------------------------------------------------------------------------

--------------------------------------------------------------------------------

--------------------------------------------------------------------------------

--------------------------------------------------------------------------------

--------------------------------------------------------------------------------

--------------------------------------------------------------------------------

--------------------------------------------------------------------------------

--------------------------------------------------------------------------------

--------------------------------------------------------------------------------

--------------------------------------------------------------------------------

--------------------------------------------------------------------------------

--------------------------------------------------------------------------------

--------------------------------------------------------------------------------

--------------------------------------------------------------------------------

--------------------------------------------------------------------------------

--------------------------------------------------------------------------------

--------------------------------------------------------------------------------

--------------------------------------------------------------------------------

--------------------------------------------------------------------------------

--------------------------------------------------------------------------------

--------------------------------------------------------------------------------

--------------------------------------------------------------------------------

--------------------------------------------------------------------------------

--------------------------------CCAACATGGCRTGAARGTGAGAATTTG---------------------

--------------------------------------------------------------------------------

--------------------------------------------------------------------------------

--------------------------------------------------------------------------------

--------------------------------------------------------------------------------

--------------------------------------------------------------------------------

--------------------------------------------------------------------------------

--------------------------------------------------------------------------------

--------------------------------------------------------------------------------

--------------------------------------------------------------------------------

--------------------------------------------------------------------------------

--------------------------------------------------------------------------------

----------------------------------------------------------

>Enam-CarnF8

--------------------------------------------------------------------------------

--------------------------------------------------------------------------------

--------------------------------------------------------------------------------

--------------------------------------------------------------------------------

--------------------------------------------------------------------------------

--------------------------------------------------------------------------------

--------------------------------------------------------------------------------

--------------------------------------------------------------------------------

--------------------------------------------------------------------------------

--------------------------------------------------------------------------------

--------------------------------------------------------------------------------

--------------------------------------------------------------------------------

--------------------------------------------------------------------------------

--------------------------------------------------------------------------------

--------------------------------------------------------------------------------

--------------------------------------------------------------------------------

--------------------------------------------------------------------------------

--------------------------------------------------------------------------------

--------------------------------------------------------------------------------

--------------------------------------------------------------------------------

--------------------------------------------------------------------------------

--------------------------------------------------------------------------------

--------------------------------------------------------------------------------

--------------------------------------------GAAGGTGAGAATTTGAATTAYGGCATGC--------

--------------------------------------------------------------------------------

--------------------------------------------------------------------------------

--------------------------------------------------------------------------------

--------------------------------------------------------------------------------

--------------------------------------------------------------------------------

--------------------------------------------------------------------------------

--------------------------------------------------------------------------------

--------------------------------------------------------------------------------

--------------------------------------------------------------------------------

--------------------------------------------------------------------------------

--------------------------------------------------------------------------------

----------------------------------------------------------

>Enam-CarnIF4

--------------------------------------------------------------------------------

--------------------------------------------------------------------------------

--------------------------------------------------------------------------------

--------------------------------------------------------------------------------

--------------------------------------------------------------------------------

--------------------------------------------------------------------------------

--------------------------------------------------------------------------------

--------------------------------------------------------------------------------

--------------------------------------------------------------------------------

--------------------------------------------------------------------------------

--------------------------------------------------------------------------------

--------------------------------------------------------------------------------

--------------------------------------------------------------------------------

--------------------------------------------------------------------------------

--------------------------------------------------------------------------------

--------------------------------------------------------------------------------

--------------------------------------------------------------------------------

--------------------------------------------------------------------------------

--------------------------------------------------------------------------------

--------------------------------------------------------------------------------

--------------------------------------------------------------------------------

--------------------------------------------------------------------------------

--------------------------------------------------------------------------------

--------------------------------------------------------------------------------

--------------------------------------------------------------------------------

--------------------------------------------------------------------------------

--------------------------------------------------------------------------------

--------------------------------------------------------------------------------

--------------GTCCTTTTAGAGATGATGTGTCCACYCTGAG-----------------------------------

--------------------------------------------------------------------------------

--------------------------------------------------------------------------------

--------------------------------------------------------------------------------

--------------------------------------------------------------------------------

--------------------------------------------------------------------------------

--------------------------------------------------------------------------------

----------------------------------------------------------

>Enam-CarnIR4

--------------------------------------------------------------------------------

--------------------------------------------------------------------------------

--------------------------------------------------------------------------------

--------------------------------------------------------------------------------

--------------------------------------------------------------------------------

--------------------------------------------------------------------------------

--------------------------------------------------------------------------------

--------------------------------------------------------------------------------

--------------------------------------------------------------------------------

--------------------------------------------------------------------------------

--------------------------------------------------------------------------------

--------------------------------------------------------------------------------

--------------------------------------------------------------------------------

--------------------------------------------------------------------------------

--------------------------------------------------------------------------------

--------------------------------------------------------------------------------

--------------------------------------------------------------------------------

--------------------------------------------------------------------------------

--------------------------------------------------------------------------------

--------------------------------------------------------------------------------

--------------------------------------------------------------------------------

--------------------------------------------------------------------------------

--------------------------------------------------------------------------------

--------------------------------------------------------------------------------

--------------------------------------------------------------------------------

--------------------------------------------------------------------------------

--------------------------------------------------------------------------------

--------------------------------------------------------------------------------

--------------------------------------------------------------------------------

-CCAAAGGGGAATTATGCCSTTTCCTGAAGCC------------------------------------------------

--------------------------------------------------------------------------------

--------------------------------------------------------------------------------

--------------------------------------------------------------------------------

--------------------------------------------------------------------------------

--------------------------------------------------------------------------------

----------------------------------------------------------

>Enam-CarnR7

--------------------------------------------------------------------------------

--------------------------------------------------------------------------------

--------------------------------------------------------------------------------

--------------------------------------------------------------------------------

--------------------------------------------------------------------------------

--------------------------------------------------------------------------------

--------------------------------------------------------------------------------

--------------------------------------------------------------------------------

--------------------------------------------------------------------------------

--------------------------------------------------------------------------------

--------------------------------------------------------------------------------

--------------------------------------------------------------------------------

--------------------------------------------------------------------------------

--------------------------------------------------------------------------------

--------------------------------------------------------------------------------

--------------------------------------------------------------------------------

--------------------------------------------------------------------------------

--------------------------------------------------------------------------------

--------------------------------------------------------------------------------

--------------------------------------------------------------------------------

--------------------------------------------------------------------------------

--------------------------------------------------------------------------------

--------------------------------------------------------------------------------

--------------------------------------------------------------------------------

--------------------------------------------------------------------------------

--------------------------------------------------------------------------------

--------------------------------------------------------------------------------

--------------------------------------------------------------------------------

--------------------------------------------------------------------------------

--------------------------------------------------------------------------------

--------------------------------------------------------------------------------

--------------------------------------------------------------------------------

--------------------------------------------------------------------------------

--------------------------------------------------------------------------------

--------------------------------------------------------------------------------

-------GACCAATCCACAGGACCATGTRCAAGAC-----------------------

>Enam-CarnR8

--------------------------------------------------------------------------------

--------------------------------------------------------------------------------

--------------------------------------------------------------------------------

--------------------------------------------------------------------------------

--------------------------------------------------------------------------------

--------------------------------------------------------------------------------

--------------------------------------------------------------------------------

--------------------------------------------------------------------------------

--------------------------------------------------------------------------------

--------------------------------------------------------------------------------

--------------------------------------------------------------------------------

--------------------------------------------------------------------------------

--------------------------------------------------------------------------------

--------------------------------------------------------------------------------

--------------------------------------------------------------------------------

--------------------------------------------------------------------------------

--------------------------------------------------------------------------------

--------------------------------------------------------------------------------

--------------------------------------------------------------------------------

--------------------------------------------------------------------------------

--------------------------------------------------------------------------------

--------------------------------------------------------------------------------

--------------------------------------------------------------------------------

--------------------------------------------------------------------------------

--------------------------------------------------------------------------------

--------------------------------------------------------------------------------

--------------------------------------------------------------------------------

--------------------------------------------------------------------------------

--------------------------------------------------------------------------------

--------------------------------------------------------------------------------

--------------------------------------------------------------------------------

--------------------------------------------------------------------------------

--------------------------------------------------------------------------------

--------------------------------------------------------------------------------

--------------------------------------------------------------------------------

--------------------GACCATGTRCAAGACTGCTTRCTRCTTCAGGCC-----

**Cetartiodactyla Primers**

>Bos

AACCCTTTCTTTGGATTTTTTGGATATCATGGATTTGGGGGCCGTCCTCCTTATTATTCAGAAGAGATGTTTGAAGATTT

TGAAAAACCAAAAGAAGAAGATCCTCCTAAAACAGAGACTCCAGCCACAGATCCTTCTGTTAATTCAACAGTTCCTGAGA

CTAACTCTACCCAGCCAGGTGCACCCAGTCCCAGAGCGGGTCAAGGCGGAAATGACACCAGCCCAGCAGGAAACAATGGT

CAGGACCCTAACACTGTGAGCAACCCTACAGTTCAAAACAACCCTGTAGTAAATGTTTCCGGCCAAGGAGTTCCAAGAAG

TCAAACCCCGTGGAGACCAAGTCAGACAAATATTCATGAAAATTATCCATATCCTAACATTCGAAATTTTCCTGCAGGAA

GACAATGGCATCCTACTGGTACCTCTATGGGGAACAGACGTAATGGGCCTTTTTACCGAAATCAACAAATTCAAAGGGCT

CCTCGGTGGAACTCCTTTGTTTTGGAAGGCAAACAAGCAATTCGTCTAGGATATCCAATATATCGCAGAGCTTATGCCTC

CACCGTAAGGGGCAATTATCCCAATTATGCAGGAAATCCAGTAAATTTCAGAAGAAAGCCTGAGGGGCCCAGTAAACAAC

CTGAGGGAACTATTGCCCCTCTGGGTCCCAAACATGGTACTACTGGCCACAATGAAAACATCCAAAATCCAAAAGAGAAG

CCAGTAAGTCAAAAAGAAAGAATAGTCATTCCTACAAGGGATCCAAATGGCCCCTGGAGAAACTCTCAAGACTATGGAGT

TACTAAATCAAACTATAAACTGCCTCACCCTGAGGGTAACATTCTAGTCCCAAATTTTAATTCTATAGATCAACATGAAA

ACTCTTATTACCCCAGAGGAGATTCCAGAGGAACCCCAAATTCTAATGGACAAACCCAAAGCCAGAATTTGCCCAAAGGG

ATTATTTTAGAACCAAGAAGAATTCCATATGAATCAGAAATTAATCAGCCAGAAATAAAGCACAGTACACATCAGCCTGT

GTACCCTGAGGGATCTCCTTCCCCTGCAAGGGAACGTTTTCCTGCTGGAAGAAATACTTGGAACCAACAAGAAATCTCTC

CACCTTTTAAGGAAGATCCTGGAAGGAAGGAAGAACACTTACCTCATCCTTCCCTTGGCTCTAGAGGACGTATTTACTAC

ACTGACTACAACCCCTATGACCGCAGGGAAAACCCACCATACCTTAGAAGTAATAGCTGGGATGAAAGAAATGATCCTCC

CAATACAATGGGGCAATCTGAAAATCCACACTATCCCATGAATACTCCAGATCCAAAAGAGACAATCCCTTATAATGAAG

AAGGCCCAGCTGATCCAACTGGAGATGAAACTTTCCCAGGACAAACGAGATGGAGTGTAGATGAGTCGAACTTTAAGACA

GCCCCAACAGCTAGGTATGAAGGCAAGCAATATACCTCAAACCAACCAAAGGAATACTCTCCCTATTCCTTAGATAATCT

ACCAAAACCCAGGGAGTATTTTCCTTACGGTGAATTTTACCCCTGGAGCCCAGATGAGAACTTTCCATCATATAATACTG

CTCCCACTATACCACTGCTTGTGGAGAACAGGGGCTATTATCCTACTAATGCTGTTGGACAAGAAGAAAACACTATGTTT

CCTTCTTGGAATTCCTGGGACCACATGGTTCAAGTCCAAGGGCAGAAAGAAAGGAGGCCATATTTTACCAGAACTTTCTG

GGGTCAGCCAACGAATCTACCCAAAGCCCCTGCTAGTCCACCATACCACAAGGAGAATCAGCCTTATTTCAGTAACTCCC

CAACTGGGCTTCAGAAAGATCCAACATGGCATGAAGGTGAGAATTTGAATTATGGCATGCAAATCACTAGGTTAAATTCA

CCAGAGGGAGGACATTTGGCTTTTCCAGACTTAATTCCTCCACATTACCCAGGAAGTCAAAAAGAAACACGTCTATTTCA

CCTAAGTCAGCGAGGCCCTTGCTGTGCTGGTGGCTCCATAGGGCCCAAGGACAATCCACTTGCTCTACAAGACTACACTC

TTTTTTTTGGTCTTGCACCAGGGGAAAACCAAGACACCAGCCCTCTCTATACAGAAGACAGTCATACTAAGCATGAAAGA

TATACTATTTCCCCAACAAGCATCCTACCTGGCCAAAGAAACAGCTCAGAAAAAAGACTTCCTGGAGAAAGTCAAAACCC

AAGTCCTTTCAGAGATGACGTGTCCACTCTGAGGAGGAACACACCATGTTCTATAAATAATCAATTGAGCCAAAGGGGAA

TTAGGCCCCTTCCTGAAGCCAGTTCCCTTCAATCAAAGAATATACCTTGCCTCAAAAGTGATCTTGAAGATGGAAACCAT

GTTTTGGAACAAACATTAGAAGGCAACCAGCTCAATGAAAGACCTGTTGATCTTACTCCTGAGCAGCTTGTTATGGATAC

ACCTGATGAAGGTCCCAAGCCAGAAGGAATTCCAAGTGAAGTGCAAGGAAATGGGGGCAAAAGGCAGCAACAAAGACCAT

CTACCATACTAAAGTTACCATGTTTTGACTCCAAATTAACAAAGTATTACACCTCCAGCACTGGGACTCCATCTAGTCTT

GGAAGGCAAGGCTCATTTGATGGGGATCCAATTATGCCTACTGAAATTCCTAACTCATTGGCTGAGTTAGCCACTGGGGC

ACAGTTTCAGAATATAAATGTAGACCCACTTAATGCAGATGATCACACTCCATTTGATCCTCTTCAAGTAGGGACCAATC

CACAGGACCAGGTTCAAGACTGCTTACTACTTCAGGCCTAGGAA--------------

>Enam-CertF1

---CCTTWCTTTGGATWTTTYGGATATCATGG------------------------------------------------

--------------------------------------------------------------------------------

--------------------------------------------------------------------------------

--------------------------------------------------------------------------------

--------------------------------------------------------------------------------

--------------------------------------------------------------------------------

--------------------------------------------------------------------------------

--------------------------------------------------------------------------------

--------------------------------------------------------------------------------

--------------------------------------------------------------------------------

--------------------------------------------------------------------------------

--------------------------------------------------------------------------------

--------------------------------------------------------------------------------

--------------------------------------------------------------------------------

--------------------------------------------------------------------------------

--------------------------------------------------------------------------------

--------------------------------------------------------------------------------

--------------------------------------------------------------------------------

--------------------------------------------------------------------------------

--------------------------------------------------------------------------------

--------------------------------------------------------------------------------

--------------------------------------------------------------------------------

--------------------------------------------------------------------------------

--------------------------------------------------------------------------------

--------------------------------------------------------------------------------

--------------------------------------------------------------------------------

--------------------------------------------------------------------------------

--------------------------------------------------------------------------------

--------------------------------------------------------------------------------

--------------------------------------------------------------------------------

--------------------------------------------------------------------------------

--------------------------------------------------------------------------------

--------------------------------------------------------------------------------

--------------------------------------------------------------------------------

--------------------------------------------------------------------------------

----------------------------------------------------------

>Enam-CertF2

-------------GATWTTTYGGATATCATGGATTTGGGGG---------------------------------------

--------------------------------------------------------------------------------

--------------------------------------------------------------------------------

--------------------------------------------------------------------------------

--------------------------------------------------------------------------------

--------------------------------------------------------------------------------

--------------------------------------------------------------------------------

--------------------------------------------------------------------------------

--------------------------------------------------------------------------------

--------------------------------------------------------------------------------

--------------------------------------------------------------------------------

--------------------------------------------------------------------------------

--------------------------------------------------------------------------------

--------------------------------------------------------------------------------

--------------------------------------------------------------------------------

--------------------------------------------------------------------------------

--------------------------------------------------------------------------------

--------------------------------------------------------------------------------

--------------------------------------------------------------------------------

--------------------------------------------------------------------------------

--------------------------------------------------------------------------------

--------------------------------------------------------------------------------

--------------------------------------------------------------------------------

--------------------------------------------------------------------------------

--------------------------------------------------------------------------------

--------------------------------------------------------------------------------

--------------------------------------------------------------------------------

--------------------------------------------------------------------------------

--------------------------------------------------------------------------------

--------------------------------------------------------------------------------

--------------------------------------------------------------------------------

--------------------------------------------------------------------------------

--------------------------------------------------------------------------------

--------------------------------------------------------------------------------

--------------------------------------------------------------------------------

----------------------------------------------------------

>Enam-CertIF1

--------------------------------------------------------------------------------

--------------------------------------------------------------------------------

--------------------------------------------------------------------------------

--------------------------------------------------------------------------------

--------------------------CAAAKATTCATGAAAATTAYCCAWATCCTAAC----------------------

--------------------------------------------------------------------------------

--------------------------------------------------------------------------------

--------------------------------------------------------------------------------

--------------------------------------------------------------------------------

--------------------------------------------------------------------------------

--------------------------------------------------------------------------------

--------------------------------------------------------------------------------

--------------------------------------------------------------------------------

--------------------------------------------------------------------------------

--------------------------------------------------------------------------------

--------------------------------------------------------------------------------

--------------------------------------------------------------------------------

--------------------------------------------------------------------------------

--------------------------------------------------------------------------------

--------------------------------------------------------------------------------

--------------------------------------------------------------------------------

--------------------------------------------------------------------------------

--------------------------------------------------------------------------------

--------------------------------------------------------------------------------

--------------------------------------------------------------------------------

--------------------------------------------------------------------------------

--------------------------------------------------------------------------------

--------------------------------------------------------------------------------

--------------------------------------------------------------------------------

--------------------------------------------------------------------------------

--------------------------------------------------------------------------------

--------------------------------------------------------------------------------

--------------------------------------------------------------------------------

--------------------------------------------------------------------------------

--------------------------------------------------------------------------------

----------------------------------------------------------

>Enam-CertIR1

--------------------------------------------------------------------------------

--------------------------------------------------------------------------------

--------------------------------------------------------------------------------

--------------------------------------------------------------------------------

--------------------------------------------------------------------------------

--------------------------------------------------------------------------------

--------------------------------------------------------------------------------

----------------------------GCAGGAAATYCAGCAAATHTCAGRAGAAAGCC--------------------

--------------------------------------------------------------------------------

--------------------------------------------------------------------------------

--------------------------------------------------------------------------------

--------------------------------------------------------------------------------

--------------------------------------------------------------------------------

--------------------------------------------------------------------------------

--------------------------------------------------------------------------------

--------------------------------------------------------------------------------

--------------------------------------------------------------------------------

--------------------------------------------------------------------------------

--------------------------------------------------------------------------------

--------------------------------------------------------------------------------

--------------------------------------------------------------------------------

--------------------------------------------------------------------------------

--------------------------------------------------------------------------------

--------------------------------------------------------------------------------

--------------------------------------------------------------------------------

--------------------------------------------------------------------------------

--------------------------------------------------------------------------------

--------------------------------------------------------------------------------

--------------------------------------------------------------------------------

--------------------------------------------------------------------------------

--------------------------------------------------------------------------------

--------------------------------------------------------------------------------

--------------------------------------------------------------------------------

--------------------------------------------------------------------------------

--------------------------------------------------------------------------------

----------------------------------------------------------

>Enam-CertR1

--------------------------------------------------------------------------------

--------------------------------------------------------------------------------

--------------------------------------------------------------------------------

--------------------------------------------------------------------------------

--------------------------------------------------------------------------------

--------------------------------------------------------------------------------

--------------------------------------------------------------------------------

--------------------------------------------------------------------------------

--------------------------------------------------------------------------------

--------------------------------------------------------------------------------

--------------------------------------------------------------------------------

--------------------------------------------------------------CAGAWTTTKCCCAAAGGG

ATTRTTTTAGARCC------------------------------------------------------------------

--------------------------------------------------------------------------------

--------------------------------------------------------------------------------

--------------------------------------------------------------------------------

--------------------------------------------------------------------------------

--------------------------------------------------------------------------------

--------------------------------------------------------------------------------

--------------------------------------------------------------------------------

--------------------------------------------------------------------------------

--------------------------------------------------------------------------------

--------------------------------------------------------------------------------

--------------------------------------------------------------------------------

--------------------------------------------------------------------------------

--------------------------------------------------------------------------------

--------------------------------------------------------------------------------

--------------------------------------------------------------------------------

--------------------------------------------------------------------------------

--------------------------------------------------------------------------------

--------------------------------------------------------------------------------

--------------------------------------------------------------------------------

--------------------------------------------------------------------------------

--------------------------------------------------------------------------------

--------------------------------------------------------------------------------

----------------------------------------------------------

>Enam-CertR2

--------------------------------------------------------------------------------

--------------------------------------------------------------------------------

--------------------------------------------------------------------------------

--------------------------------------------------------------------------------

--------------------------------------------------------------------------------

--------------------------------------------------------------------------------

--------------------------------------------------------------------------------

--------------------------------------------------------------------------------

--------------------------------------------------------------------------------

--------------------------------------------------------------------------------

--------------------------------------------------------------------------------

------------------------------------------------------------------------------GG

ATTRTTTTAGARCCAAGAAGAATYCC------------------------------------------------------

--------------------------------------------------------------------------------

--------------------------------------------------------------------------------

--------------------------------------------------------------------------------

--------------------------------------------------------------------------------

--------------------------------------------------------------------------------

--------------------------------------------------------------------------------

--------------------------------------------------------------------------------

--------------------------------------------------------------------------------

--------------------------------------------------------------------------------

--------------------------------------------------------------------------------

--------------------------------------------------------------------------------

--------------------------------------------------------------------------------

--------------------------------------------------------------------------------

--------------------------------------------------------------------------------

--------------------------------------------------------------------------------

--------------------------------------------------------------------------------

--------------------------------------------------------------------------------

--------------------------------------------------------------------------------

--------------------------------------------------------------------------------

--------------------------------------------------------------------------------

--------------------------------------------------------------------------------

--------------------------------------------------------------------------------

----------------------------------------------------------

>Enam-CertF3

--------------------------------------------------------------------------------

--------------------------------------------------------------------------------

--------------------------------------------------------------------------------

--------------------------------------------------------------------------------

--------------------------------------------------------------------------------

--------------------------------------------------------------------------------

--------------------------------------------------------------------------------

--------------------------------------------------------------------------------

--------------------------------------------------------------------------------

----------------------------------CAAGGGATCCAAVTGGCCCCTGGAGAAAC-----------------

--------------------------------------------------------------------------------

--------------------------------------------------------------------------------

--------------------------------------------------------------------------------

--------------------------------------------------------------------------------

--------------------------------------------------------------------------------

--------------------------------------------------------------------------------

--------------------------------------------------------------------------------

--------------------------------------------------------------------------------

--------------------------------------------------------------------------------

--------------------------------------------------------------------------------

--------------------------------------------------------------------------------

--------------------------------------------------------------------------------

--------------------------------------------------------------------------------

--------------------------------------------------------------------------------

--------------------------------------------------------------------------------

--------------------------------------------------------------------------------

--------------------------------------------------------------------------------

--------------------------------------------------------------------------------

--------------------------------------------------------------------------------

--------------------------------------------------------------------------------

--------------------------------------------------------------------------------

--------------------------------------------------------------------------------

--------------------------------------------------------------------------------

--------------------------------------------------------------------------------

--------------------------------------------------------------------------------

----------------------------------------------------------

>Enam-CertF4

--------------------------------------------------------------------------------

--------------------------------------------------------------------------------

--------------------------------------------------------------------------------

--------------------------------------------------------------------------------

--------------------------------------------------------------------------------

--------------------------------------------------------------------------------

--------------------------------------------------------------------------------

--------------------------------------------------------------------------------

--------------------------------------------------------------------------------

------------------------------------------------GGCCCCTGGAGAAACTCTSAAGACTATGG---

--------------------------------------------------------------------------------

--------------------------------------------------------------------------------

--------------------------------------------------------------------------------

--------------------------------------------------------------------------------

--------------------------------------------------------------------------------

--------------------------------------------------------------------------------

--------------------------------------------------------------------------------

--------------------------------------------------------------------------------

--------------------------------------------------------------------------------

--------------------------------------------------------------------------------

--------------------------------------------------------------------------------

--------------------------------------------------------------------------------

--------------------------------------------------------------------------------

--------------------------------------------------------------------------------

--------------------------------------------------------------------------------

--------------------------------------------------------------------------------

--------------------------------------------------------------------------------

--------------------------------------------------------------------------------

--------------------------------------------------------------------------------

--------------------------------------------------------------------------------

--------------------------------------------------------------------------------

--------------------------------------------------------------------------------

--------------------------------------------------------------------------------

--------------------------------------------------------------------------------

--------------------------------------------------------------------------------

----------------------------------------------------------

>Enam-CertIF2

--------------------------------------------------------------------------------

--------------------------------------------------------------------------------

--------------------------------------------------------------------------------

--------------------------------------------------------------------------------

--------------------------------------------------------------------------------

--------------------------------------------------------------------------------

--------------------------------------------------------------------------------

--------------------------------------------------------------------------------

--------------------------------------------------------------------------------

--------------------------------------------------------------------------------

--------------------------------------------------------------------------------

--------------------------------------------------------------------------------

--------------------------------------------------------------------------------

--------------------------------------------------GAAATACTTGGAAYCAMCAAGAAATCTCTC

C-------------------------------------------------------------------------------

--------------------------------------------------------------------------------

--------------------------------------------------------------------------------

--------------------------------------------------------------------------------

--------------------------------------------------------------------------------

--------------------------------------------------------------------------------

--------------------------------------------------------------------------------

--------------------------------------------------------------------------------

--------------------------------------------------------------------------------

--------------------------------------------------------------------------------

--------------------------------------------------------------------------------

--------------------------------------------------------------------------------

--------------------------------------------------------------------------------

--------------------------------------------------------------------------------

--------------------------------------------------------------------------------

--------------------------------------------------------------------------------

--------------------------------------------------------------------------------

--------------------------------------------------------------------------------

--------------------------------------------------------------------------------

--------------------------------------------------------------------------------

--------------------------------------------------------------------------------

----------------------------------------------------------

>Enam-CertIR2

--------------------------------------------------------------------------------

--------------------------------------------------------------------------------

--------------------------------------------------------------------------------

--------------------------------------------------------------------------------

--------------------------------------------------------------------------------

--------------------------------------------------------------------------------

--------------------------------------------------------------------------------

--------------------------------------------------------------------------------

--------------------------------------------------------------------------------

--------------------------------------------------------------------------------

--------------------------------------------------------------------------------

--------------------------------------------------------------------------------

--------------------------------------------------------------------------------

--------------------------------------------------------------------------------

--------------------------------------------------------------------------------

-------------CCTATGATCSYAGGGAAAAYTCACCATAC--------------------------------------

--------------------------------------------------------------------------------

--------------------------------------------------------------------------------

--------------------------------------------------------------------------------

--------------------------------------------------------------------------------

--------------------------------------------------------------------------------

--------------------------------------------------------------------------------

--------------------------------------------------------------------------------

--------------------------------------------------------------------------------

--------------------------------------------------------------------------------

--------------------------------------------------------------------------------

--------------------------------------------------------------------------------

--------------------------------------------------------------------------------

--------------------------------------------------------------------------------

--------------------------------------------------------------------------------

--------------------------------------------------------------------------------

--------------------------------------------------------------------------------

--------------------------------------------------------------------------------

--------------------------------------------------------------------------------

--------------------------------------------------------------------------------

----------------------------------------------------------

>Enam-CertR3

--------------------------------------------------------------------------------

--------------------------------------------------------------------------------

--------------------------------------------------------------------------------

--------------------------------------------------------------------------------

--------------------------------------------------------------------------------

--------------------------------------------------------------------------------

--------------------------------------------------------------------------------

--------------------------------------------------------------------------------

--------------------------------------------------------------------------------

--------------------------------------------------------------------------------

--------------------------------------------------------------------------------

--------------------------------------------------------------------------------

--------------------------------------------------------------------------------

--------------------------------------------------------------------------------

--------------------------------------------------------------------------------

--------------------------------------------------------------------------------

--------------------------------------------------------------------------------

--------------------------------------------------------------------------------

--------------------------------------------------------------------------------

-------CCCAGRGAGGATTTTCYTTAYGGTGAATTTTACC---------------------------------------

--------------------------------------------------------------------------------

--------------------------------------------------------------------------------

--------------------------------------------------------------------------------

--------------------------------------------------------------------------------

--------------------------------------------------------------------------------

--------------------------------------------------------------------------------

--------------------------------------------------------------------------------

--------------------------------------------------------------------------------

--------------------------------------------------------------------------------

--------------------------------------------------------------------------------

--------------------------------------------------------------------------------

--------------------------------------------------------------------------------

--------------------------------------------------------------------------------

--------------------------------------------------------------------------------

--------------------------------------------------------------------------------

----------------------------------------------------------

>Enam-CertR4

--------------------------------------------------------------------------------

--------------------------------------------------------------------------------

--------------------------------------------------------------------------------

--------------------------------------------------------------------------------

--------------------------------------------------------------------------------

--------------------------------------------------------------------------------

--------------------------------------------------------------------------------

--------------------------------------------------------------------------------

--------------------------------------------------------------------------------

--------------------------------------------------------------------------------

--------------------------------------------------------------------------------

--------------------------------------------------------------------------------

--------------------------------------------------------------------------------

--------------------------------------------------------------------------------

--------------------------------------------------------------------------------

--------------------------------------------------------------------------------

--------------------------------------------------------------------------------

--------------------------------------------------------------------------------

--------------------------------------------------------------------------------

----------------------CYTTAYGGTGAATTTTACCCCTGGARCCCAG---------------------------

--------------------------------------------------------------------------------

--------------------------------------------------------------------------------

--------------------------------------------------------------------------------

--------------------------------------------------------------------------------

--------------------------------------------------------------------------------

--------------------------------------------------------------------------------

--------------------------------------------------------------------------------

--------------------------------------------------------------------------------

--------------------------------------------------------------------------------

--------------------------------------------------------------------------------

--------------------------------------------------------------------------------

--------------------------------------------------------------------------------

--------------------------------------------------------------------------------

--------------------------------------------------------------------------------

--------------------------------------------------------------------------------

----------------------------------------------------------

>Enam-CertF5

--------------------------------------------------------------------------------

--------------------------------------------------------------------------------

--------------------------------------------------------------------------------

--------------------------------------------------------------------------------

--------------------------------------------------------------------------------

--------------------------------------------------------------------------------

--------------------------------------------------------------------------------

--------------------------------------------------------------------------------

--------------------------------------------------------------------------------

--------------------------------------------------------------------------------

--------------------------------------------------------------------------------

--------------------------------------------------------------------------------

--------------------------------------------------------------------------------

--------------------------------------------------------------------------------

--------------------------------------------------------------------------------

--------------------------------------------------------------------------------

--------------------------------------------------------------------------------

-----------GATCCAACTGGAGATGAAVMTTTCCCAGGAC--------------------------------------

--------------------------------------------------------------------------------

--------------------------------------------------------------------------------

--------------------------------------------------------------------------------

--------------------------------------------------------------------------------

--------------------------------------------------------------------------------

--------------------------------------------------------------------------------

--------------------------------------------------------------------------------

--------------------------------------------------------------------------------

--------------------------------------------------------------------------------

--------------------------------------------------------------------------------

--------------------------------------------------------------------------------

--------------------------------------------------------------------------------

--------------------------------------------------------------------------------

--------------------------------------------------------------------------------

--------------------------------------------------------------------------------

--------------------------------------------------------------------------------

--------------------------------------------------------------------------------

----------------------------------------------------------

>Enam-CertF6

--------------------------------------------------------------------------------

--------------------------------------------------------------------------------

--------------------------------------------------------------------------------

--------------------------------------------------------------------------------

--------------------------------------------------------------------------------

--------------------------------------------------------------------------------

--------------------------------------------------------------------------------

--------------------------------------------------------------------------------

--------------------------------------------------------------------------------

--------------------------------------------------------------------------------

--------------------------------------------------------------------------------

--------------------------------------------------------------------------------

--------------------------------------------------------------------------------

--------------------------------------------------------------------------------

--------------------------------------------------------------------------------

--------------------------------------------------------------------------------

--------------------------------------------------------------------------------

--------------------GGAGATGAAVMTTTCCCAGGACAAASKAGATGG---------------------------

--------------------------------------------------------------------------------

--------------------------------------------------------------------------------

--------------------------------------------------------------------------------

--------------------------------------------------------------------------------

--------------------------------------------------------------------------------

--------------------------------------------------------------------------------

--------------------------------------------------------------------------------

--------------------------------------------------------------------------------

--------------------------------------------------------------------------------

--------------------------------------------------------------------------------

--------------------------------------------------------------------------------

--------------------------------------------------------------------------------

--------------------------------------------------------------------------------

--------------------------------------------------------------------------------

--------------------------------------------------------------------------------

--------------------------------------------------------------------------------

--------------------------------------------------------------------------------

----------------------------------------------------------

>Enam-CertIF3

--------------------------------------------------------------------------------

--------------------------------------------------------------------------------

--------------------------------------------------------------------------------

--------------------------------------------------------------------------------

--------------------------------------------------------------------------------

--------------------------------------------------------------------------------

--------------------------------------------------------------------------------

--------------------------------------------------------------------------------

--------------------------------------------------------------------------------

--------------------------------------------------------------------------------

--------------------------------------------------------------------------------

--------------------------------------------------------------------------------

--------------------------------------------------------------------------------

--------------------------------------------------------------------------------

--------------------------------------------------------------------------------

--------------------------------------------------------------------------------

--------------------------------------------------------------------------------

--------------------------------------------------------------------------------

--------------------------------------------------------------------------------

--------------------------------------------------------------------------------

--------------------------------------------------------------------------------

--------------------------------------------------------------------------------

--------------------------------------------------------------------------------

---CTGGKCTTCAGAAAAATCCAAYATGGCMTGAAG--------------------------------------------

--------------------------------------------------------------------------------

--------------------------------------------------------------------------------

--------------------------------------------------------------------------------

--------------------------------------------------------------------------------

--------------------------------------------------------------------------------

--------------------------------------------------------------------------------

--------------------------------------------------------------------------------

--------------------------------------------------------------------------------

--------------------------------------------------------------------------------

--------------------------------------------------------------------------------

--------------------------------------------------------------------------------

----------------------------------------------------------

>Enam-CertIR3

--------------------------------------------------------------------------------

--------------------------------------------------------------------------------

--------------------------------------------------------------------------------

--------------------------------------------------------------------------------

--------------------------------------------------------------------------------

--------------------------------------------------------------------------------

--------------------------------------------------------------------------------

--------------------------------------------------------------------------------

--------------------------------------------------------------------------------

--------------------------------------------------------------------------------

--------------------------------------------------------------------------------

--------------------------------------------------------------------------------

--------------------------------------------------------------------------------

--------------------------------------------------------------------------------

--------------------------------------------------------------------------------

--------------------------------------------------------------------------------

--------------------------------------------------------------------------------

--------------------------------------------------------------------------------

--------------------------------------------------------------------------------

--------------------------------------------------------------------------------

--------------------------------------------------------------------------------

--------------------------------------------------------------------------------

--------------------------------------------------------------------------------

-----------------------------------------------------GGYATGCARAYCACTAGGTTAAATTCA

CCAG----------------------------------------------------------------------------

--------------------------------------------------------------------------------

--------------------------------------------------------------------------------

--------------------------------------------------------------------------------

--------------------------------------------------------------------------------

--------------------------------------------------------------------------------

--------------------------------------------------------------------------------

--------------------------------------------------------------------------------

--------------------------------------------------------------------------------

--------------------------------------------------------------------------------

--------------------------------------------------------------------------------

----------------------------------------------------------

>Enam-CertR5

--------------------------------------------------------------------------------

--------------------------------------------------------------------------------

--------------------------------------------------------------------------------

--------------------------------------------------------------------------------

--------------------------------------------------------------------------------

--------------------------------------------------------------------------------

--------------------------------------------------------------------------------

--------------------------------------------------------------------------------

--------------------------------------------------------------------------------

--------------------------------------------------------------------------------

--------------------------------------------------------------------------------

--------------------------------------------------------------------------------

--------------------------------------------------------------------------------

--------------------------------------------------------------------------------

--------------------------------------------------------------------------------

--------------------------------------------------------------------------------

--------------------------------------------------------------------------------

--------------------------------------------------------------------------------

--------------------------------------------------------------------------------

--------------------------------------------------------------------------------

--------------------------------------------------------------------------------

--------------------------------------------------------------------------------

--------------------------------------------------------------------------------

--------------------------------------------------------------------------------

--------------------------------------------------------------------------------

--------------------------------------------------------------------------------

--------------------------------------------------------------------------------

-----------------------------------------CAGCTCAGARAAAAGACTKCCTGGAGAAAG---------

--------------------------------------------------------------------------------

--------------------------------------------------------------------------------

--------------------------------------------------------------------------------

--------------------------------------------------------------------------------

--------------------------------------------------------------------------------

--------------------------------------------------------------------------------

--------------------------------------------------------------------------------

----------------------------------------------------------

>Enam-CertR6

--------------------------------------------------------------------------------

--------------------------------------------------------------------------------

--------------------------------------------------------------------------------

--------------------------------------------------------------------------------

--------------------------------------------------------------------------------

--------------------------------------------------------------------------------

--------------------------------------------------------------------------------

--------------------------------------------------------------------------------

--------------------------------------------------------------------------------

--------------------------------------------------------------------------------

--------------------------------------------------------------------------------

--------------------------------------------------------------------------------

--------------------------------------------------------------------------------

--------------------------------------------------------------------------------

--------------------------------------------------------------------------------

--------------------------------------------------------------------------------

--------------------------------------------------------------------------------

--------------------------------------------------------------------------------

--------------------------------------------------------------------------------

--------------------------------------------------------------------------------

--------------------------------------------------------------------------------

--------------------------------------------------------------------------------

--------------------------------------------------------------------------------

--------------------------------------------------------------------------------

--------------------------------------------------------------------------------

--------------------------------------------------------------------------------

--------------------------------------------------------------------------------

-------------------------------------------------------GACTKCCTGGAGAAAGTCAAAACCC

AAGTCC--------------------------------------------------------------------------

--------------------------------------------------------------------------------

--------------------------------------------------------------------------------

--------------------------------------------------------------------------------

--------------------------------------------------------------------------------

--------------------------------------------------------------------------------

--------------------------------------------------------------------------------

----------------------------------------------------------

>Enam-CertF7

--------------------------------------------------------------------------------

--------------------------------------------------------------------------------

--------------------------------------------------------------------------------

--------------------------------------------------------------------------------

--------------------------------------------------------------------------------

--------------------------------------------------------------------------------

--------------------------------------------------------------------------------

--------------------------------------------------------------------------------

--------------------------------------------------------------------------------

--------------------------------------------------------------------------------

--------------------------------------------------------------------------------

--------------------------------------------------------------------------------

--------------------------------------------------------------------------------

--------------------------------------------------------------------------------

--------------------------------------------------------------------------------

--------------------------------------------------------------------------------

--------------------------------------------------------------------------------

--------------------------------------------------------------------------------

--------------------------------------------------------------------------------

--------------------------------------------------------------------------------

--------------------------------------------------------------------------------

--------------------------------------------------------------------------------

--------------------------------------------------------------------------------

--------------------CCAAYATGGCMTGAAGGTGAGAATTTG---------------------------------

--------------------------------------------------------------------------------

--------------------------------------------------------------------------------

--------------------------------------------------------------------------------

--------------------------------------------------------------------------------

--------------------------------------------------------------------------------

--------------------------------------------------------------------------------

--------------------------------------------------------------------------------

--------------------------------------------------------------------------------

--------------------------------------------------------------------------------

--------------------------------------------------------------------------------

--------------------------------------------------------------------------------

----------------------------------------------------------

>Enam-CertF8

--------------------------------------------------------------------------------

--------------------------------------------------------------------------------

--------------------------------------------------------------------------------

--------------------------------------------------------------------------------

--------------------------------------------------------------------------------

--------------------------------------------------------------------------------

--------------------------------------------------------------------------------

--------------------------------------------------------------------------------

--------------------------------------------------------------------------------

--------------------------------------------------------------------------------

--------------------------------------------------------------------------------

--------------------------------------------------------------------------------

--------------------------------------------------------------------------------

--------------------------------------------------------------------------------

--------------------------------------------------------------------------------

--------------------------------------------------------------------------------

--------------------------------------------------------------------------------

--------------------------------------------------------------------------------

--------------------------------------------------------------------------------

--------------------------------------------------------------------------------

--------------------------------------------------------------------------------

--------------------------------------------------------------------------------

--------------------------------------------------------------------------------

--------------------------------GAAGGTGAGAATTTGAATTATGGYATGC--------------------

--------------------------------------------------------------------------------

--------------------------------------------------------------------------------

--------------------------------------------------------------------------------

--------------------------------------------------------------------------------

--------------------------------------------------------------------------------

--------------------------------------------------------------------------------

--------------------------------------------------------------------------------

--------------------------------------------------------------------------------

--------------------------------------------------------------------------------

--------------------------------------------------------------------------------

--------------------------------------------------------------------------------

----------------------------------------------------------

>Enam-CertIF4

--------------------------------------------------------------------------------

--------------------------------------------------------------------------------

--------------------------------------------------------------------------------

--------------------------------------------------------------------------------

--------------------------------------------------------------------------------

--------------------------------------------------------------------------------

--------------------------------------------------------------------------------

--------------------------------------------------------------------------------

--------------------------------------------------------------------------------

--------------------------------------------------------------------------------

--------------------------------------------------------------------------------

--------------------------------------------------------------------------------

--------------------------------------------------------------------------------

--------------------------------------------------------------------------------

--------------------------------------------------------------------------------

--------------------------------------------------------------------------------

--------------------------------------------------------------------------------

--------------------------------------------------------------------------------

--------------------------------------------------------------------------------

--------------------------------------------------------------------------------

--------------------------------------------------------------------------------

--------------------------------------------------------------------------------

--------------------------------------------------------------------------------

--------------------------------------------------------------------------------

--------------------------------------------------------------------------------

--------------------------------------------------------------------------------

--------------------------------------------------------------------------------

--------------------------------------------------------------------------------

---------------------GTCCMCTCTGAGGAGGAACAYACCAYGTTC-----------------------------

--------------------------------------------------------------------------------

--------------------------------------------------------------------------------

--------------------------------------------------------------------------------

--------------------------------------------------------------------------------

--------------------------------------------------------------------------------

--------------------------------------------------------------------------------

----------------------------------------------------------

>Enam-CertIR4

--------------------------------------------------------------------------------

--------------------------------------------------------------------------------

--------------------------------------------------------------------------------

--------------------------------------------------------------------------------

--------------------------------------------------------------------------------

--------------------------------------------------------------------------------

--------------------------------------------------------------------------------

--------------------------------------------------------------------------------

--------------------------------------------------------------------------------

--------------------------------------------------------------------------------

--------------------------------------------------------------------------------

--------------------------------------------------------------------------------

--------------------------------------------------------------------------------

--------------------------------------------------------------------------------

--------------------------------------------------------------------------------

--------------------------------------------------------------------------------

--------------------------------------------------------------------------------

--------------------------------------------------------------------------------

--------------------------------------------------------------------------------

--------------------------------------------------------------------------------

--------------------------------------------------------------------------------

--------------------------------------------------------------------------------

--------------------------------------------------------------------------------

--------------------------------------------------------------------------------

--------------------------------------------------------------------------------

--------------------------------------------------------------------------------

--------------------------------------------------------------------------------

--------------------------------------------------------------------------------

--------------------------------------------------------------------------------

--------------GAARCCAGTTCCCTTCAAYCAAAGAATAYACCTTG-------------------------------

--------------------------------------------------------------------------------

--------------------------------------------------------------------------------

--------------------------------------------------------------------------------

--------------------------------------------------------------------------------

--------------------------------------------------------------------------------

----------------------------------------------------------

>Enam-CertR7

--------------------------------------------------------------------------------

--------------------------------------------------------------------------------

--------------------------------------------------------------------------------

--------------------------------------------------------------------------------

--------------------------------------------------------------------------------

--------------------------------------------------------------------------------

--------------------------------------------------------------------------------

--------------------------------------------------------------------------------

--------------------------------------------------------------------------------

--------------------------------------------------------------------------------

--------------------------------------------------------------------------------

--------------------------------------------------------------------------------

--------------------------------------------------------------------------------

--------------------------------------------------------------------------------

--------------------------------------------------------------------------------

--------------------------------------------------------------------------------

--------------------------------------------------------------------------------

--------------------------------------------------------------------------------

--------------------------------------------------------------------------------

--------------------------------------------------------------------------------

--------------------------------------------------------------------------------

--------------------------------------------------------------------------------

--------------------------------------------------------------------------------

--------------------------------------------------------------------------------

--------------------------------------------------------------------------------

--------------------------------------------------------------------------------

--------------------------------------------------------------------------------

--------------------------------------------------------------------------------

--------------------------------------------------------------------------------

--------------------------------------------------------------------------------

--------------------------------------------------------------------------------

--------------------------------------------------------------------------------

--------------------------------------------------------------------------------

--------------------------------------------------------------------------------

-----------------------------------------------------------------------GGACCRATC

CASARGACCAGGTKCAAGAC--------------------------------------

>Enam-CertR8

--------------------------------------------------------------------------------

--------------------------------------------------------------------------------

--------------------------------------------------------------------------------

--------------------------------------------------------------------------------

--------------------------------------------------------------------------------

--------------------------------------------------------------------------------

--------------------------------------------------------------------------------

--------------------------------------------------------------------------------

--------------------------------------------------------------------------------

--------------------------------------------------------------------------------

--------------------------------------------------------------------------------

--------------------------------------------------------------------------------

--------------------------------------------------------------------------------

--------------------------------------------------------------------------------

--------------------------------------------------------------------------------

--------------------------------------------------------------------------------

--------------------------------------------------------------------------------

--------------------------------------------------------------------------------

--------------------------------------------------------------------------------

--------------------------------------------------------------------------------

--------------------------------------------------------------------------------

--------------------------------------------------------------------------------

--------------------------------------------------------------------------------

--------------------------------------------------------------------------------

--------------------------------------------------------------------------------

--------------------------------------------------------------------------------

--------------------------------------------------------------------------------

--------------------------------------------------------------------------------

--------------------------------------------------------------------------------

--------------------------------------------------------------------------------

--------------------------------------------------------------------------------

--------------------------------------------------------------------------------

--------------------------------------------------------------------------------

--------------------------------------------------------------------------------

--------------------------------------------------------------------------------

-----GACCAGGTKCAAGACTGCYTACTACTTCAGGCC--------------------

**Chiroptera Primers**

>Myotis

AATCCTTACTTTGGGTATTTCGGATATCAGGGATTTGGGGGTCGTCCTCCCTATTATTCAGAAGAAATGTTTGAACAAGA

TTTTGAAAAACCCAAAGAAAAAGACCCTCCTAAAACGGAAAGTCCAGCCACCGAGCCCTCGTCAAATTCAACTGTTCCTG

AGACTAATTCTACCCAACCAAATGCACCCAATCCTGGAGGGCGTCCGGGTGGAAATGACACCAGCCCAACAGGAAACAGT

GGCCATGGGCCCAACACTGGGGGCAACCCTACAGCTCAAAATGGGGTCAATCCACACCCTGCAGTTAATGTTTCAGGCCA

GGGGCCACCAAGAAGTCAAATCCCATGGGGCTCAGGTCAGCCAAATATTCGTGAAAATTACCCAAATCCTAACTTTCCAA

GTTTTCCTGTGGGAAGACAATGGCGTCCCACTGGTACCACCGTGGGACACAGACAGAATGGGCCTTTTTACCGAAATCCA

GTCCAAAGGGGTCCTCGGTGGAACTCCTATGCTTGGGAAGGCAAGCAAGCTGTTCGTCCAGGAAATCCAATTTATCACAG

ACCGTATGCTTCCACTGCAAGAGGCAATTCTCCCAATCTTGCAGGAAATCCAGCAAATTTCAGAAGAAAACCTCAGGGGC

CAAATAAACACCCTATGGGAACCAATGTTGCCCCTTTGGGTCCCAAACATGGTACTGTTGGTCGCAATGAAAAAATTCAA

AATCCAAGAGAAAAGCCATTAGGTCAAAAAGAACGAGTAGTCATTCCTACAAGAGATCCAACTGGCCCCTGGAGAAACTC

TCAAGATAATGAAGCTAATAAATCAAACTATAAACTGCCTCCTCCCGAGGGTAACAAGCAACTCCCAAATTTTAATTCTA

TTGATCAGCGTGAAAATTCTTATTACCCAAGAGGAGATTCCAGAAGAGCCCCAAATTCTGATGGACAAGCCCAGAGCCAG

AATTTGCCCAAAGGGATTATTTTAGAGCCAAGAAGGATCCCATATGAATCAGAAACTAATCAACCAGAATTGAAGCACAG

TACATATCAGCCTGCATACCCTGAGGACATCCCTTCCCCTGCAGGAGAACGTTTTCCTGCTGGAAGAAATACTTGGAACC

ACCAAGACATCTCTCCACCTTTTAAGGAAGATCCCAACAGGCAGGAAGAACACTTACCTCATCCTTCCCATGGCTCTAGG

GCAGATGTGTACTACCCTGACTATAACCCCTATGATCCCAGGGAAAACTCACCATACATTAGAAGCAATACATGGGATGA

AAGAGATGATTCTCCCAATAATGCGGGACTACCAGAAGATCCGCTATACCCCATGAATACTCCAGACCCAAAAGAGACAG

TACCTTATAATGAAGAGGACCCAATTGACCCAACTGGAGACGAACCTTTTCCAGGACAAAGTAGATGGGATGTGGAGGAG

TCGCGCTTTAAAGAAGGTGCCCCAGTTAGGCACTACAAAGATGAACAGTATACCTCAAGTCGACCAAAGGAGTACCTTCC

CTATTCCTTAGATAATCCATCGAAACCCAGGGAGGATTTTCCTTATGGTGAATTTTACCCCTGGAACGCAGATGAGAATT

TCCCATCATATAATACAATTCCCACTGTACCACCACCTGTGGAGAACAGGGGCTATTATGCCAACAGACCTGTTGGACAA

GAAGAAAACTCTCTGTTTCCTTCTTGGAACTCCTGGGACCATAGGATAGAAACCCAAGGGCAGAAAGAAAGAAGACCATA

CTTTAACAGAAATTTCTGGGATCGGCCAACCAATTTACACAAAGCCCCTGCTAGCCCACCACACCAGAAAGAGAACCCGC

ACTATTCCAGTAACTCCCCAGCTGGGCTTCAGAAAAATCCAATATGGCGTGAAGGTGAGAATTTGAATTATGGAATGCAG

AATACTAGGTTAAATTCACCAGACAATGAACACTTGGCTTTCCAGGACTTAATTCCTCCAAGTTATTCAGCAGGTCAAAA

AGAAGCACATTTATTTCACCAAAGACAAAGAAGCCCTTGCTGTGCTGGTGGCTCTGTTGGACACAAGGACAATCCACTTG

CTCTACAAGACTACACTCCATCCTTTGGTCTTGCACCAGGGGAGAACCAAGACACCAGTCCCCTGTATACAAAAGATAGC

CACACTAAGCATGCAAGACATACCATCTCCCCGACGAGCAGCCTACCTAGCCAAAGAAACAGCTCAGAGAAAAGACAGCC

TGGAGAAAGTCAAAACCCAAGTCCTTTTAGAGATGATGTGTCCACTTTGAGGAGGAACATACCGTGTTCTATAAAGAATC

AACTGGGCCAAAGGGGAATTATGCCCTTTTCTGAAGCCAGTTCCCTTCAATCAAAGAATACACCTTGTCTCAAAAATGAC

CTTGGAGGAGATGGAAACAATGCTTTGGAACCAATATTTGAAGGCAACCAGCTTAATGAAAGGACTGTTGACCTTACTCC

CGAGCAGCTTGTTATCAGCACACCTGATGAAGGCCCCAAGCCAGAAGAAATCCAAAGTGAAGTGCAAGGAAATGAGGGTG

AAAGGCAGCAACAAAGACCATCTAGCATTCTACAGTTACCATGCTTTGGCTCCAAATTAGCAAAGTATCACTCCTCCAGC

CCTGGAACTCCATCTAGCATTGGAAGGCAAAGCCCATTCAATGGCGATTCAATAATGCCTACTGAAATTCCTGACTCATT

GGCTGGGTTAGCTACTGGGACCCAGTTTCAGAGTATAATTGTAGACCCATATAATGATGATGAACACATTCCATTTGATT

CCCTTCAAATAGGGACCAAACC------------------------------------

>Enam-ChiropF1

---CCTTACTTTGGRTATTTYGGATATCAKGG------------------------------------------------

--------------------------------------------------------------------------------

--------------------------------------------------------------------------------

--------------------------------------------------------------------------------

--------------------------------------------------------------------------------

--------------------------------------------------------------------------------

--------------------------------------------------------------------------------

--------------------------------------------------------------------------------

--------------------------------------------------------------------------------

--------------------------------------------------------------------------------

--------------------------------------------------------------------------------

--------------------------------------------------------------------------------

--------------------------------------------------------------------------------

--------------------------------------------------------------------------------

--------------------------------------------------------------------------------

--------------------------------------------------------------------------------

--------------------------------------------------------------------------------

--------------------------------------------------------------------------------

--------------------------------------------------------------------------------

--------------------------------------------------------------------------------

--------------------------------------------------------------------------------

--------------------------------------------------------------------------------

--------------------------------------------------------------------------------

--------------------------------------------------------------------------------

--------------------------------------------------------------------------------

--------------------------------------------------------------------------------

--------------------------------------------------------------------------------

--------------------------------------------------------------------------------

--------------------------------------------------------------------------------

--------------------------------------------------------------------------------

--------------------------------------------------------------------------------

--------------------------------------------------------------------------------

--------------------------------------------------------------------------------

--------------------------------------------------------------------------------

--------------------------------------------------------------------------------

----------------------------------------------------------

>Enam-ChiropF2

------------GGRTATTTYGGATATCAKGGATTTGGGGG---------------------------------------

--------------------------------------------------------------------------------

--------------------------------------------------------------------------------

--------------------------------------------------------------------------------

--------------------------------------------------------------------------------

--------------------------------------------------------------------------------

--------------------------------------------------------------------------------

--------------------------------------------------------------------------------

--------------------------------------------------------------------------------

--------------------------------------------------------------------------------

--------------------------------------------------------------------------------

--------------------------------------------------------------------------------

--------------------------------------------------------------------------------

--------------------------------------------------------------------------------

--------------------------------------------------------------------------------

--------------------------------------------------------------------------------

--------------------------------------------------------------------------------

--------------------------------------------------------------------------------

--------------------------------------------------------------------------------

--------------------------------------------------------------------------------

--------------------------------------------------------------------------------

--------------------------------------------------------------------------------

--------------------------------------------------------------------------------

--------------------------------------------------------------------------------

--------------------------------------------------------------------------------

--------------------------------------------------------------------------------

--------------------------------------------------------------------------------

--------------------------------------------------------------------------------

--------------------------------------------------------------------------------

--------------------------------------------------------------------------------

--------------------------------------------------------------------------------

--------------------------------------------------------------------------------

--------------------------------------------------------------------------------

--------------------------------------------------------------------------------

--------------------------------------------------------------------------------

----------------------------------------------------------

>Enam-ChirIF1

--------------------------------------------------------------------------------

--------------------------------------------------------------------------------

--------------------------------------------------------------------------------

--------------------------------------------------------------------------------

-------------------------------------CAGCCAAATATTCGTGAAAATTACCCAAATCC-----------

--------------------------------------------------------------------------------

--------------------------------------------------------------------------------

--------------------------------------------------------------------------------

--------------------------------------------------------------------------------

--------------------------------------------------------------------------------

--------------------------------------------------------------------------------

--------------------------------------------------------------------------------

--------------------------------------------------------------------------------

--------------------------------------------------------------------------------

--------------------------------------------------------------------------------

--------------------------------------------------------------------------------

--------------------------------------------------------------------------------

--------------------------------------------------------------------------------

--------------------------------------------------------------------------------

--------------------------------------------------------------------------------

--------------------------------------------------------------------------------

--------------------------------------------------------------------------------

--------------------------------------------------------------------------------

--------------------------------------------------------------------------------

--------------------------------------------------------------------------------

--------------------------------------------------------------------------------

--------------------------------------------------------------------------------

--------------------------------------------------------------------------------

--------------------------------------------------------------------------------

--------------------------------------------------------------------------------

--------------------------------------------------------------------------------

--------------------------------------------------------------------------------

--------------------------------------------------------------------------------

--------------------------------------------------------------------------------

--------------------------------------------------------------------------------

----------------------------------------------------------

>Enam-ChirIR1

--------------------------------------------------------------------------------

--------------------------------------------------------------------------------

--------------------------------------------------------------------------------

--------------------------------------------------------------------------------

--------------------------------------------------------------------------------

--------------------------------------------------------------------------------

--------------------------------------------------------------------------------

----------------------GGCAATTCTCCCARTCWTGCAGGAAATCCAGC--------------------------

--------------------------------------------------------------------------------

--------------------------------------------------------------------------------

--------------------------------------------------------------------------------

--------------------------------------------------------------------------------

--------------------------------------------------------------------------------

--------------------------------------------------------------------------------

--------------------------------------------------------------------------------

--------------------------------------------------------------------------------

--------------------------------------------------------------------------------

--------------------------------------------------------------------------------

--------------------------------------------------------------------------------

--------------------------------------------------------------------------------

--------------------------------------------------------------------------------

--------------------------------------------------------------------------------

--------------------------------------------------------------------------------

--------------------------------------------------------------------------------

--------------------------------------------------------------------------------

--------------------------------------------------------------------------------

--------------------------------------------------------------------------------

--------------------------------------------------------------------------------

--------------------------------------------------------------------------------

--------------------------------------------------------------------------------

--------------------------------------------------------------------------------

--------------------------------------------------------------------------------

--------------------------------------------------------------------------------

--------------------------------------------------------------------------------

--------------------------------------------------------------------------------

----------------------------------------------------------

>Enam-ChiropR1

--------------------------------------------------------------------------------

--------------------------------------------------------------------------------

--------------------------------------------------------------------------------

--------------------------------------------------------------------------------

--------------------------------------------------------------------------------

--------------------------------------------------------------------------------

--------------------------------------------------------------------------------

--------------------------------------------------------------------------------

--------------------------------------------------------------------------------

--------------------------------------------------------------------------------

--------------------------------------------------------------------------------

-----------------------------------------------------------------------------CAG

AATTTGCCCAAAGGGATTATTKTAGAGCC---------------------------------------------------

--------------------------------------------------------------------------------

--------------------------------------------------------------------------------

--------------------------------------------------------------------------------

--------------------------------------------------------------------------------

--------------------------------------------------------------------------------

--------------------------------------------------------------------------------

--------------------------------------------------------------------------------

--------------------------------------------------------------------------------

--------------------------------------------------------------------------------

--------------------------------------------------------------------------------

--------------------------------------------------------------------------------

--------------------------------------------------------------------------------

--------------------------------------------------------------------------------

--------------------------------------------------------------------------------

--------------------------------------------------------------------------------

--------------------------------------------------------------------------------

--------------------------------------------------------------------------------

--------------------------------------------------------------------------------

--------------------------------------------------------------------------------

--------------------------------------------------------------------------------

--------------------------------------------------------------------------------

--------------------------------------------------------------------------------

----------------------------------------------------------

>Enam-ChiropR2

--------------------------------------------------------------------------------

--------------------------------------------------------------------------------

--------------------------------------------------------------------------------

--------------------------------------------------------------------------------

--------------------------------------------------------------------------------

--------------------------------------------------------------------------------

--------------------------------------------------------------------------------

--------------------------------------------------------------------------------

--------------------------------------------------------------------------------

--------------------------------------------------------------------------------

--------------------------------------------------------------------------------

--------------------------------------------------------------------------------

-------------GGATTATTKTAGAGCCAAGAAGGATCCC---------------------------------------

--------------------------------------------------------------------------------

--------------------------------------------------------------------------------

--------------------------------------------------------------------------------

--------------------------------------------------------------------------------

--------------------------------------------------------------------------------

--------------------------------------------------------------------------------

--------------------------------------------------------------------------------

--------------------------------------------------------------------------------

--------------------------------------------------------------------------------

--------------------------------------------------------------------------------

--------------------------------------------------------------------------------

--------------------------------------------------------------------------------

--------------------------------------------------------------------------------

--------------------------------------------------------------------------------

--------------------------------------------------------------------------------

--------------------------------------------------------------------------------

--------------------------------------------------------------------------------

--------------------------------------------------------------------------------

--------------------------------------------------------------------------------

--------------------------------------------------------------------------------

--------------------------------------------------------------------------------

--------------------------------------------------------------------------------

----------------------------------------------------------

>Enam-ChiropF3

--------------------------------------------------------------------------------

--------------------------------------------------------------------------------

--------------------------------------------------------------------------------

--------------------------------------------------------------------------------

--------------------------------------------------------------------------------

--------------------------------------------------------------------------------

--------------------------------------------------------------------------------

--------------------------------------------------------------------------------

--------------------------------------------------------------------------------

---------------------------------------GTCATTCCTACAAGAGATCCAACTGGCC-------------

--------------------------------------------------------------------------------

--------------------------------------------------------------------------------

--------------------------------------------------------------------------------

--------------------------------------------------------------------------------

--------------------------------------------------------------------------------

--------------------------------------------------------------------------------

--------------------------------------------------------------------------------

--------------------------------------------------------------------------------

--------------------------------------------------------------------------------

--------------------------------------------------------------------------------

--------------------------------------------------------------------------------

--------------------------------------------------------------------------------

--------------------------------------------------------------------------------

--------------------------------------------------------------------------------

--------------------------------------------------------------------------------

--------------------------------------------------------------------------------

--------------------------------------------------------------------------------

--------------------------------------------------------------------------------

--------------------------------------------------------------------------------

--------------------------------------------------------------------------------

--------------------------------------------------------------------------------

--------------------------------------------------------------------------------

--------------------------------------------------------------------------------

--------------------------------------------------------------------------------

--------------------------------------------------------------------------------

----------------------------------------------------------

>Enam-ChiropF4

--------------------------------------------------------------------------------

--------------------------------------------------------------------------------

--------------------------------------------------------------------------------

--------------------------------------------------------------------------------

--------------------------------------------------------------------------------

--------------------------------------------------------------------------------

--------------------------------------------------------------------------------

--------------------------------------------------------------------------------

--------------------------------------------------------------------------------

---------------------------------------------------------CCAACTGGCCCCTGGAGAAACTC

TCAAG---------------------------------------------------------------------------

--------------------------------------------------------------------------------

--------------------------------------------------------------------------------

--------------------------------------------------------------------------------

--------------------------------------------------------------------------------

--------------------------------------------------------------------------------

--------------------------------------------------------------------------------

--------------------------------------------------------------------------------

--------------------------------------------------------------------------------

--------------------------------------------------------------------------------

--------------------------------------------------------------------------------

--------------------------------------------------------------------------------

--------------------------------------------------------------------------------

--------------------------------------------------------------------------------

--------------------------------------------------------------------------------

--------------------------------------------------------------------------------

--------------------------------------------------------------------------------

--------------------------------------------------------------------------------

--------------------------------------------------------------------------------

--------------------------------------------------------------------------------

--------------------------------------------------------------------------------

--------------------------------------------------------------------------------

--------------------------------------------------------------------------------

--------------------------------------------------------------------------------

--------------------------------------------------------------------------------

----------------------------------------------------------

>Enam-ChirIF2

--------------------------------------------------------------------------------

--------------------------------------------------------------------------------

--------------------------------------------------------------------------------

--------------------------------------------------------------------------------

--------------------------------------------------------------------------------

--------------------------------------------------------------------------------

--------------------------------------------------------------------------------

--------------------------------------------------------------------------------

--------------------------------------------------------------------------------

--------------------------------------------------------------------------------

--------------------------------------------------------------------------------

--------------------------------------------------------------------------------

--------------------------------------------------------------------------------

-------------------------------------------------------CCTGCTGGAAGAAAYACTTGGAACC

RCCAAG--------------------------------------------------------------------------

--------------------------------------------------------------------------------

--------------------------------------------------------------------------------

--------------------------------------------------------------------------------

--------------------------------------------------------------------------------

--------------------------------------------------------------------------------

--------------------------------------------------------------------------------

--------------------------------------------------------------------------------

--------------------------------------------------------------------------------

--------------------------------------------------------------------------------

--------------------------------------------------------------------------------

--------------------------------------------------------------------------------

--------------------------------------------------------------------------------

--------------------------------------------------------------------------------

--------------------------------------------------------------------------------

--------------------------------------------------------------------------------

--------------------------------------------------------------------------------

--------------------------------------------------------------------------------

--------------------------------------------------------------------------------

--------------------------------------------------------------------------------

--------------------------------------------------------------------------------

----------------------------------------------------------

>Enam-ChirIR2

--------------------------------------------------------------------------------

--------------------------------------------------------------------------------

--------------------------------------------------------------------------------

--------------------------------------------------------------------------------

--------------------------------------------------------------------------------

--------------------------------------------------------------------------------

--------------------------------------------------------------------------------

--------------------------------------------------------------------------------

--------------------------------------------------------------------------------

--------------------------------------------------------------------------------

--------------------------------------------------------------------------------

--------------------------------------------------------------------------------

--------------------------------------------------------------------------------

--------------------------------------------------------------------------------

--------------------------------------------------------------------------------

---------------------------CCCTATGATCCCAGGGAAAACTCACCATAC-----------------------

--------------------------------------------------------------------------------

--------------------------------------------------------------------------------

--------------------------------------------------------------------------------

--------------------------------------------------------------------------------

--------------------------------------------------------------------------------

--------------------------------------------------------------------------------

--------------------------------------------------------------------------------

--------------------------------------------------------------------------------

--------------------------------------------------------------------------------

--------------------------------------------------------------------------------

--------------------------------------------------------------------------------

--------------------------------------------------------------------------------

--------------------------------------------------------------------------------

--------------------------------------------------------------------------------

--------------------------------------------------------------------------------

--------------------------------------------------------------------------------

--------------------------------------------------------------------------------

--------------------------------------------------------------------------------

--------------------------------------------------------------------------------

----------------------------------------------------------

>Enam-ChiropR3

--------------------------------------------------------------------------------

--------------------------------------------------------------------------------

--------------------------------------------------------------------------------

--------------------------------------------------------------------------------

--------------------------------------------------------------------------------

--------------------------------------------------------------------------------

--------------------------------------------------------------------------------

--------------------------------------------------------------------------------

--------------------------------------------------------------------------------

--------------------------------------------------------------------------------

--------------------------------------------------------------------------------

--------------------------------------------------------------------------------

--------------------------------------------------------------------------------

--------------------------------------------------------------------------------

--------------------------------------------------------------------------------

--------------------------------------------------------------------------------

--------------------------------------------------------------------------------

--------------------------------------------------------------------------------

--------------------------------------------------------------------------------

-------------------------CCCASGGARGATTTTCCTTATGGTGAATWTTACC---------------------

--------------------------------------------------------------------------------

--------------------------------------------------------------------------------

--------------------------------------------------------------------------------

--------------------------------------------------------------------------------

--------------------------------------------------------------------------------

--------------------------------------------------------------------------------

--------------------------------------------------------------------------------

--------------------------------------------------------------------------------

--------------------------------------------------------------------------------

--------------------------------------------------------------------------------

--------------------------------------------------------------------------------

--------------------------------------------------------------------------------

--------------------------------------------------------------------------------

--------------------------------------------------------------------------------

--------------------------------------------------------------------------------

----------------------------------------------------------

>Enam-ChiropR4

--------------------------------------------------------------------------------

--------------------------------------------------------------------------------

--------------------------------------------------------------------------------

--------------------------------------------------------------------------------

--------------------------------------------------------------------------------

--------------------------------------------------------------------------------

--------------------------------------------------------------------------------

--------------------------------------------------------------------------------

--------------------------------------------------------------------------------

--------------------------------------------------------------------------------

--------------------------------------------------------------------------------

--------------------------------------------------------------------------------

--------------------------------------------------------------------------------

--------------------------------------------------------------------------------

--------------------------------------------------------------------------------

--------------------------------------------------------------------------------

--------------------------------------------------------------------------------

--------------------------------------------------------------------------------

--------------------------------------------------------------------------------

----------------------------------------CCTTATGGTGAATTTTACCCCTGGAACSCAG---------

--------------------------------------------------------------------------------

--------------------------------------------------------------------------------

--------------------------------------------------------------------------------

--------------------------------------------------------------------------------

--------------------------------------------------------------------------------

--------------------------------------------------------------------------------

--------------------------------------------------------------------------------

--------------------------------------------------------------------------------

--------------------------------------------------------------------------------

--------------------------------------------------------------------------------

--------------------------------------------------------------------------------

--------------------------------------------------------------------------------

--------------------------------------------------------------------------------

--------------------------------------------------------------------------------

--------------------------------------------------------------------------------

----------------------------------------------------------

>Enam-ChiropF5

--------------------------------------------------------------------------------

--------------------------------------------------------------------------------

--------------------------------------------------------------------------------

--------------------------------------------------------------------------------

--------------------------------------------------------------------------------

--------------------------------------------------------------------------------

--------------------------------------------------------------------------------

--------------------------------------------------------------------------------

--------------------------------------------------------------------------------

--------------------------------------------------------------------------------

--------------------------------------------------------------------------------

--------------------------------------------------------------------------------

--------------------------------------------------------------------------------

--------------------------------------------------------------------------------

--------------------------------------------------------------------------------

--------------------------CCCCTATGATCCCAGGGAAAACTCACCATAC-----------------------

--------------------------------------------------------------------------------

--------------------------------------------------------------------------------

--------------------------------------------------------------------------------

--------------------------------------------------------------------------------

--------------------------------------------------------------------------------

--------------------------------------------------------------------------------

--------------------------------------------------------------------------------

--------------------------------------------------------------------------------

--------------------------------------------------------------------------------

--------------------------------------------------------------------------------

--------------------------------------------------------------------------------

--------------------------------------------------------------------------------

--------------------------------------------------------------------------------

--------------------------------------------------------------------------------

--------------------------------------------------------------------------------

--------------------------------------------------------------------------------

--------------------------------------------------------------------------------

--------------------------------------------------------------------------------

--------------------------------------------------------------------------------

----------------------------------------------------------

>Enam-ChiropF6

--------------------------------------------------------------------------------

--------------------------------------------------------------------------------

--------------------------------------------------------------------------------

--------------------------------------------------------------------------------

--------------------------------------------------------------------------------

--------------------------------------------------------------------------------

--------------------------------------------------------------------------------

--------------------------------------------------------------------------------

--------------------------------------------------------------------------------

--------------------------------------------------------------------------------

--------------------------------------------------------------------------------

--------------------------------------------------------------------------------

--------------------------------------------------------------------------------

--------------------------------------------------------------------------------

--------------------------------------------------------------------------------

----------------------------------------GGGAAAACTCACCATACMTYAGAAGCAATAC---------

--------------------------------------------------------------------------------

--------------------------------------------------------------------------------

--------------------------------------------------------------------------------

--------------------------------------------------------------------------------

--------------------------------------------------------------------------------

--------------------------------------------------------------------------------

--------------------------------------------------------------------------------

--------------------------------------------------------------------------------

--------------------------------------------------------------------------------

--------------------------------------------------------------------------------

--------------------------------------------------------------------------------

--------------------------------------------------------------------------------

--------------------------------------------------------------------------------

--------------------------------------------------------------------------------

--------------------------------------------------------------------------------

--------------------------------------------------------------------------------

--------------------------------------------------------------------------------

--------------------------------------------------------------------------------

--------------------------------------------------------------------------------

----------------------------------------------------------

>Enam-ChirIF3

--------------------------------------------------------------------------------

--------------------------------------------------------------------------------

--------------------------------------------------------------------------------

--------------------------------------------------------------------------------

--------------------------------------------------------------------------------

--------------------------------------------------------------------------------

--------------------------------------------------------------------------------

--------------------------------------------------------------------------------

--------------------------------------------------------------------------------

--------------------------------------------------------------------------------

--------------------------------------------------------------------------------

--------------------------------------------------------------------------------

--------------------------------------------------------------------------------

--------------------------------------------------------------------------------

--------------------------------------------------------------------------------

--------------------------------------------------------------------------------

--------------------------------------------------------------------------------

--------------------------------------------------------------------------------

--------------------------------------------------------------------------------

--------------------------------------------------------------------------------

--------------------------------------------------------------------------------

--------------------------------------------------------------------------------

----------------------------------------------CCTGCTAGSCCACCACACCAGAAAGAGAACC---

--------------------------------------------------------------------------------

--------------------------------------------------------------------------------

--------------------------------------------------------------------------------

--------------------------------------------------------------------------------

--------------------------------------------------------------------------------

--------------------------------------------------------------------------------

--------------------------------------------------------------------------------

--------------------------------------------------------------------------------

--------------------------------------------------------------------------------

--------------------------------------------------------------------------------

--------------------------------------------------------------------------------

--------------------------------------------------------------------------------

----------------------------------------------------------

>Enam-ChirIR3

--------------------------------------------------------------------------------

--------------------------------------------------------------------------------

--------------------------------------------------------------------------------

--------------------------------------------------------------------------------

--------------------------------------------------------------------------------

--------------------------------------------------------------------------------

--------------------------------------------------------------------------------

--------------------------------------------------------------------------------

--------------------------------------------------------------------------------

--------------------------------------------------------------------------------

--------------------------------------------------------------------------------

--------------------------------------------------------------------------------

--------------------------------------------------------------------------------

--------------------------------------------------------------------------------

--------------------------------------------------------------------------------

--------------------------------------------------------------------------------

--------------------------------------------------------------------------------

--------------------------------------------------------------------------------

--------------------------------------------------------------------------------

--------------------------------------------------------------------------------

--------------------------------------------------------------------------------

--------------------------------------------------------------------------------

--------------------------------------------------------------------------------

--------------------------------------------------------------------------------

------------------------------CAYTTGGCTTTCCAGGACTTAATTCCTCCAAG------------------

--------------------------------------------------------------------------------

--------------------------------------------------------------------------------

--------------------------------------------------------------------------------

--------------------------------------------------------------------------------

--------------------------------------------------------------------------------

--------------------------------------------------------------------------------

--------------------------------------------------------------------------------

--------------------------------------------------------------------------------

--------------------------------------------------------------------------------

--------------------------------------------------------------------------------

----------------------------------------------------------

>Enam-ChiropR5

--------------------------------------------------------------------------------

--------------------------------------------------------------------------------

--------------------------------------------------------------------------------

--------------------------------------------------------------------------------

--------------------------------------------------------------------------------

--------------------------------------------------------------------------------

--------------------------------------------------------------------------------

--------------------------------------------------------------------------------

--------------------------------------------------------------------------------

--------------------------------------------------------------------------------

--------------------------------------------------------------------------------

--------------------------------------------------------------------------------

--------------------------------------------------------------------------------

--------------------------------------------------------------------------------

--------------------------------------------------------------------------------

--------------------------------------------------------------------------------

--------------------------------------------------------------------------------

--------------------------------------------------------------------------------

--------------------------------------------------------------------------------

--------------------------------------------------------------------------------

--------------------------------------------------------------------------------

--------------------------------------------------------------------------------

--------------------------------------------------------------------------------

--------------------------------------------------------------------------------

--------------------------------------------------------------------------------

--------------------------------------------------------------------------------

--------------------------------------------------------------------------------

-------------------------------------------------------GAAACAGYTCAGAGAAARGACWGCC

TGG-----------------------------------------------------------------------------

--------------------------------------------------------------------------------

--------------------------------------------------------------------------------

--------------------------------------------------------------------------------

--------------------------------------------------------------------------------

--------------------------------------------------------------------------------

--------------------------------------------------------------------------------

----------------------------------------------------------

>Enam-ChiropR6

--------------------------------------------------------------------------------

--------------------------------------------------------------------------------

--------------------------------------------------------------------------------

--------------------------------------------------------------------------------

--------------------------------------------------------------------------------

--------------------------------------------------------------------------------

--------------------------------------------------------------------------------

--------------------------------------------------------------------------------

--------------------------------------------------------------------------------

--------------------------------------------------------------------------------

--------------------------------------------------------------------------------

--------------------------------------------------------------------------------

--------------------------------------------------------------------------------

--------------------------------------------------------------------------------

--------------------------------------------------------------------------------

--------------------------------------------------------------------------------

--------------------------------------------------------------------------------

--------------------------------------------------------------------------------

--------------------------------------------------------------------------------

--------------------------------------------------------------------------------

--------------------------------------------------------------------------------

--------------------------------------------------------------------------------

--------------------------------------------------------------------------------

--------------------------------------------------------------------------------

--------------------------------------------------------------------------------

--------------------------------------------------------------------------------

--------------------------------------------------------------------------------

--------------------------------------------------------------------GAAAAGACAGCC

TGGAGAAAGTCAAAACCC--------------------------------------------------------------

--------------------------------------------------------------------------------

--------------------------------------------------------------------------------

--------------------------------------------------------------------------------

--------------------------------------------------------------------------------

--------------------------------------------------------------------------------

--------------------------------------------------------------------------------

----------------------------------------------------------

>Enam-ChiropF7

--------------------------------------------------------------------------------

--------------------------------------------------------------------------------

--------------------------------------------------------------------------------

--------------------------------------------------------------------------------

--------------------------------------------------------------------------------

--------------------------------------------------------------------------------

--------------------------------------------------------------------------------

--------------------------------------------------------------------------------

--------------------------------------------------------------------------------

--------------------------------------------------------------------------------

--------------------------------------------------------------------------------

--------------------------------------------------------------------------------

--------------------------------------------------------------------------------

--------------------------------------------------------------------------------

--------------------------------------------------------------------------------

--------------------------------------------------------------------------------

--------------------------------------------------------------------------------

--------------------------------------------------------------------------------

--------------------------------------------------------------------------------

--------------------------------------------------------------------------------

--------------------------------------------------------------------------------

--------------------------------------------------------------------------------

--------------------------------------------------------------------------------

-----------------------GGGCTTCAGAAAAATCCAAYATGGCGTG-----------------------------

--------------------------------------------------------------------------------

--------------------------------------------------------------------------------

--------------------------------------------------------------------------------

--------------------------------------------------------------------------------

--------------------------------------------------------------------------------

--------------------------------------------------------------------------------

--------------------------------------------------------------------------------

--------------------------------------------------------------------------------

--------------------------------------------------------------------------------

--------------------------------------------------------------------------------

--------------------------------------------------------------------------------

----------------------------------------------------------

>Enam-ChiropF8

--------------------------------------------------------------------------------

--------------------------------------------------------------------------------

--------------------------------------------------------------------------------

--------------------------------------------------------------------------------

--------------------------------------------------------------------------------

--------------------------------------------------------------------------------

--------------------------------------------------------------------------------

--------------------------------------------------------------------------------

--------------------------------------------------------------------------------

--------------------------------------------------------------------------------

--------------------------------------------------------------------------------

--------------------------------------------------------------------------------

--------------------------------------------------------------------------------

--------------------------------------------------------------------------------

--------------------------------------------------------------------------------

--------------------------------------------------------------------------------

--------------------------------------------------------------------------------

--------------------------------------------------------------------------------

--------------------------------------------------------------------------------

--------------------------------------------------------------------------------

--------------------------------------------------------------------------------

--------------------------------------------------------------------------------

--------------------------------------------------------------------------------

--------------------------------------CCAAYATGGCGTGAAGGTGAGAATTTG---------------

--------------------------------------------------------------------------------

--------------------------------------------------------------------------------

--------------------------------------------------------------------------------

--------------------------------------------------------------------------------

--------------------------------------------------------------------------------

--------------------------------------------------------------------------------

--------------------------------------------------------------------------------

--------------------------------------------------------------------------------

--------------------------------------------------------------------------------

--------------------------------------------------------------------------------

--------------------------------------------------------------------------------

----------------------------------------------------------

>Enam-ChirIF4

--------------------------------------------------------------------------------

--------------------------------------------------------------------------------

--------------------------------------------------------------------------------

--------------------------------------------------------------------------------

--------------------------------------------------------------------------------

--------------------------------------------------------------------------------

--------------------------------------------------------------------------------

--------------------------------------------------------------------------------

--------------------------------------------------------------------------------

--------------------------------------------------------------------------------

--------------------------------------------------------------------------------

--------------------------------------------------------------------------------

--------------------------------------------------------------------------------

--------------------------------------------------------------------------------

--------------------------------------------------------------------------------

--------------------------------------------------------------------------------

--------------------------------------------------------------------------------

--------------------------------------------------------------------------------

--------------------------------------------------------------------------------

--------------------------------------------------------------------------------

--------------------------------------------------------------------------------

--------------------------------------------------------------------------------

--------------------------------------------------------------------------------

--------------------------------------------------------------------------------

--------------------------------------------------------------------------------

--------------------------------------------------------------------------------

--------------------------------------------------------------------------------

-----------------------------------------------------------------------------GCC

TGGAGAAAGTCAAAACCCAARTCCTTTTAG--------------------------------------------------

--------------------------------------------------------------------------------

--------------------------------------------------------------------------------

--------------------------------------------------------------------------------

--------------------------------------------------------------------------------

--------------------------------------------------------------------------------

--------------------------------------------------------------------------------

----------------------------------------------------------

>Enam-ChirIR4

--------------------------------------------------------------------------------

--------------------------------------------------------------------------------

--------------------------------------------------------------------------------

--------------------------------------------------------------------------------

--------------------------------------------------------------------------------

--------------------------------------------------------------------------------

--------------------------------------------------------------------------------

--------------------------------------------------------------------------------

--------------------------------------------------------------------------------

--------------------------------------------------------------------------------

--------------------------------------------------------------------------------

--------------------------------------------------------------------------------

--------------------------------------------------------------------------------

--------------------------------------------------------------------------------

--------------------------------------------------------------------------------

--------------------------------------------------------------------------------

--------------------------------------------------------------------------------

--------------------------------------------------------------------------------

--------------------------------------------------------------------------------

--------------------------------------------------------------------------------

--------------------------------------------------------------------------------

--------------------------------------------------------------------------------

--------------------------------------------------------------------------------

--------------------------------------------------------------------------------

--------------------------------------------------------------------------------

--------------------------------------------------------------------------------

--------------------------------------------------------------------------------

--------------------------------------------------------------------------------

--------------------------------------------------------------------------------

--------------------------------GAAGCCAGTTCCCTTCAWTCRAAGAATACACC----------------

--------------------------------------------------------------------------------

--------------------------------------------------------------------------------

--------------------------------------------------------------------------------

--------------------------------------------------------------------------------

--------------------------------------------------------------------------------

----------------------------------------------------------

>Enam-ChiropR7

--------------------------------------------------------------------------------

--------------------------------------------------------------------------------

--------------------------------------------------------------------------------

--------------------------------------------------------------------------------

--------------------------------------------------------------------------------

--------------------------------------------------------------------------------

--------------------------------------------------------------------------------

--------------------------------------------------------------------------------

--------------------------------------------------------------------------------

--------------------------------------------------------------------------------

--------------------------------------------------------------------------------

--------------------------------------------------------------------------------

--------------------------------------------------------------------------------

--------------------------------------------------------------------------------

--------------------------------------------------------------------------------

--------------------------------------------------------------------------------

--------------------------------------------------------------------------------

--------------------------------------------------------------------------------

--------------------------------------------------------------------------------

--------------------------------------------------------------------------------

--------------------------------------------------------------------------------

--------------------------------------------------------------------------------

--------------------------------------------------------------------------------

--------------------------------------------------------------------------------

--------------------------------------------------------------------------------

--------------------------------------------------------------------------------

--------------------------------------------------------------------------------

--------------------------------------------------------------------------------

--------------------------------------------------------------------------------

--------------------------------------------------------------------------------

--------------------------------------------------------------------------------

--------------------------------------------------------------------------------

--------------------------------------------------------------------------------

--------------------------------------------------------------------------------

----------------------------------------------------------GATGAACACAYTMCATTTGATT

CCC-------------------------------------------------------

>Enam-ChiropR8

--------------------------------------------------------------------------------

--------------------------------------------------------------------------------

--------------------------------------------------------------------------------

--------------------------------------------------------------------------------

--------------------------------------------------------------------------------

--------------------------------------------------------------------------------

--------------------------------------------------------------------------------

--------------------------------------------------------------------------------

--------------------------------------------------------------------------------

--------------------------------------------------------------------------------

--------------------------------------------------------------------------------

--------------------------------------------------------------------------------

--------------------------------------------------------------------------------

--------------------------------------------------------------------------------

--------------------------------------------------------------------------------

--------------------------------------------------------------------------------

--------------------------------------------------------------------------------

--------------------------------------------------------------------------------

--------------------------------------------------------------------------------

--------------------------------------------------------------------------------

--------------------------------------------------------------------------------

--------------------------------------------------------------------------------

--------------------------------------------------------------------------------

--------------------------------------------------------------------------------

--------------------------------------------------------------------------------

--------------------------------------------------------------------------------

--------------------------------------------------------------------------------

--------------------------------------------------------------------------------

--------------------------------------------------------------------------------

--------------------------------------------------------------------------------

--------------------------------------------------------------------------------

--------------------------------------------------------------------------------

--------------------------------------------------------------------------------

--------------------------------------------------------------------------------

------------------------------------------------------------------CAYTMCATTTGATT

CCCTTCAARTAGGGACC-----------------------------------------

**Marsupialia Primers**

>Monodelphis

AATCCTTATTTTGGCTATTTTGGATATCATGGATTTGGAGGGCGACCCCCATATTATTCAGAAGAGATGTTTGAACAAGA

CTTTGAAAAGCCCAAAGAGGAAGATCCCCCCAAAGTAGAGAGTACCACCGCAGCCCCTCCAACGAACTCCACGGTTCCTG

AGACCAATTCAACTCAAGCAACTGTCCCTGTCTCAGGAGGGAGTCAAGGTGGAAATGAGACCAGCACAACAGGGAATGAA

GCTGAAGTGCAGAACCCTGGGATCAACTTGGCTCCTGCTGTTAATGTCTCTGGCCAAGACGGACCAGGAAGTCAGGTTCC

CCCTGGCCCATATCAGCCAACTATTCTTGAAAACTCTCCAAATCCTAATTTAAGAGGCTTTCCCATAAGCAGACAATGGA

GTCAAACAAGCTTCCCTGTAGGACCTAGACTGATGGGCCCTTTTTATAGAAATTATCCCAATCAAAGGAGCTACCAATGG

CCCAGTTTAGCTTATGTAAGCAAGCAAATAGTTCGCCCAGGAAACACAGCTTACCAAAAAGTTTATCCTTACATTTCTAA

GAGCAATTCTCCTAATCATGTATCTAATCCAGCAAATAACAGAAGAAAACCCCAGGGTCCAACCAAACAACCTGAAGAAA

TAAATGGTGGGTTAGCAGGTCCCAAGCTTGGCACTGCCCATAAAGAGGAGCAGATTCAAAACCCCAAAGAGAATTCAGTA

ACCCAGAGAGAAAGAATAACCTTTCCTACAAGGGATCCAACCAGCACCTGGAGAAACTCACAAGGTTATGAAACCAACAA

ATCAAATTATAAATTAACTCCTCCACAGGGCAGCCCTTCAGTACCAAGTGTCAATTCTGTTGACCAGCCTGAAAATTATT

ACCCCAGGGTAGATTCCAGGAGATTCCCAGCTAGTATTCAAGCATCAAATTTTCCCAAAGGAATAGTTTCAGAACCAAGA

AAAGGTCCCTCTGACTCTGAAATTAATCCACCAGAAATAAAACATGGAACCCACCAGACTGATGAAGGTCCATATCCTCC

AAAAGAATCCTTTTTTCTGAGAAGCAATACATGGAATCTCCAAAAAGGATCTCCAATTTTTGAGGATGAACCTACAAAAC

AGGAAGGATCTTTACTCTATCCAGCCCTTGGAGCAAGAGAAAATGTTCCCTACCCTGAATACGTGCCCTATGATCCCCAG

AGGAGCTCTCTTTATGCCAGAGGTAATATGTGGGATGAAGGAGATGAGTTTCCTGGCACTTTCAGACCAGCAGGTCAACC

AGGAAATCCATTGCACTCCCTGAATTCTCCTTCAGGTCAGAGACATCGGTCAACTGACAATGAAGAGGATCCAATTGACC

CAACGGGTGATGAGTTTTACCGGGGACCTGATGCAGGGGGTAAGGAATCCAACTTCAAAGGAAGTCAAGTTAGGTACCGT

GAAAGATACCCATATGCCCCCAGTCATCCATCCAAGCCAAAGGAATACCCCCAACATTCCACAGATAATTTAGAAAGACA

AAGAAATCCGTCTTATGGTGAATATTATCCCTGGCACTTTCCACCTTACAATTTTGCTCCTCCTCTAACACCACCAAGAG

AAAACAGTGGTTATTATTATCCTCCTAACGCCTTGGAACAAGAAGAGAGTACACCATCTCCATCTTGGGGGTCATGGGAC

CAAAAGAATTATGTCCCAGCAAAAAAGGGAAGAATACCATATTATAATAGATATTTCTGGGATCAAGCCACCAATTTACA

CAAATCTGCAGCAAGTGCCCTGGAGCAGAGAGAACATCAACCCCATTCCAGTAGTTTTCCAGCTGGGCTTAGAGGAAGCC

TAACATACCAAGAAACAGAGAATCTGAACTATGACAGTGTACACAATAACAGGATAAATACTCCTGAAAAAGGACAGTTT

CCTGTCACTGACTCTGTGATTCAAAACAACCTGATATATCAAGGCGAAGTATATTCATATTCCCCAGCCAGCCAAAGAAG

TCCCTGCTGTGCTGGTGAATCCCCAGGGCCCAGAGAAAGCCCTCTCATTTCCCTCGATTACTCTCCACCCTTTGGTCTTG

TTTCAGGAGATGATGGAGAAAGAAATCCCCCAACTACGGAGGGCATCCACATGAAGCATGCGAGGCACATCATCTACTCC

ACAGACATCCAACCCAACCAAAGAAATAGTTCAGAGAAAAATCTATCTGAGAAAGGAGCAAATCCAGACCCTTTCAGGGC

TGACACAGCCATGTTGAAAAAAAGCTCACCTTGTTCTAAAAGGAGCCCAGCAGGCCAGACAGAAAGCGTGCCTTTTTCTG

AAGCTGACTCCCTCCAAGCAAACATGCCCTGCCGCAAAAGTAACCTTAGAGGAGAAGGGAACAATGTTCTTGCAAAAATC

TTGGGAGTCAACCAGTTCAATGCAAGAACCAGTAACCTTATCCCTGAGGAACTCGAGGCTCCTGAAGAAAATCTCGGGCC

AGAAAATATCCAAAGTGAAGGGGGAGGAAGTGAGGGTCGTGTGAAACAGAAAGGAGTGCCTAATATCCAGAAGGTGCCAT

GCCTCCACTCTAAACTAAAGGACCACCTCCTTTCCAGCACAGGTGCTCCACTTGGCAAGAGGAGACCAGGCCTGTCCACT

GGAGAGCCAGCCATGGTCTCTGCCCAGCCCAGCAGCACATTGACTGGATTGACAGCTAGGGAGCAGCTTGGTGGTACAAA

TATCGACCCACTCGTTGCCTCAGAGCCACCTCTTTCTTTTCCATCTCTTTCAATCCCAAAGAATGCAGAGTTTCAGGACT

GCCTGCTGCTCCACAGCTAGGG------------------------------------

>Enam-MarsF1

---CCTTATTTTGGCTATTTTGGATAYCAYGG------------------------------------------------

--------------------------------------------------------------------------------

--------------------------------------------------------------------------------

--------------------------------------------------------------------------------

--------------------------------------------------------------------------------

--------------------------------------------------------------------------------

--------------------------------------------------------------------------------

--------------------------------------------------------------------------------

--------------------------------------------------------------------------------

--------------------------------------------------------------------------------

--------------------------------------------------------------------------------

--------------------------------------------------------------------------------

--------------------------------------------------------------------------------

--------------------------------------------------------------------------------

--------------------------------------------------------------------------------

--------------------------------------------------------------------------------

--------------------------------------------------------------------------------

--------------------------------------------------------------------------------

--------------------------------------------------------------------------------

--------------------------------------------------------------------------------

--------------------------------------------------------------------------------

--------------------------------------------------------------------------------

--------------------------------------------------------------------------------

--------------------------------------------------------------------------------

--------------------------------------------------------------------------------

--------------------------------------------------------------------------------

--------------------------------------------------------------------------------

--------------------------------------------------------------------------------

--------------------------------------------------------------------------------

--------------------------------------------------------------------------------

--------------------------------------------------------------------------------

--------------------------------------------------------------------------------

--------------------------------------------------------------------------------

--------------------------------------------------------------------------------

--------------------------------------------------------------------------------

----------------------------------------------------------

>Enam-MarsF2

-------------GCTATTTTGGATAYCAYGGATTTGGAGGGC-------------------------------------

--------------------------------------------------------------------------------

--------------------------------------------------------------------------------

--------------------------------------------------------------------------------

--------------------------------------------------------------------------------

--------------------------------------------------------------------------------

--------------------------------------------------------------------------------

--------------------------------------------------------------------------------

--------------------------------------------------------------------------------

--------------------------------------------------------------------------------

--------------------------------------------------------------------------------

--------------------------------------------------------------------------------

--------------------------------------------------------------------------------

--------------------------------------------------------------------------------

--------------------------------------------------------------------------------

--------------------------------------------------------------------------------

--------------------------------------------------------------------------------

--------------------------------------------------------------------------------

--------------------------------------------------------------------------------

--------------------------------------------------------------------------------

--------------------------------------------------------------------------------

--------------------------------------------------------------------------------

--------------------------------------------------------------------------------

--------------------------------------------------------------------------------

--------------------------------------------------------------------------------

--------------------------------------------------------------------------------

--------------------------------------------------------------------------------

--------------------------------------------------------------------------------

--------------------------------------------------------------------------------

--------------------------------------------------------------------------------

--------------------------------------------------------------------------------

--------------------------------------------------------------------------------

--------------------------------------------------------------------------------

--------------------------------------------------------------------------------

--------------------------------------------------------------------------------

----------------------------------------------------------

>Enam-MarsIF1

--------------------------------------------------------------------------------

--------------------------------------------------------------------------------

--------------------------------------------------------------------------------

--------------------------------------------------------------------------------

----------------------------GAAAACTCTCCAAAYCCTAATTTWAGAGGC----------------------

--------------------------------------------------------------------------------

--------------------------------------------------------------------------------

--------------------------------------------------------------------------------

--------------------------------------------------------------------------------

--------------------------------------------------------------------------------

--------------------------------------------------------------------------------

--------------------------------------------------------------------------------

--------------------------------------------------------------------------------

--------------------------------------------------------------------------------

--------------------------------------------------------------------------------

--------------------------------------------------------------------------------

--------------------------------------------------------------------------------

--------------------------------------------------------------------------------

--------------------------------------------------------------------------------

--------------------------------------------------------------------------------

--------------------------------------------------------------------------------

--------------------------------------------------------------------------------

--------------------------------------------------------------------------------

--------------------------------------------------------------------------------

--------------------------------------------------------------------------------

--------------------------------------------------------------------------------

--------------------------------------------------------------------------------

--------------------------------------------------------------------------------

--------------------------------------------------------------------------------

--------------------------------------------------------------------------------

--------------------------------------------------------------------------------

--------------------------------------------------------------------------------

--------------------------------------------------------------------------------

--------------------------------------------------------------------------------

--------------------------------------------------------------------------------

----------------------------------------------------------

>Enam-MarsIR1

--------------------------------------------------------------------------------

--------------------------------------------------------------------------------

--------------------------------------------------------------------------------

--------------------------------------------------------------------------------

--------------------------------------------------------------------------------

--------------------------------------------------------------------------------

------------------------------------------------------------GTTTATCCTTACRTTTCYAA

GAGCAATTCTCC--------------------------------------------------------------------

--------------------------------------------------------------------------------

--------------------------------------------------------------------------------

--------------------------------------------------------------------------------

--------------------------------------------------------------------------------

--------------------------------------------------------------------------------

--------------------------------------------------------------------------------

--------------------------------------------------------------------------------

--------------------------------------------------------------------------------

--------------------------------------------------------------------------------

--------------------------------------------------------------------------------

--------------------------------------------------------------------------------

--------------------------------------------------------------------------------

--------------------------------------------------------------------------------

--------------------------------------------------------------------------------

--------------------------------------------------------------------------------

--------------------------------------------------------------------------------

--------------------------------------------------------------------------------

--------------------------------------------------------------------------------

--------------------------------------------------------------------------------

--------------------------------------------------------------------------------

--------------------------------------------------------------------------------

--------------------------------------------------------------------------------

--------------------------------------------------------------------------------

--------------------------------------------------------------------------------

--------------------------------------------------------------------------------

--------------------------------------------------------------------------------

--------------------------------------------------------------------------------

----------------------------------------------------------

>Enam-MarsR1

--------------------------------------------------------------------------------

--------------------------------------------------------------------------------

--------------------------------------------------------------------------------

--------------------------------------------------------------------------------

--------------------------------------------------------------------------------

--------------------------------------------------------------------------------

--------------------------------------------------------------------------------

--------------------------------------------------------------------------------

--------------------------------------------------------------------------------

--------------------------------------------------------------------------------

--------------------------------------------------------------------------------

---------------------------------GTATWCAARCATCAAATTTTCCCAAAGG-------------------

--------------------------------------------------------------------------------

--------------------------------------------------------------------------------

--------------------------------------------------------------------------------

--------------------------------------------------------------------------------

--------------------------------------------------------------------------------

--------------------------------------------------------------------------------

--------------------------------------------------------------------------------

--------------------------------------------------------------------------------

--------------------------------------------------------------------------------

--------------------------------------------------------------------------------

--------------------------------------------------------------------------------

--------------------------------------------------------------------------------

--------------------------------------------------------------------------------

--------------------------------------------------------------------------------

--------------------------------------------------------------------------------

--------------------------------------------------------------------------------

--------------------------------------------------------------------------------

--------------------------------------------------------------------------------

--------------------------------------------------------------------------------

--------------------------------------------------------------------------------

--------------------------------------------------------------------------------

--------------------------------------------------------------------------------

--------------------------------------------------------------------------------

----------------------------------------------------------

>Enam-MarsR2

--------------------------------------------------------------------------------

--------------------------------------------------------------------------------

--------------------------------------------------------------------------------

--------------------------------------------------------------------------------

--------------------------------------------------------------------------------

--------------------------------------------------------------------------------

--------------------------------------------------------------------------------

--------------------------------------------------------------------------------

--------------------------------------------------------------------------------

--------------------------------------------------------------------------------

--------------------------------------------------------------------------------

---------------------------------------------CAAATTTTCCCAAAGGAATAGTTTCAGAACC----

--------------------------------------------------------------------------------

--------------------------------------------------------------------------------

--------------------------------------------------------------------------------

--------------------------------------------------------------------------------

--------------------------------------------------------------------------------

--------------------------------------------------------------------------------

--------------------------------------------------------------------------------

--------------------------------------------------------------------------------

--------------------------------------------------------------------------------

--------------------------------------------------------------------------------

--------------------------------------------------------------------------------

--------------------------------------------------------------------------------

--------------------------------------------------------------------------------

--------------------------------------------------------------------------------

--------------------------------------------------------------------------------

--------------------------------------------------------------------------------

--------------------------------------------------------------------------------

--------------------------------------------------------------------------------

--------------------------------------------------------------------------------

--------------------------------------------------------------------------------

--------------------------------------------------------------------------------

--------------------------------------------------------------------------------

--------------------------------------------------------------------------------

----------------------------------------------------------

>Enam-MarsF3

--------------------------------------------------------------------------------

--------------------------------------------------------------------------------

--------------------------------------------------------------------------------

--------------------------------------------------------------------------------

--------------------------------------------------------------------------------

--------------------------------------------------------------------------------

--------------------------------------------------------------------------------

--------------------------------------------------------------------------------

--------------------------------------------------------------------------------

------------------------CCTACAAGGGWTCCAACCAGCMCCTGG-----------------------------

--------------------------------------------------------------------------------

--------------------------------------------------------------------------------

--------------------------------------------------------------------------------

--------------------------------------------------------------------------------

--------------------------------------------------------------------------------

--------------------------------------------------------------------------------

--------------------------------------------------------------------------------

--------------------------------------------------------------------------------

--------------------------------------------------------------------------------

--------------------------------------------------------------------------------

--------------------------------------------------------------------------------

--------------------------------------------------------------------------------

--------------------------------------------------------------------------------

--------------------------------------------------------------------------------

--------------------------------------------------------------------------------

--------------------------------------------------------------------------------

--------------------------------------------------------------------------------

--------------------------------------------------------------------------------

--------------------------------------------------------------------------------

--------------------------------------------------------------------------------

--------------------------------------------------------------------------------

--------------------------------------------------------------------------------

--------------------------------------------------------------------------------

--------------------------------------------------------------------------------

--------------------------------------------------------------------------------

----------------------------------------------------------

>Enam-MarsF4

--------------------------------------------------------------------------------

--------------------------------------------------------------------------------

--------------------------------------------------------------------------------

--------------------------------------------------------------------------------

--------------------------------------------------------------------------------

--------------------------------------------------------------------------------

--------------------------------------------------------------------------------

--------------------------------------------------------------------------------

--------------------------------------------------------------------------------

------------------------------------CCAACCAGCMCCTGGAGAAACTCACAAGGTTATG----------

--------------------------------------------------------------------------------

--------------------------------------------------------------------------------

--------------------------------------------------------------------------------

--------------------------------------------------------------------------------

--------------------------------------------------------------------------------

--------------------------------------------------------------------------------

--------------------------------------------------------------------------------

--------------------------------------------------------------------------------

--------------------------------------------------------------------------------

--------------------------------------------------------------------------------

--------------------------------------------------------------------------------

--------------------------------------------------------------------------------

--------------------------------------------------------------------------------

--------------------------------------------------------------------------------

--------------------------------------------------------------------------------

--------------------------------------------------------------------------------

--------------------------------------------------------------------------------

--------------------------------------------------------------------------------

--------------------------------------------------------------------------------

--------------------------------------------------------------------------------

--------------------------------------------------------------------------------

--------------------------------------------------------------------------------

--------------------------------------------------------------------------------

--------------------------------------------------------------------------------

--------------------------------------------------------------------------------

----------------------------------------------------------

>Enam-MarsIF2

--------------------------------------------------------------------------------

--------------------------------------------------------------------------------

--------------------------------------------------------------------------------

--------------------------------------------------------------------------------

--------------------------------------------------------------------------------

--------------------------------------------------------------------------------

--------------------------------------------------------------------------------

--------------------------------------------------------------------------------

--------------------------------------------------------------------------------

--------------------------------------------------------------------------------

--------------------------------------------------------------------------------

--------------------------------------------------------------------------------

--------------------------------------------------------------------------------

--------------------------------GGAATCYCCAAMAAGGATMTCCAATTTTTGAGG---------------

--------------------------------------------------------------------------------

--------------------------------------------------------------------------------

--------------------------------------------------------------------------------

--------------------------------------------------------------------------------

--------------------------------------------------------------------------------

--------------------------------------------------------------------------------

--------------------------------------------------------------------------------

--------------------------------------------------------------------------------

--------------------------------------------------------------------------------

--------------------------------------------------------------------------------

--------------------------------------------------------------------------------

--------------------------------------------------------------------------------

--------------------------------------------------------------------------------

--------------------------------------------------------------------------------

--------------------------------------------------------------------------------

--------------------------------------------------------------------------------

--------------------------------------------------------------------------------

--------------------------------------------------------------------------------

--------------------------------------------------------------------------------

--------------------------------------------------------------------------------

--------------------------------------------------------------------------------

----------------------------------------------------------

>Enam-MarsIR2

--------------------------------------------------------------------------------

--------------------------------------------------------------------------------

--------------------------------------------------------------------------------

--------------------------------------------------------------------------------

--------------------------------------------------------------------------------

--------------------------------------------------------------------------------

--------------------------------------------------------------------------------

--------------------------------------------------------------------------------

--------------------------------------------------------------------------------

--------------------------------------------------------------------------------

--------------------------------------------------------------------------------

--------------------------------------------------------------------------------

--------------------------------------------------------------------------------

--------------------------------------------------------------------------------

--------------------------------------------------------------------------------

----------------------------------------------------------------------CAGGYCAASC

AGGAAAYCCATTAYACTCCCTG----------------------------------------------------------

--------------------------------------------------------------------------------

--------------------------------------------------------------------------------

--------------------------------------------------------------------------------

--------------------------------------------------------------------------------

--------------------------------------------------------------------------------

--------------------------------------------------------------------------------

--------------------------------------------------------------------------------

--------------------------------------------------------------------------------

--------------------------------------------------------------------------------

--------------------------------------------------------------------------------

--------------------------------------------------------------------------------

--------------------------------------------------------------------------------

--------------------------------------------------------------------------------

--------------------------------------------------------------------------------

--------------------------------------------------------------------------------

--------------------------------------------------------------------------------

--------------------------------------------------------------------------------

--------------------------------------------------------------------------------

----------------------------------------------------------

>Enam-MarsR3

--------------------------------------------------------------------------------

--------------------------------------------------------------------------------

--------------------------------------------------------------------------------

--------------------------------------------------------------------------------

--------------------------------------------------------------------------------

--------------------------------------------------------------------------------

--------------------------------------------------------------------------------

--------------------------------------------------------------------------------

--------------------------------------------------------------------------------

--------------------------------------------------------------------------------

--------------------------------------------------------------------------------

--------------------------------------------------------------------------------

--------------------------------------------------------------------------------

--------------------------------------------------------------------------------

--------------------------------------------------------------------------------

--------------------------------------------------------------------------------

--------------------------------------------------------------------------------

--------------------------------------------------------------------------------

--------------------------------------------------------------------------------

--------------------------------------------------------------------------------

-----------------------------------------------------CCATCTCCATCTTGGGGGTCATGGGAC

CAAAAG--------------------------------------------------------------------------

--------------------------------------------------------------------------------

--------------------------------------------------------------------------------

--------------------------------------------------------------------------------

--------------------------------------------------------------------------------

--------------------------------------------------------------------------------

--------------------------------------------------------------------------------

--------------------------------------------------------------------------------

--------------------------------------------------------------------------------

--------------------------------------------------------------------------------

--------------------------------------------------------------------------------

--------------------------------------------------------------------------------

--------------------------------------------------------------------------------

--------------------------------------------------------------------------------

----------------------------------------------------------

>Enam-MarsR4

--------------------------------------------------------------------------------

--------------------------------------------------------------------------------

--------------------------------------------------------------------------------

--------------------------------------------------------------------------------

--------------------------------------------------------------------------------

--------------------------------------------------------------------------------

--------------------------------------------------------------------------------

--------------------------------------------------------------------------------

--------------------------------------------------------------------------------

--------------------------------------------------------------------------------

--------------------------------------------------------------------------------

--------------------------------------------------------------------------------

--------------------------------------------------------------------------------

--------------------------------------------------------------------------------

--------------------------------------------------------------------------------

--------------------------------------------------------------------------------

--------------------------------------------------------------------------------

--------------------------------------------------------------------------------

--------------------------------------------------------------------------------

--------------------------------------------------------------------------------

----------------------------------------------------------------------GTCATGGGAC

CAAAAGAATTATGTCCCAGC------------------------------------------------------------

--------------------------------------------------------------------------------

--------------------------------------------------------------------------------

--------------------------------------------------------------------------------

--------------------------------------------------------------------------------

--------------------------------------------------------------------------------

--------------------------------------------------------------------------------

--------------------------------------------------------------------------------

--------------------------------------------------------------------------------

--------------------------------------------------------------------------------

--------------------------------------------------------------------------------

--------------------------------------------------------------------------------

--------------------------------------------------------------------------------

--------------------------------------------------------------------------------

----------------------------------------------------------

>Enam-MarsF5

--------------------------------------------------------------------------------

--------------------------------------------------------------------------------

--------------------------------------------------------------------------------

--------------------------------------------------------------------------------

--------------------------------------------------------------------------------

--------------------------------------------------------------------------------

--------------------------------------------------------------------------------

--------------------------------------------------------------------------------

--------------------------------------------------------------------------------

--------------------------------------------------------------------------------

--------------------------------------------------------------------------------

--------------------------------------------------------------------------------

--------------------------------------------------------------------------------

--------------------------------------------------------------------------------

--------------------------------------------------------------------------------

--------------------------------------------------------------------------------

--------------------------------------------------------------------------------

--------------------------------------------------------------------------------

-----------------------------------GCCAAAGGAATACCCCCARCATTCCAC------------------

--------------------------------------------------------------------------------

--------------------------------------------------------------------------------

--------------------------------------------------------------------------------

--------------------------------------------------------------------------------

--------------------------------------------------------------------------------

--------------------------------------------------------------------------------

--------------------------------------------------------------------------------

--------------------------------------------------------------------------------

--------------------------------------------------------------------------------

--------------------------------------------------------------------------------

--------------------------------------------------------------------------------

--------------------------------------------------------------------------------

--------------------------------------------------------------------------------

--------------------------------------------------------------------------------

--------------------------------------------------------------------------------

--------------------------------------------------------------------------------

----------------------------------------------------------

>Enam-MarsF6

--------------------------------------------------------------------------------

--------------------------------------------------------------------------------

--------------------------------------------------------------------------------

--------------------------------------------------------------------------------

--------------------------------------------------------------------------------

--------------------------------------------------------------------------------

--------------------------------------------------------------------------------

--------------------------------------------------------------------------------

--------------------------------------------------------------------------------

--------------------------------------------------------------------------------

--------------------------------------------------------------------------------

--------------------------------------------------------------------------------

--------------------------------------------------------------------------------

--------------------------------------------------------------------------------

--------------------------------------------------------------------------------

--------------------------------------------------------------------------------

--------------------------------------------------------------------------------

--------------------------------------------------------------------------------

-------------------------------------------------CCCARCATTCCACAAATAACTTASMAAGAC-

--------------------------------------------------------------------------------

--------------------------------------------------------------------------------

--------------------------------------------------------------------------------

--------------------------------------------------------------------------------

--------------------------------------------------------------------------------

--------------------------------------------------------------------------------

--------------------------------------------------------------------------------

--------------------------------------------------------------------------------

--------------------------------------------------------------------------------

--------------------------------------------------------------------------------

--------------------------------------------------------------------------------

--------------------------------------------------------------------------------

--------------------------------------------------------------------------------

--------------------------------------------------------------------------------

--------------------------------------------------------------------------------

--------------------------------------------------------------------------------

----------------------------------------------------------

>Enam-MarsIF3

--------------------------------------------------------------------------------

--------------------------------------------------------------------------------

--------------------------------------------------------------------------------

--------------------------------------------------------------------------------

--------------------------------------------------------------------------------

--------------------------------------------------------------------------------

--------------------------------------------------------------------------------

--------------------------------------------------------------------------------

--------------------------------------------------------------------------------

--------------------------------------------------------------------------------

--------------------------------------------------------------------------------

--------------------------------------------------------------------------------

--------------------------------------------------------------------------------

--------------------------------------------------------------------------------

--------------------------------------------------------------------------------

--------------------------------------------------------------------------------

--------------------------------------------------------------------------------

--------------------------------------------------------------------------------

--------------------------------------------------------------------------------

--------------------------------------------------------------------------------

--------------------------------------------------------------------------------

--------------------------------------------------------------------------------

----------------------------------------CCCCWTTCCAGCAGYTTTCCAGYTGRGCTTAG--------

--------------------------------------------------------------------------------

--------------------------------------------------------------------------------

--------------------------------------------------------------------------------

--------------------------------------------------------------------------------

--------------------------------------------------------------------------------

--------------------------------------------------------------------------------

--------------------------------------------------------------------------------

--------------------------------------------------------------------------------

--------------------------------------------------------------------------------

--------------------------------------------------------------------------------

--------------------------------------------------------------------------------

--------------------------------------------------------------------------------

----------------------------------------------------------

>Enam-MarsIR3

--------------------------------------------------------------------------------

--------------------------------------------------------------------------------

--------------------------------------------------------------------------------

--------------------------------------------------------------------------------

--------------------------------------------------------------------------------

--------------------------------------------------------------------------------

--------------------------------------------------------------------------------

--------------------------------------------------------------------------------

--------------------------------------------------------------------------------

--------------------------------------------------------------------------------

--------------------------------------------------------------------------------

--------------------------------------------------------------------------------

--------------------------------------------------------------------------------

--------------------------------------------------------------------------------

--------------------------------------------------------------------------------

--------------------------------------------------------------------------------

--------------------------------------------------------------------------------

--------------------------------------------------------------------------------

--------------------------------------------------------------------------------

--------------------------------------------------------------------------------

--------------------------------------------------------------------------------

--------------------------------------------------------------------------------

--------------------------------------------------------------------------------

--------------------------------------------------------------------------------

-------------------------------------------------------------------CCAKCCAAAGAAG

TCCCTGCTGTGCTGGTG---------------------------------------------------------------

--------------------------------------------------------------------------------

--------------------------------------------------------------------------------

--------------------------------------------------------------------------------

--------------------------------------------------------------------------------

--------------------------------------------------------------------------------

--------------------------------------------------------------------------------

--------------------------------------------------------------------------------

--------------------------------------------------------------------------------

--------------------------------------------------------------------------------

----------------------------------------------------------

>Enam-MarsR5

--------------------------------------------------------------------------------

--------------------------------------------------------------------------------

--------------------------------------------------------------------------------

--------------------------------------------------------------------------------

--------------------------------------------------------------------------------

--------------------------------------------------------------------------------

--------------------------------------------------------------------------------

--------------------------------------------------------------------------------

--------------------------------------------------------------------------------

--------------------------------------------------------------------------------

--------------------------------------------------------------------------------

--------------------------------------------------------------------------------

--------------------------------------------------------------------------------

--------------------------------------------------------------------------------

--------------------------------------------------------------------------------

--------------------------------------------------------------------------------

--------------------------------------------------------------------------------

--------------------------------------------------------------------------------

--------------------------------------------------------------------------------

--------------------------------------------------------------------------------

--------------------------------------------------------------------------------

--------------------------------------------------------------------------------

--------------------------------------------------------------------------------

--------------------------------------------------------------------------------

--------------------------------------------------------------------------------

--------------------------------------------------------------------------------

--------------------------------------------------------------------------------

-------------CCAACCAAMGAAATAGTTCAGAGAAAAATC-------------------------------------

--------------------------------------------------------------------------------

--------------------------------------------------------------------------------

--------------------------------------------------------------------------------

--------------------------------------------------------------------------------

--------------------------------------------------------------------------------

--------------------------------------------------------------------------------

--------------------------------------------------------------------------------

----------------------------------------------------------

>Enam-MarsR6

--------------------------------------------------------------------------------

--------------------------------------------------------------------------------

--------------------------------------------------------------------------------

--------------------------------------------------------------------------------

--------------------------------------------------------------------------------

--------------------------------------------------------------------------------

--------------------------------------------------------------------------------

--------------------------------------------------------------------------------

--------------------------------------------------------------------------------

--------------------------------------------------------------------------------

--------------------------------------------------------------------------------

--------------------------------------------------------------------------------

--------------------------------------------------------------------------------

--------------------------------------------------------------------------------

--------------------------------------------------------------------------------

--------------------------------------------------------------------------------

--------------------------------------------------------------------------------

--------------------------------------------------------------------------------

--------------------------------------------------------------------------------

--------------------------------------------------------------------------------

--------------------------------------------------------------------------------

--------------------------------------------------------------------------------

--------------------------------------------------------------------------------

--------------------------------------------------------------------------------

--------------------------------------------------------------------------------

--------------------------------------------------------------------------------

--------------------------------------------------------------------------------

----------------------------GTTCAGAGAAAAATCYAYCTGAGAAAGGAG----------------------

--------------------------------------------------------------------------------

--------------------------------------------------------------------------------

--------------------------------------------------------------------------------

--------------------------------------------------------------------------------

--------------------------------------------------------------------------------

--------------------------------------------------------------------------------

--------------------------------------------------------------------------------

----------------------------------------------------------

>Enam-MarsF7

--------------------------------------------------------------------------------

--------------------------------------------------------------------------------

--------------------------------------------------------------------------------

--------------------------------------------------------------------------------

--------------------------------------------------------------------------------

--------------------------------------------------------------------------------

--------------------------------------------------------------------------------

--------------------------------------------------------------------------------

--------------------------------------------------------------------------------

--------------------------------------------------------------------------------

--------------------------------------------------------------------------------

--------------------------------------------------------------------------------

--------------------------------------------------------------------------------

--------------------------------------------------------------------------------

--------------------------------------------------------------------------------

--------------------------------------------------------------------------------

--------------------------------------------------------------------------------

--------------------------------------------------------------------------------

--------------------------------------------------------------------------------

--------------------------------------------------------------------------------

--------------------------------------------------------------------------------

--------------------------------------------------------------------------------

--------------------------------------------------------------------------------

--------------------------------------------------------------------------------

-------------------------------------------------------CATATYCCCCAGCCAKCCAAAGAAG

TCCCTGCTG-----------------------------------------------------------------------

--------------------------------------------------------------------------------

--------------------------------------------------------------------------------

--------------------------------------------------------------------------------

--------------------------------------------------------------------------------

--------------------------------------------------------------------------------

--------------------------------------------------------------------------------

--------------------------------------------------------------------------------

--------------------------------------------------------------------------------

--------------------------------------------------------------------------------

----------------------------------------------------------

>Enam-MarsF8

--------------------------------------------------------------------------------

--------------------------------------------------------------------------------

--------------------------------------------------------------------------------

--------------------------------------------------------------------------------

--------------------------------------------------------------------------------

--------------------------------------------------------------------------------

--------------------------------------------------------------------------------

--------------------------------------------------------------------------------

--------------------------------------------------------------------------------

--------------------------------------------------------------------------------

--------------------------------------------------------------------------------

--------------------------------------------------------------------------------

--------------------------------------------------------------------------------

--------------------------------------------------------------------------------

--------------------------------------------------------------------------------

--------------------------------------------------------------------------------

--------------------------------------------------------------------------------

--------------------------------------------------------------------------------

--------------------------------------------------------------------------------

--------------------------------------------------------------------------------

--------------------------------------------------------------------------------

--------------------------------------------------------------------------------

--------------------------------------------------------------------------------

--------------------------------------------------------------------------------

-----------------------------------------------------------------------CCAAAGAAG

TCCCTGCTGTGCTGGTG---------------------------------------------------------------

--------------------------------------------------------------------------------

--------------------------------------------------------------------------------

--------------------------------------------------------------------------------

--------------------------------------------------------------------------------

--------------------------------------------------------------------------------

--------------------------------------------------------------------------------

--------------------------------------------------------------------------------

--------------------------------------------------------------------------------

--------------------------------------------------------------------------------

----------------------------------------------------------

>Enam-MarsIF4

--------------------------------------------------------------------------------

--------------------------------------------------------------------------------

--------------------------------------------------------------------------------

--------------------------------------------------------------------------------

--------------------------------------------------------------------------------

--------------------------------------------------------------------------------

--------------------------------------------------------------------------------

--------------------------------------------------------------------------------

--------------------------------------------------------------------------------

--------------------------------------------------------------------------------

--------------------------------------------------------------------------------

--------------------------------------------------------------------------------

--------------------------------------------------------------------------------

--------------------------------------------------------------------------------

--------------------------------------------------------------------------------

--------------------------------------------------------------------------------

--------------------------------------------------------------------------------

--------------------------------------------------------------------------------

--------------------------------------------------------------------------------

--------------------------------------------------------------------------------

--------------------------------------------------------------------------------

--------------------------------------------------------------------------------

--------------------------------------------------------------------------------

--------------------------------------------------------------------------------

--------------------------------------------------------------------------------

--------------------------------------------------------------------------------

--------------------------------------------------------------------------------

--------------------------------------------------------------------------------

-----------------------------------------------------------------------CTTTTTCTG

AAGCYGAYTCCCTCCAAGCAAAC---------------------------------------------------------

--------------------------------------------------------------------------------

--------------------------------------------------------------------------------

--------------------------------------------------------------------------------

--------------------------------------------------------------------------------

--------------------------------------------------------------------------------

----------------------------------------------------------

>Enam-MarsIR4

--------------------------------------------------------------------------------

--------------------------------------------------------------------------------

--------------------------------------------------------------------------------

--------------------------------------------------------------------------------

--------------------------------------------------------------------------------

--------------------------------------------------------------------------------

--------------------------------------------------------------------------------

--------------------------------------------------------------------------------

--------------------------------------------------------------------------------

--------------------------------------------------------------------------------

--------------------------------------------------------------------------------

--------------------------------------------------------------------------------

--------------------------------------------------------------------------------

--------------------------------------------------------------------------------

--------------------------------------------------------------------------------

--------------------------------------------------------------------------------

--------------------------------------------------------------------------------

--------------------------------------------------------------------------------

--------------------------------------------------------------------------------

--------------------------------------------------------------------------------

--------------------------------------------------------------------------------

--------------------------------------------------------------------------------

--------------------------------------------------------------------------------

--------------------------------------------------------------------------------

--------------------------------------------------------------------------------

--------------------------------------------------------------------------------

--------------------------------------------------------------------------------

--------------------------------------------------------------------------------

--------------------------------------------------------------------------------

--------------------------------------------------------------------------------

-----------------------------------CCTTATYCCTGAGGAACTCGAGGCYCCTGAAG-------------

--------------------------------------------------------------------------------

--------------------------------------------------------------------------------

--------------------------------------------------------------------------------

--------------------------------------------------------------------------------

----------------------------------------------------------

>Enam-MarsR7

--------------------------------------------------------------------------------

--------------------------------------------------------------------------------

--------------------------------------------------------------------------------

--------------------------------------------------------------------------------

--------------------------------------------------------------------------------

--------------------------------------------------------------------------------

--------------------------------------------------------------------------------

--------------------------------------------------------------------------------

--------------------------------------------------------------------------------

--------------------------------------------------------------------------------

--------------------------------------------------------------------------------

--------------------------------------------------------------------------------

--------------------------------------------------------------------------------

--------------------------------------------------------------------------------

--------------------------------------------------------------------------------

--------------------------------------------------------------------------------

--------------------------------------------------------------------------------

--------------------------------------------------------------------------------

--------------------------------------------------------------------------------

--------------------------------------------------------------------------------

--------------------------------------------------------------------------------

--------------------------------------------------------------------------------

--------------------------------------------------------------------------------

--------------------------------------------------------------------------------

--------------------------------------------------------------------------------

--------------------------------------------------------------------------------

--------------------------------------------------------------------------------

--------------------------------------------------------------------------------

--------------------------------------------------------------------------------

--------------------------------------------------------------------------------

--------------------------------------------------------------------------------

--------------------------------------------------------------------------------

--------------------------------------------------------------------------------

--------------------------------------------------------------------------------

--------------------------------------------------CAATCCCAAAGAATGCAGAGTTTCAGGAC-

----------------------------------------------------------

>Enam-MarsR8

--------------------------------------------------------------------------------

--------------------------------------------------------------------------------

--------------------------------------------------------------------------------

--------------------------------------------------------------------------------

--------------------------------------------------------------------------------

--------------------------------------------------------------------------------

--------------------------------------------------------------------------------

--------------------------------------------------------------------------------

--------------------------------------------------------------------------------

--------------------------------------------------------------------------------

--------------------------------------------------------------------------------

--------------------------------------------------------------------------------

--------------------------------------------------------------------------------

--------------------------------------------------------------------------------

--------------------------------------------------------------------------------

--------------------------------------------------------------------------------

--------------------------------------------------------------------------------

--------------------------------------------------------------------------------

--------------------------------------------------------------------------------

--------------------------------------------------------------------------------

--------------------------------------------------------------------------------

--------------------------------------------------------------------------------

--------------------------------------------------------------------------------

--------------------------------------------------------------------------------

--------------------------------------------------------------------------------

--------------------------------------------------------------------------------

--------------------------------------------------------------------------------

--------------------------------------------------------------------------------

--------------------------------------------------------------------------------

--------------------------------------------------------------------------------

--------------------------------------------------------------------------------

--------------------------------------------------------------------------------

--------------------------------------------------------------------------------

--------------------------------------------------------------------------------

----------------------------------------------------------------GCAGAGTTTCAGGACT

GCCTGCTGCTCCACAGC-----------------------------------------

**Eulipotyphla Primers**

>Erinaceous_europaeus_F6

ACAGAATCCCTACTTTGGCTACTTTGGATTTCATGGATTTGGGGGTCGTCCTCCTTATTATTCAGAAGAGATGTTTGAAC

AGGATTTTGAAAAACCCAAAGAAAAAGATCCTCCTAAAACCGAGAGTCCAGCCACAGAACCCTCACCTAACTCAACTACT

CCTGAAACTAATTCTACCCAACCAAATGCACCCAATCCCAGAGGGAATCAGCCTGGAAACGACACCAATCCAGCTGGAAA

CGGTGGCCCTGGGTCAAACACTGTGAACAACCCTGCTGTTCAAAACCCATCCTCTACAGTTAACATTTCAGGCCAGGGAA

TACCAAGAAGTCAAATACCCCTGGGACGAAGGCAGCCAAATTTTCATGGAAATTATCCAAATCCCAACATCCGAAATTAT

CCTGCAGGAAGACAATGGCGTCCCACTGGTACAATCCTGGGTCATAGGCAGAACTGGCCATTCTACAGAACTCAACAAGT

TCAAGGGAGTCCTCGGTGGCACTCATTTGCTTTGGAAAACAAACAAGCACTTCGTCCAGGAACTCCATTTTACCGCAAAG

CTTATGCTTCCACTGCAAGAGGCAATTCTCCCAATCAGGCAGGAAATCCAGCAAATTTCAAAAGAAAGCCTCAGACTCCA

AATAAACAACCTATTGGAACCAGTGTTGCCCCTGTGGCTCCCAAACGTGATCCTGTTGGTCACAATGAAAAAATCCAGAA

TCCAAGAGAGAATTCACTAGGTCAGAAAGAAAGGATTGTCATTCCAACTAGAGACCCAACTGGCCCCTGGAGAAACTCTC

AAGATTATGGAATTAATAAATCAAATTTTAAGCCTTCTCGCCCTGAGGGTAACATGCCAGTTCCAAATTTTAATTCTATT

GATCAACATGAAAACTCTTATTACCCAAGAGGAGATTCCAGAAGAGCCCCAAATTCTGATGGACAAGCCCCAAGCCAGAA

TTTGCCCAAAGGGATTGTTTTAGAGCCAAAAAGAATTCCATATGAATCAGAAACTAATGGTCCAGAATTAAAACAGAATA

CATATCCGCCTGTCTACCTTGAGGAAATCCCTCCCCCTGAAAGAGAACTCTTTCCTGTTGGAAGAAATACTTGGAATCAC

CAAGAAATCTCTCCACCTTTTAAGGAAGACCCTAGGAGGCAGGAGGAACATTTGCCTCGTCCTTCCCAAGGCTCTAGGGG

AGGTGTTTACTATCCTGACTATAACCCCTATACCCCCAGAGAGAATGTCCCATATCATAGAAGCAATACATGGGATGAGA

GAGATGATTCTCCCAATACCGTAAGGCAGCCTGAAAATCCACTCTACCCCATGAATACTCCAGACCCCAAAGATACAGTA

CCTTATAATGAAGAGGACCCAGCTGACCCAACTGGAGATGAAACTTACCTAGGACAAAGTCAATGGGGCATGGAAGAGTC

AAACTTTAAAGGAGGGCCAACGGTTAGGCAATATGAAAGGGAACAATTTACTTCAAATCAACCAAAAGACTATCTTCCCT

ATCCCTTGGATAATCCATCAAAACCTAGGGAGGAGTTTCCTTACAATGAATTTTACCCCTGGAATCCAGATGAGACTTTC

CCATCATATAATACAGTTCCCTCTGTTCCACCACCTGTGGAGAGCAGGGGCTATTATGCTAGCAATGCTATTGGACAAGA

AGAAAGTTCTCCATTTCCTTCATGGAACTCCTGGACCAACAGAATTCAAGCCCAAGGGCAGAAAGAAAGAGATCCATATT

TTAACAGGAATTTCTGGGATCAGTCAACAAATGTATACAATGCTCCCACAAATCCACCACACCAGAAGGAAAACCAACCC

TATTCTAGTAACTACCCAGCTGGGCTTCAGAAAAATCCTACATGGAATGAAGGTGAGAATTTGAATTATGGCATGCAAAA

TACTAGGCTAAATTCACCAGAAAGAGAGCATTCTTCTTTCCCAGACTTAATTCCTTCAAATCACCCAGCTGGTCAAAAAG

AACCGCATTCATTCCACCAAACCCAGAGAGGCCCTTGCTGTGCCAGTGACCCCACAAGGCACAAGGACAATCCACTTGCT

CTTCAAGACTACACGCCATCCTTTGATCTTGCACCAGAGGAGAACCAAGATACCAGCCCTATGTACACAGAAGATAGTCA

TACTAAGCATGCAAGACATACCATCTCCCCAACAAGCAGCCTGCCTGGGCAAAGAAACAGCTCAGAGAAAAGACCACCTA

CAGAAAGTCAAAACCCAAGTCCTTTCAGAGAAGATGTGTCCACTCCGAGAAGGAACACACCATGTTCCATGAAGAATCAG

CTGGGTCAAAGGGGAATAATGCCCTTTCCAGAAGCCAGTTCCCTTCAGTCAAAGAATATACCTTGTCTCAAGAGTGACAT

TGTAGGAGATGGAAATAATCTTGTTTTGGAACAAATATTTGAAGACAACCAGCTCAATGTTGGCCTTACTCCTGAGCAGC

TTGTTATGGACACAGCTGAGCAAGACCCAAAGCCAGAAGGAATTCAAGGTGAAGTGCAAGTAAATGAGGGTGAACGACAG

CAATCAAGACCATCTAGCATATTACAGCTACCATGCTTTGACTCAAGGTTAGCAAAGTATCACTCATCTGACATTGGAAC

ACCACCTACCATTGGAAGGCAAACTTCATTTGATGGGGATCCAATTATGCCTACTGAAATACCCAACTCACTGACCGGAT

TAGCTACTGGGGAACAGTTTCAGAGCATAAATGTAGATACACAAAATACAGATGAACACCCTCCATTTGATTCCCTTCAA

ATAGGAACCAAYCCACAGGACCAGGTACAAGACTGCTTGCTACTTCAGGCCTAGGA--

>Enam-EulipF1

-------CCYTACTTYGGMTWCTTTGGATTTCATGG--------------------------------------------

--------------------------------------------------------------------------------

--------------------------------------------------------------------------------

--------------------------------------------------------------------------------

--------------------------------------------------------------------------------

--------------------------------------------------------------------------------

--------------------------------------------------------------------------------

--------------------------------------------------------------------------------

--------------------------------------------------------------------------------

--------------------------------------------------------------------------------

--------------------------------------------------------------------------------

--------------------------------------------------------------------------------

--------------------------------------------------------------------------------

--------------------------------------------------------------------------------

--------------------------------------------------------------------------------

--------------------------------------------------------------------------------

--------------------------------------------------------------------------------

--------------------------------------------------------------------------------

--------------------------------------------------------------------------------

--------------------------------------------------------------------------------

--------------------------------------------------------------------------------

--------------------------------------------------------------------------------

--------------------------------------------------------------------------------

--------------------------------------------------------------------------------

--------------------------------------------------------------------------------

--------------------------------------------------------------------------------

--------------------------------------------------------------------------------

--------------------------------------------------------------------------------

--------------------------------------------------------------------------------

--------------------------------------------------------------------------------

--------------------------------------------------------------------------------

--------------------------------------------------------------------------------

--------------------------------------------------------------------------------

--------------------------------------------------------------------------------

--------------------------------------------------------------------------------

----------------------------------------------------------

>Enam-EulipF2

----------------GGMTWCTTTGGATTTCATGGATTTGGGGG-----------------------------------

--------------------------------------------------------------------------------

--------------------------------------------------------------------------------

--------------------------------------------------------------------------------

--------------------------------------------------------------------------------

--------------------------------------------------------------------------------

--------------------------------------------------------------------------------

--------------------------------------------------------------------------------

--------------------------------------------------------------------------------

--------------------------------------------------------------------------------

--------------------------------------------------------------------------------

--------------------------------------------------------------------------------

--------------------------------------------------------------------------------

--------------------------------------------------------------------------------

--------------------------------------------------------------------------------

--------------------------------------------------------------------------------

--------------------------------------------------------------------------------

--------------------------------------------------------------------------------

--------------------------------------------------------------------------------

--------------------------------------------------------------------------------

--------------------------------------------------------------------------------

--------------------------------------------------------------------------------

--------------------------------------------------------------------------------

--------------------------------------------------------------------------------

--------------------------------------------------------------------------------

--------------------------------------------------------------------------------

--------------------------------------------------------------------------------

--------------------------------------------------------------------------------

--------------------------------------------------------------------------------

--------------------------------------------------------------------------------

--------------------------------------------------------------------------------

--------------------------------------------------------------------------------

--------------------------------------------------------------------------------

--------------------------------------------------------------------------------

--------------------------------------------------------------------------------

----------------------------------------------------------

>Enam-EulipIF1

--------------------------------------------------------------------------------

--------------------------------------------------------------------------------

--------------------------------------------------------------------------------

--------------------------------------------------------------------------------

--------------------------------------------------------------------------------

---GCAGGAAGACAATGGCGTCCCACTGGTAC------------------------------------------------

--------------------------------------------------------------------------------

--------------------------------------------------------------------------------

--------------------------------------------------------------------------------

--------------------------------------------------------------------------------

--------------------------------------------------------------------------------

--------------------------------------------------------------------------------

--------------------------------------------------------------------------------

--------------------------------------------------------------------------------

--------------------------------------------------------------------------------

--------------------------------------------------------------------------------

--------------------------------------------------------------------------------

--------------------------------------------------------------------------------

--------------------------------------------------------------------------------

--------------------------------------------------------------------------------

--------------------------------------------------------------------------------

--------------------------------------------------------------------------------

--------------------------------------------------------------------------------

--------------------------------------------------------------------------------

--------------------------------------------------------------------------------

--------------------------------------------------------------------------------

--------------------------------------------------------------------------------

--------------------------------------------------------------------------------

--------------------------------------------------------------------------------

--------------------------------------------------------------------------------

--------------------------------------------------------------------------------

--------------------------------------------------------------------------------

--------------------------------------------------------------------------------

--------------------------------------------------------------------------------

--------------------------------------------------------------------------------

----------------------------------------------------------

>Enam-EulipIR1

--------------------------------------------------------------------------------

--------------------------------------------------------------------------------

--------------------------------------------------------------------------------

--------------------------------------------------------------------------------

--------------------------------------------------------------------------------

--------------------------------------------------------------------------------

--------------------------------------------------------------------------------

------------------GAGGCAATTCTCCCAATCARGCAGGAAATCC-------------------------------

--------------------------------------------------------------------------------

--------------------------------------------------------------------------------

--------------------------------------------------------------------------------

--------------------------------------------------------------------------------

--------------------------------------------------------------------------------

--------------------------------------------------------------------------------

--------------------------------------------------------------------------------

--------------------------------------------------------------------------------

--------------------------------------------------------------------------------

--------------------------------------------------------------------------------

--------------------------------------------------------------------------------

--------------------------------------------------------------------------------

--------------------------------------------------------------------------------

--------------------------------------------------------------------------------

--------------------------------------------------------------------------------

--------------------------------------------------------------------------------

--------------------------------------------------------------------------------

--------------------------------------------------------------------------------

--------------------------------------------------------------------------------

--------------------------------------------------------------------------------

--------------------------------------------------------------------------------

--------------------------------------------------------------------------------

--------------------------------------------------------------------------------

--------------------------------------------------------------------------------

--------------------------------------------------------------------------------

--------------------------------------------------------------------------------

--------------------------------------------------------------------------------

----------------------------------------------------------

>Enam-EulipR1

--------------------------------------------------------------------------------

--------------------------------------------------------------------------------

--------------------------------------------------------------------------------

--------------------------------------------------------------------------------

--------------------------------------------------------------------------------

--------------------------------------------------------------------------------

--------------------------------------------------------------------------------

--------------------------------------------------------------------------------

--------------------------------------------------------------------------------

--------------------------------------------------------------------------------

--------------------------------------------------------------------------------

-------------------------------------------------CAAATTCTGATGGACAARCCCMAAGCCAG--

--------------------------------------------------------------------------------

--------------------------------------------------------------------------------

--------------------------------------------------------------------------------

--------------------------------------------------------------------------------

--------------------------------------------------------------------------------

--------------------------------------------------------------------------------

--------------------------------------------------------------------------------

--------------------------------------------------------------------------------

--------------------------------------------------------------------------------

--------------------------------------------------------------------------------

--------------------------------------------------------------------------------

--------------------------------------------------------------------------------

--------------------------------------------------------------------------------

--------------------------------------------------------------------------------

--------------------------------------------------------------------------------

--------------------------------------------------------------------------------

--------------------------------------------------------------------------------

--------------------------------------------------------------------------------

--------------------------------------------------------------------------------

--------------------------------------------------------------------------------

--------------------------------------------------------------------------------

--------------------------------------------------------------------------------

--------------------------------------------------------------------------------

----------------------------------------------------------

>Enam-EulipR2

--------------------------------------------------------------------------------

--------------------------------------------------------------------------------

--------------------------------------------------------------------------------

--------------------------------------------------------------------------------

--------------------------------------------------------------------------------

--------------------------------------------------------------------------------

--------------------------------------------------------------------------------

--------------------------------------------------------------------------------

--------------------------------------------------------------------------------

--------------------------------------------------------------------------------

--------------------------------------------------------------------------------

---------------------------------------------------------------CAARCCCMAAGCCAGAA

TTTGCCCAAAGGG-------------------------------------------------------------------

--------------------------------------------------------------------------------

--------------------------------------------------------------------------------

--------------------------------------------------------------------------------

--------------------------------------------------------------------------------

--------------------------------------------------------------------------------

--------------------------------------------------------------------------------

--------------------------------------------------------------------------------

--------------------------------------------------------------------------------

--------------------------------------------------------------------------------

--------------------------------------------------------------------------------

--------------------------------------------------------------------------------

--------------------------------------------------------------------------------

--------------------------------------------------------------------------------

--------------------------------------------------------------------------------

--------------------------------------------------------------------------------

--------------------------------------------------------------------------------

--------------------------------------------------------------------------------

--------------------------------------------------------------------------------

--------------------------------------------------------------------------------

--------------------------------------------------------------------------------

--------------------------------------------------------------------------------

--------------------------------------------------------------------------------

----------------------------------------------------------

>Enam-EulipF3

--------------------------------------------------------------------------------

--------------------------------------------------------------------------------

--------------------------------------------------------------------------------

--------------------------------------------------------------------------------

--------------------------------------------------------------------------------

--------------------------------------------------------------------------------

--------------------------------------------------------------------------------

--------------------------------------------------------------------------------

--------------------------------------------------------------------------------

----------------------------------------------------GACCCAACTGGCCCCTGGAGAAAYTCTC

AAG-----------------------------------------------------------------------------

--------------------------------------------------------------------------------

--------------------------------------------------------------------------------

--------------------------------------------------------------------------------

--------------------------------------------------------------------------------

--------------------------------------------------------------------------------

--------------------------------------------------------------------------------

--------------------------------------------------------------------------------

--------------------------------------------------------------------------------

--------------------------------------------------------------------------------

--------------------------------------------------------------------------------

--------------------------------------------------------------------------------

--------------------------------------------------------------------------------

--------------------------------------------------------------------------------

--------------------------------------------------------------------------------

--------------------------------------------------------------------------------

--------------------------------------------------------------------------------

--------------------------------------------------------------------------------

--------------------------------------------------------------------------------

--------------------------------------------------------------------------------

--------------------------------------------------------------------------------

--------------------------------------------------------------------------------

--------------------------------------------------------------------------------

--------------------------------------------------------------------------------

--------------------------------------------------------------------------------

----------------------------------------------------------

>Enam-EulipF4

--------------------------------------------------------------------------------

--------------------------------------------------------------------------------

--------------------------------------------------------------------------------

--------------------------------------------------------------------------------

--------------------------------------------------------------------------------

--------------------------------------------------------------------------------

--------------------------------------------------------------------------------

--------------------------------------------------------------------------------

--------------------------------------------------------------------------------

----------------------------------------------------------------CCCTGGAGAAAYTCTC

AAGATYWTGG----------------------------------------------------------------------

--------------------------------------------------------------------------------

--------------------------------------------------------------------------------

--------------------------------------------------------------------------------

--------------------------------------------------------------------------------

--------------------------------------------------------------------------------

--------------------------------------------------------------------------------

--------------------------------------------------------------------------------

--------------------------------------------------------------------------------

--------------------------------------------------------------------------------

--------------------------------------------------------------------------------

--------------------------------------------------------------------------------

--------------------------------------------------------------------------------

--------------------------------------------------------------------------------

--------------------------------------------------------------------------------

--------------------------------------------------------------------------------

--------------------------------------------------------------------------------

--------------------------------------------------------------------------------

--------------------------------------------------------------------------------

--------------------------------------------------------------------------------

--------------------------------------------------------------------------------

--------------------------------------------------------------------------------

--------------------------------------------------------------------------------

--------------------------------------------------------------------------------

--------------------------------------------------------------------------------

----------------------------------------------------------

>Enam-EulipIF2

--------------------------------------------------------------------------------

--------------------------------------------------------------------------------

--------------------------------------------------------------------------------

--------------------------------------------------------------------------------

--------------------------------------------------------------------------------

--------------------------------------------------------------------------------

--------------------------------------------------------------------------------

--------------------------------------------------------------------------------

--------------------------------------------------------------------------------

--------------------------------------------------------------------------------

--------------------------------------------------------------------------------

--------------------------------------------------------------------------------

--------------------------------------------------------------------------------

------------------------------------------------------------------------GGAAYCAY

CAAGAAATCTCTCCAYCTTTTAAGG-------------------------------------------------------

--------------------------------------------------------------------------------

--------------------------------------------------------------------------------

--------------------------------------------------------------------------------

--------------------------------------------------------------------------------

--------------------------------------------------------------------------------

--------------------------------------------------------------------------------

--------------------------------------------------------------------------------

--------------------------------------------------------------------------------

--------------------------------------------------------------------------------

--------------------------------------------------------------------------------

--------------------------------------------------------------------------------

--------------------------------------------------------------------------------

--------------------------------------------------------------------------------

--------------------------------------------------------------------------------

--------------------------------------------------------------------------------

--------------------------------------------------------------------------------

--------------------------------------------------------------------------------

--------------------------------------------------------------------------------

--------------------------------------------------------------------------------

--------------------------------------------------------------------------------

----------------------------------------------------------

>Enam-EulipIR2

--------------------------------------------------------------------------------

--------------------------------------------------------------------------------

--------------------------------------------------------------------------------

--------------------------------------------------------------------------------

--------------------------------------------------------------------------------

--------------------------------------------------------------------------------

--------------------------------------------------------------------------------

--------------------------------------------------------------------------------

--------------------------------------------------------------------------------

--------------------------------------------------------------------------------

--------------------------------------------------------------------------------

--------------------------------------------------------------------------------

--------------------------------------------------------------------------------

--------------------------------------------------------------------------------

--------------------------------------------------------------------------------

-----------------------------------------------------------------------GGGATGAGA

GAKATGRTYCTCCCAATAC-------------------------------------------------------------

--------------------------------------------------------------------------------

--------------------------------------------------------------------------------

--------------------------------------------------------------------------------

--------------------------------------------------------------------------------

--------------------------------------------------------------------------------

--------------------------------------------------------------------------------

--------------------------------------------------------------------------------
[truncated: 42,191 more chars]
