# Supplementary material for: Molecular Decay of the Tooth Gene Enamelin (ENAM) Mirrors the Loss of Enamel in the Fossil Record of Placental Mammals
Source: PLoS Genet. 2009 Sep 4;5(9):e1000634. doi: 10.1371/journal.pgen.1000634 (PMC2728479; doi:10.1371/journal.pgen.1000634)
Supplement: Table S1 — Comprehensive list of frameshift insertions and deletions in edentulous and enamelless taxa. Numbering of insertions and deletions corresponds to the alignment in Dataset S1. Numbers in red font indicate the minimum to maximum number of frameshift indels. (0.03 MB DOC) [file pgen.1000634.s011.doc]

# PHOLIDOTA (Pangolins)

***Manis*:** 1344-1348 (D),1362 (D), 2688-2697 (I) [3-8]

***Manis pentadactyla*:** 397 (I*), 561 (D*), 619-625 (D*), 652-653 (D*), 763 (D*), 1527 (D), 2842 (D), 3556-3557 (I), 3994 (D) [4-9]

* region not sequenced for *M. tricuspis*; indels could be on *M. pentadactyla* branch or *Manis* branch

***Manis tricuspus*:** 785 (I), 826 (D), 898-915 (D), 917 (D), 1011-1014 (D), 2735-2738 (I), 3491 (D), 3499 (D), 3537-3540 (I), 3945 (I) [10]

# ORYCTEROPODIDAE (Aardvark)

***Orycteropus***: 2649 (I), 3678 (D), 4041 (D) [3]

# PILOSA (Sloths and Anteaters)

**Pilosa:** 2503 (D), 2956-3479 (I*) [2-4]

*numerous indels that are not in multiples of three are nested within this insert

# VERMILINGUA (Anteaters)

**Vermilingua:** 434 (D*), 461 (D*), 1618 (D), 2291 (D), 2837-2841 (D) [3-7]

*region not sequencd for sloths; indels could be in common ancestor of Vermilingua or common ancestor of Pilosa

***Cyclopes*:** 11 (D), 51 (I), 149 (D), 200-201 (D; convergent deletion in *Tamandua* using deltran reconstruction), 444-445 (D), 489 (I), 612 (I), 616 (D), 622 (D), 870 (D), 1209-1212 (D*), 1288-1300 (D), 1486 (D), 1524 (D), 2272 (D), 2383 (I), 2786 (D), 2807 (D), 2864 (D), 2869-2870 (D) [20]

*four bp deletion that occurs within the 27 bp “Pilosa” insert

**Myrmecophagidae (*Myrmecophaga* + *Tamandua*):** 25 (D), 67 (I), 245 (D), 375-376 (I), 557 (D), 642 (D), 874 (D), 922 (I), 1118-1119 (D), 1340-1346 (D), 2200-2206 (D), 2320-2323 (D), 2382 (D), 2408 (D), 2424 (D), 2552-2553 (D), 2895 (D), 3722 (I*) [17-19]

*not sequenced in this region for *Cyclopes*; insertion could be in common ancestor of Myrmecophagidae or common ancestor of Vermilingua

***Myrmecophaga*:** 1182 (I), 1407 (I), 2796-2800 (D; spans five bp in alignment, but the deletion is four bp relative to taxa that are in frame), 3674-3684 (D; spans 11 bp in alignment, but the deletion is ten bp relative to taxa that are in frame), 3724 (D), 3742 (D), 3760-3761 (I), 3775-3776 (D), 3870-3880 (D*) [8-9]

*region not sequenced in *Tamandua* and *Cyclopes*; deletion could be in ancestor of *Myrmecophaga*, Myrmecophagidae, or Vermilingua)

***Tamandua*:** 5 (I), 200-201 (D; convergent deletion in *Cyclopes* based on deltran reconstruction), 2308-2309 (D), 2806-2807 (D), 3577 (I), 3677-3684 (D; spans 8 bp in alignment, but the deletion is seven bp relative to taxa that are in frame), 3701 (I), 3706 (I), 3713 (I), 3716 (I), 3738 (I) [11]

# FOLIVORA (Sloths)

**Folivora:** 1587-1588 (D), 2749-2755 (D), 2799 (I), 2805 (I), 3684 (I), 3883-3884 (I), 3494 (D) [7-12]

***Choloepus*:** 1419 (D*), 1481-1482 (I*), 2913 (D*) [0-5]

*region not sequenced for *Bradypus*, indels could be in common ancestor of *Choloepus* or common ancestor of Folivora

***Choloepus didactylus*:** 1541 (I), 2244-2251 (D) [2]

***Choloepus hoffmanni*:** 676 (D*), 943 (D*) [0-2]

*region not sequenced for *C. didactylus* and *Bradypus*; indels could be on *C. hoffmani* branch, in the common ancestor of *Choloepus*, in common ancestor of Folivora.

***Bradypus***: 2172-2173 (I), 2836-2837 (D) [2]

# CINGULATA (Armadillos)

***Euphractus* + *Chaetophractus*:** 321-340 (D), 800-801 (I), 923-932 (D), 1072 (D), 1483 (D) [5]

***Chaetophractus*:** 73 (D) [1]

***Euphractus*:** 154 (I) [1]

***Tolypeutes*:** 35 (I*), 652 (D), 696 (D), 1411-1412 (D), 1414 (D), 1589 (D), 3786 (D) [7]

* region sequenced in *Chaetophractus* but not *Euphractus*

***Dasypus*:** 4020 (I) [1]

# CETACEA (Whales)

***Kogia*:** 2343 (D), 4034-4035 (D) [2]

***Kogia simus*:** 2260-2261 (D) [1]

***Eschrichtius*:** 1243 (D; convergent in *Caperea* based on deltran reconstruction) [1]

***Caperea*:** 661 (D), 1243 (D; convergent in *Eschrichtius* based on deltran reconstruction) [2]
