# Supplementary material for: Molecular Decay of the Tooth Gene Enamelin (ENAM) Mirrors the Loss of Enamel in the Fossil Record of Placental Mammals
Source: PLoS Genet. 2009 Sep 4;5(9):e1000634. doi: 10.1371/journal.pgen.1000634 (PMC2728479; doi:10.1371/journal.pgen.1000634)
Supplement: Text S1 — Supplementary note on enamel in (a) sperm whales and (b) xenarthrans. (0.07 MB DOC) [file pgen.1000634.s012.doc]

**a) Enamel in sperm whales.** True prismatic enamel includes rods (prisms) and interprismatic crystallites. Rods form during the secretory phase of enamel formation. Each rod is a parallel bundle of ~10,000 hydroxyapatite crystallites and is the product of a single ameloblast [1]. Interprismatic crystallites form in the spaces between rods during the maturation phase of enamel formation. Pseudoprismatic enamel includes rods but not interprismatic enamel [2]. The enamel in *Physeter macrocephalus* is thin and pseudoprismatic based on microscopic analysis [3]. Bloodworth and Odell [4, p. 4] note in their Mammalian Species account for *Kogia breviceps* that “enamel has been found in very young individuals but likely wears away quickly” and cite Plon’s [5] unpublished Ph.D. dissertation. However, as noted by Simpson [6] in his discussion of enamel in the cingulatan *Utaetus*, it can be difficult or impossible to distinguish dense shiny dentin from enamel without thin sections. Further, experiments with ENAM^-/-^ knock-in mice demonstrate the presence of a very thin mineral layer, not to be confused with true enamel, that caps dentin [1]. Thus, the mere presence of a layer capping dentin cannot be taken as evidence of true enamel without further microscopic analysis. Our finding that there are two frameshift mutations in *Kogia breviceps*, which result in eight stop codons, provides evidence that a full-length enamelin protein cannot be manufactured in this species. It remains possible that a truncated version of enamelin is secreted and that with or without this protein product there is a degenerate “enamel” layer, perhaps having similarities to amelogenesis imperfectas in humans, that caps dentin and is quickly worn away.

**b) Enamel in living and fossil xenarthrans.** Some authors have suggested that enamel is absent in all extant xenarthrans [7, 8], but studies from the early 20^th^ century suggest that enamel is present in the nine-banded armadillo (*Dasypus novemcinctus*). Spurgin [9] concluded that enamel is present in the milk teeth of *D. novemcinctus*; Martin [10] believed that enamel is present in both the milk teeth and permanent teeth of this species. However, these studies do not provide decisive evidence for true prismatic enamel as opposed to aprismatic enamel or simply a mineralized dentin cap as described by Hu et al. [1] for ENAM^-/-^ knock-in mice. Spurgin [9] and Martin [10] document an enamel organ and an ameloblast-derived secretion that covers dentin, but the composition and structure of this secretion is unclear. Martin [10, p. 659] stated that “in favorable specimens, as shown in figure 4, the enamel is sometimes seen to be composed of darker areas which have separated from each other, and between which lighter areas appear. I have interpreted these darker areas as representing the enamel prisms…. Decalcification destroys all prismatic structure, so that examination under a polarizing microscope is useless. Attempts to grind down the calcified teeth have so far been unsuccessful because of the extreme thinness of the enamel coat, which causes it to break off in the process of grinding…. Therefore, since I have no evidence against the conclusion that the substance is enamel, and since it is secreted through the agency of Tomes’ processes, and looks like the newly formed enamel in the cat’s tooth, I feel that I am justified in applying the term enamel to the secretion which covers the dentine in the teeth of the armadillo.” Martin’s [10] figure 10 illustrates what she interprets as the enamel of a permanent tooth a few months after birth. Simpson [11, p. 3] reported the occurrence of enamel on some of the lower adult teeth of the early Eocene *Utaetus buccatus* based on thin sections: “The most striking feature is the presence of true enamel on the permanent teeth. This has been examined microscopically in thin sections, leaving no question as to its identification. It is thin, but typical of the simplest types of mammalian enamel. In the limited material available for microscopic study, the enamel prisms appear to be straight, parallel, and simple, without any striking or unusual characters.” In contrast to Martin [10], who argued that enamel occurs on the milk and adult teeth of *D. novemcinctus*, Simpson [6, p. 82] notes that *Utaetus* is the only xenarthran with true enamel on the permanent functional teeth, although “vestiges have been discovered on the germs of milk teeth of recent armadillos.” Future studies are required to elucidate the nature of the ‘enamel’ that covers the milk teeth, and possibly adult teeth, of *D. novemcinctus*. We identified only one frameshift in *D. novemcinctus* *ENAM*, which results in a premature stop codon. Whereas this frameshift precludes a functional, full-length enamelin protein, its location is relatively late in the coding sequence of exon 10 (alignment position 4020 in Dataset S1) and shorter protein products are possible. Given that the carboxy terminus of intact enamelin is rapidly processed after secretion and that only N-terminal cleavage products accumulate in deeper layers of enamel, a truncated version of enamelin may contribute to the formation of a vestigial ‘enamel’ layer that covers the dentin of some teeth in *D. novemcinctus* and is quickly worn off. Our finding that the protein-coding regions of three other EMP genes (*AMELX*, *AMBN*, *MMP20*) are intact (Figure S6) in *D. novemcinctus* also supports the hypothesis that vestigial ‘enamel’ is present in this species. In contrast to *D. novemcinctus*, which has only a single frameshift that occurs near the 3’ end of exon 10, frameshifts and premature stop codons occur much further upstream in *ENAM* of the other armadillos (*Tolypeutes*, *Chaetophractus*, *Euphractus*).

**References**

1. Hu, JC-C, et al. (2008) Enamel defects and ameloblast-specific expression in Enam knock-out/lacZ knock-in mice. J Biol Chem 283:10858-10871.

2. Line RPL, Novaes PD (2005) The development and evolution of mammalian enamel: structural and functional aspects. Braz J Morphol Sci 22:67-72.

3. Ishiyama M (1987) Enamel structure in odontocete whales. Scanning Microscopy 1:1071-1079.

4. Bloodworth BE, Odell DK (2008) *Kogia breviceps* (Cetacea: Kogiidae). Mamm Species 819:1-12.

5. Plon S (2004) The status and natural history of pygmy (*Kogia breviceps*) and dwarf (*Kogia sima*) sperm whales off southern Africa, Unpublished Ph.D. dissertation (Rhodes University, Grahamstown, South Africa).

6. Simpson GG (1948) The beginning of the age of mammals in South America. Bull Amer Mus Nat Hist 91:1-232.

7. Vizcaino SF, De Iuliis G (2003) Evidence for advanced carnivory in fossil armadillos (Mammalia: Xenarthra: Dasypodidae). Paleobiology 29:123-138.

8. McDonald HG (2003) Xenarthran skeletal anatomy: primitive or derived? Senckenbergianna biológica 83:5-18.

9. Spurgin AM (1904) Enamel in the teeth of an embryo edentate (*Dasypus novemcinctus* Linn). Amer J Anat 3:75-84.

10. Martin BE (1916) Tooth development in *Dasypus novemcinctus*. J Morphol 27:647-681.

11. Simpson GG (1932) Enamel on the teeth of an Eocene edentate. Amer Mus Novitat 567:1-4.
